# Cleptoparasitic bees (Apoidea, Anthophila) in rural protected areas in Latium (Italy): a faunistic survey through an integrative taxonomic approach 

**DOI:** 10.3897/BDJ.14.e193040

**Published:** 2026-07-14

**Authors:** Matteo Annessi, Alessandra Riccieri, Carlo Polidori, Maurizio Mei, Andrea Di Giulio

**Affiliations:** 1 Department of Science, Roma Tre University, Rome, Italy Department of Science, Roma Tre University Rome Italy; 2 NBFC - National Biodiversity Future Center, Piazza Marina 61, Palermo, Italy NBFC - National Biodiversity Future Center, Piazza Marina 61 Palermo Italy; 3 Department of Environmental Science and Policy (ESP), University of Milan, Milan, Italy Department of Environmental Science and Policy (ESP), University of Milan Milan Italy; 4 c/o Department of Biology and Biotechnology “Charles Darwin”, Sapienza University of Rome, Rome, Italy c/o Department of Biology and Biotechnology “Charles Darwin”, Sapienza University of Rome Rome Italy

**Keywords:** COI-5P, diversity, DNA barcoding, Mediterranean region, morphological identification

## Abstract

**Background:**

Cleptoparasitic bees represent a poorly studied yet ecologically important component of wild bee communities, responding sensitively to environmental heterogeneity and reflecting local wild bee diversity. Despite representing approximately 13% of bee species globally, significant knowledge gaps remain regarding their species distribution, taxonomy, and conservation status, particularly within the Mediterranean region, which is recognized as a global hotspot for bee diversity.

**New information:**

This work provides a faunistic survey of cleptoparasitic bees collected in rural protected areas within the province of Rome (Latium, central Italy), employing an integrative taxonomic approach combining morphological identification and DNA barcoding (COI-5P gene). A total of 417 individuals belonging to 44 species from 10 genera and three families were collected. Five species are newly recorded for Latium. A total of 98 COI-5P sequences were generated and deposited in BOLD and GenBank, including the first COI-5P record for *Nomada nausicaa* Schmiedeknecht, 1882. The integrative approach proved particularly valuable for resolving challenging identifications and for highlighting some taxonomic issues — particularly within the genus *Melecta* — that clearly require further molecular and morphological studies. Our findings provide new distributional data on cleptoparasitic bees in central Italy and expand the availability of COI-5P sequences in public molecular databases.

## Introduction

Bees (Hymenoptera, Apoidea, Anthophila) represent the most important group of insect pollinators, with more than 20,000 described species classified into seven families (Michener 2007, Potts et al. 2010, Ascherl and Pickering 2020), although recent estimates suggest the total number may exceed 26,000 species (Dorey et al. 2026). In the Mediterranean region, six families are present, encompassing a wide range of life-history strategies, including solitary, social and parasitic life-styles, as well as ground-nesting or aerial nesting habits and various degrees of pollen specialization (Michener 2007, Michez et al. 2026). Cleptoparasitic bees — also referred to as cuckoo bees — account for approximately 13% of bee species described globally (Danforth et al. 2019). In the Mediterranean, they belong exclusively to three families: Apidae, Halictidae and Megachilidae. Female cuckoo bees enter the nests of their bee hosts and lay eggs in the provisioned cells, and subsequently their larvae develop by feeding on the stored pollen provisions (Michener 2007). The host range of cuckoo bees varies considerably, from species that are strictly associated with a single host to those that exploit multiple hosts even from different families (Bogusch 2006, Michener 2007). However, generalist cuckoo bees may behave as species-specialist at the individual level (Habermannová et al. 2013).

Cleptoparasitic bees play a regulatory role within bee communities given their ecological role (Combes 1996) and can reflect the diversity of wild bee fauna as demonstrated by Sheffield et al. (2013) across agroecosystem gradients in Canada and, more recently, by Annessi et al. (2026) in urban nature reserves. Indeed, their presence and reproductive success depend on the availability of suitable host species (Finke and Denno 2004). Moreover, cleptoparasitic bees are known to respond rapidly to environmental disturbances (Sheffield et al. 2013, Annessi et al. 2026), a pattern particularly evident in their reduced richness and abundance in urban environments compared with more natural habitats (Banaszak-Cibicka et al. 2018, Ferrari and Polidori 2022, Prendergast et al. 2022).

Despite their sensitivity to environmental disturbances and their role in host regulation, the most recent IUCN European Red List of Bees (Michez et al. 2026) reveals that parasitic bee species suffer from a considerable lack of knowledge in Europe, with several genera displaying notably high proportions of Data Deficient species (e.g., *Metadioxys* with 100% and *Melecta* with 64.0%), partly due to the natural rarity of these bees and the taxonomic uncertainties that still affect many groups. Moreover, some parasitic bee genera also appear to be particularly threatened, with some genera displaying >20% of threatened species (e.g., *Biastes* with 33.3% and *Ammobatoides* with 25%), although the actual extent of this threat remains difficult to assess given current knowledge gaps, which severely compromise a reliable assessment of their conservation status. For this reason, continued monitoring is essential to address those gaps, particularly in regions such as the Mediterranean Basin, where research remains limited despite the region being a global hotspot for bee diversity (Orr et al. 2021). This region is also among the most vulnerable to increasing warming and aridity driven by global change (Malcolm et al. 2006, Klausmeyer and Shaw 2009). Given their ecological role and close dependence on host availability, detailed faunistic data on cleptoparasitic bees may contribute to the assessment of local bee diversity (Sheffield et al. 2013, Annessi et al. 2026).

However, the challenging morphological identification of bee species, due to interspecific similarity, intraspecific morphological variability, and the presence of cryptic or closely related species complexes (e.g., Gibbs 2009, Praz et al. 2022, Annessi et al. 2025a, Flaminio et al. 2025) may hinder the consistent recording of species richness. In this context, molecular tools such as DNA barcoding targeting the mitochondrial Cytochrome C Oxidase Subunit I (COI) are useful to support species identification as well as to enable the assessment of lineage coherence and the detection of divergent or geographically structured lineages, or cryptic diversity (Gibbs 2009, Hebert et al. 2003, Schmidt et al. 2015).

In this work, we provide a faunistic account of cleptoparasitic bees (Apoidea, Anthophila) collected as part of an ecological study conducted in Mediterranean rural protected areas in the province of Rome (Latium, Italy). Specimens were identified using an integrative taxonomic approach combining morphological and molecular evidence, which is known to increase accuracy and the detection of potential cryptic diversity (Dayrat 2005), and also allows the enlargement of the availability of COI-5P sequences for cleptoparasitic bees in public databases (BOLD and GenBank).

## Materials and methods

### Study area and bee sampling

**Figure 1.**
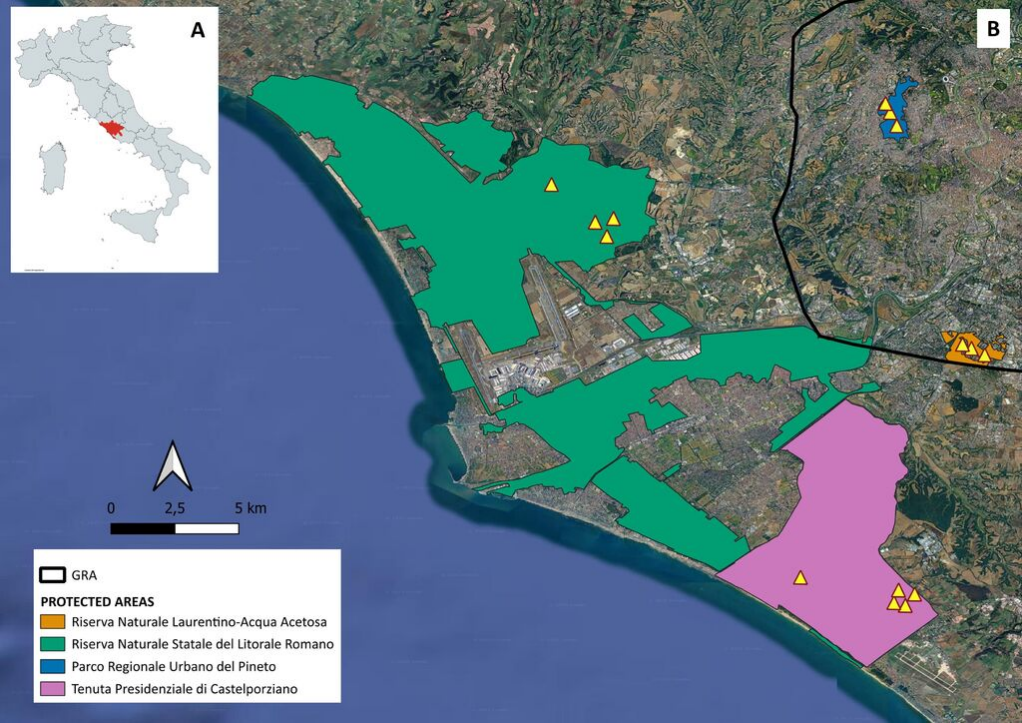
Location of the study area in Italy with the Province of Rome highlighted in red (**A**). Map shows the position of sampling transects (yellow triangles) and surveyed protected areas; GRA (Grande Raccordo Anulare) indicates the motorway ring road delimiting the core urban extent of Rome (**B**). Base map: orthophoto © 2025 Google.

The study was carried out in rural areas within four protected areas in the province of Rome: (1) Parco Regionale Urbano del Pineto (PIN), (2) Riserva Naturale Laurentino-Acqua Acetosa (LAU), (3) Riserva Naturale Statale del Litorale Romano (LIT), and (4) Tenuta Presidenziale di Castelporziano (CAS) (Fig. 1). PIN and LAU are located within the boundaries of the “Grande Raccordo Anulare”, the motorway ring delimiting the urban area of Rome, whereas LIT and CAS are in the Tyrrhenian coastal sector (Fig. 1). The Parco Regionale Urbano del Pineto (247 ha) hosts a remarkable diversity of habitats — including cork oak (*Quercus suber* L., 1753) woodlands, species-rich grasslands, ponds, and wetlands — supporting 642 plant species and representing the richest plant-species density in Rome (Celesti Grapow et al. 1995). The Riserva Naturale Laurentino-Acqua Acetosa is largely dominated by agricultural land use (ca. 75%), with remnant patches of spontaneous vegetation, while nearly 10% of the area is covered by woodland formations (Iamonico and Lorenzetti 2008). The Riserva Naturale Statale del Litorale Romano (ca. 15,900 ha) is characterized by a high geomorphological and pedological heterogeneity, resulting in a mosaic of habitats including evergreen woodlands, riparian corridors and river mouths, coastal dune systems, wetlands, extensive Mediterranean scrublands, as well as agricultural areas and pastures (Blasi 1994, Corona 2001). The Tenuta Presidenziale di Castelporziano (ca. 6,000 ha) represents one of the largest and best-preserved Mediterranean coastal landscapes, predominantly characterized by relict mesophilous and hygrophilous forests, together with a mosaic of natural habitats, planted pine woods, cultivated fields and pastures (Fattorini and Maltzeff 2001).

Cleptoparasitic bees were sampled using entomological nets along fifteen fixed transects of size 200 × 2 m (Quaranta et al. 2004) (Fig. 1), separated by at least 500 meters, and evenly distributed across three different land-use classes according to the CORINE Land Cover classification: "non-irrigated arable land" (2.1.1), "pastures" (2.3.1), and "land principally occupied by agriculture with significant areas of natural vegetation" (2.4.3) (Büttner 2014). Field sampling was conducted from March to September 2025, with monthly surveys at each transect, except in April and May, when sampling was carried out twice per month to account for higher bee activity in spring (e.g., Herrera et al. 2023, Fortini et al. 2024). Each transect was walked once per sampling session by two people, for 50 minutes of regular walking between 9.30 am and 3.00 pm. Sampling was conducted only when the temperature was above 15 °C, the wind was absent or light (<3 m/s), there was no rain, and the vegetation was dry. Each sample collected was assigned to a unique identification code, date, coordinates (WGS84), and Corine Land Cover class. The bee specimens were prepared and stored dry in entomological boxes and preserved in the “Museum of Zoology and Comparative Anatomy” of the Department of Science, Roma Tre University (Rome, Italy).

### DNA barcoding

Total genomic DNA was extracted from one leg following the salting out protocol (Sambrook et al. 1989), eluted in 100 μL H2O milliq, and stored at -20 °C. The 5' end barcode fragment of the mitochondrial Cytochrome C Oxidase Subunit I (COI-5P) gene was amplified using the primer pair LCO1490 (5’-GGTCAACAAATCATAAAGATATTG-3’) and HCO2198 (5’- TAAACTTCAGGTGACCAAAATCA-3’) (Folmer et al. 1994). For each PCR, an aliquot of 1 μL of DNA and 24 μL of reaction mixture (MIX) containing: 3 μl of Buffer 10×, 1 μl MgCl2 50 mM, 0.5 μl dNTPs 10 mM, 0.5 μl primer forward 10 mM, 0.5 μl primer reverse 10 mM, 0.5 μl BSA, 0.2 Taq DNA polymerase 5U/μl and 17.8 H2O milliQ. The PCR cycling conditions were based on Marconi et al. (2022). Three μl of PCR products were used to determine amplification success by agarose (1%) gel run stained with 1 μl of Midori Green Advance (Nippon Genetics Europe). Purification and Sanger sequencing of the amplified products were performed by Macrogen company (Milan, Italy). COI-5P sequences were checked and edited using Geneious Prime software (ver. 2023.1.2) and then deposited in BOLD and GenBank under Acc. n° PZ028938 – PZ029035.

### Species identification

Specimens were first identified morphologically (by authors MA and MM) using dichotomous keys (such as Amiet et al. 2004, Bogusch and Straka 2012, Rasmont 2014, Smit 2018, Wood 2025) and through the consultation of reference collections at the Museum of Zoology of Sapienza University of Rome (Rome, Italy) and the Biodiversitätszentrum Oberösterreich (Linz, Austria). Specimens with uncertain morphological identification were processed for DNA barcoding to confirm their species-level identity (one species of *Epeolus*, five species of *Nomada*, one species of *Sphecodes*, and two species of *Coelioxys*). In addition, three specimens per species with a confident morphological identification were barcoded where possible, to increase the record of species genetic variability in public databases and detect potential cryptic diversity. To confirm the identification of the barcoded specimens, we relied on the BOLD v4 (Ratnasingham et al. 2024) identification engine (available online at https://id.boldsystems.org/), using the Animal Library (Public) database and the Rapid Species Search operating mode. The sequences were then aligned with Muscle (Edgar 2004), implemented on MEGA 7 (Kumar et al. 2016, Tamura et al. 2021). The resulting alignment was used to generate a distance-based Neighbor-Joining (NJ) consensus tree (K2P model, 500 bootstrap replicates) in MEGA 7 to assess potential cross-contamination and correspondence between molecular clusters and morphologically identified species.

For specimens whose morphological identification was uncertain, as well as for cases in which the BOLD identification showed less than 97% sequence similarity or disagreed with the morphological identification, the corresponding sequences were aligned (as described above) together with reference sequences retrieved from BOLD for specimens belonging to the same genus (Suppl. material 1: Table S1-S5). This procedure followed the criteria established by Annessi et al. (2025b). Reference sequences were selected based on BIN assignment (Ratnasingham and Hebert 2013), sequence quality, and country of origin, including only specimens from the Western Palearctic region. For each species, approximately three to five sequences belonging to the same BIN and country were selected, while sequences that were too short (<250 bp) were excluded. The resulting alignments were used to construct consensus Neighbor-Joining (NJ) phylogenetic trees (500 bootstrap replicates, K2P model) to evaluate clustering into monophyletic clades within bee genera. Final species names were assigned by combining morphological and molecular evidence obtained through morphology (MORPHO ID) and BOLD (BOLD ID, % similarity), and, if necessary, considering the relative position of analysed specimens within the comprehensive NJ phylogenetic trees. The classification and the nomenclature used in this paper follow that proposed in the new checklist of wild bees of Europe (Ghisbain et al. 2025), and for some details in the checklist by Comba (2019). Species distribution was referenced from previous literature and from the checklist “Hymenoptera: Apoidea: Anthophila of Italy” by Comba (2019), the “Atlas Hymenoptera” by Rasmont and Haubruge (2015) and the “Global Biodiversity Information Facility” (GBIF 2026).

## Checklists

### Apidae

#### Eupavlovskia obscura

(Friese, 1895)

0348A3AE-C03F-5BD6-90AA-30FEB0C19812

##### Materials

- **Type status:** Other material. **Occurrence:** recordedBy: M. Annessi; individualCount: 4; sex: 4 males; lifeStage: adult; occurrenceID: 1DB44FAF-0EE1-5A17-937A-3AEA7F63AFA9; **Taxon:** class: Insecta; order: Hymenoptera; family: Apidae; genus: Eupavlovskia; specificEpithet: obscura; scientificNameAuthorship: (Friese, 1895); **Location:** country: Italy; countryCode: IT; stateProvince: Roma; locality: Parco Regionale Urbano del Pineto; decimalLatitude: 41.922833; decimalLongitude: 12.426278; geodeticDatum: WGS84; coordinatePrecision: 0.0002; **Identification:** identifiedBy: M. Annessi; **Event:** samplingProtocol: entomological net; eventDate: 3/20/2025; habitat: land principally occupied by agriculture with significant areas of natural vegetation; **Record Level:** institutionID: Roma Tre University; collectionCode: Clepto-01
- **Type status:** Other material. **Occurrence:** recordedBy: M. Annessi; individualCount: 1; sex: 1 female; lifeStage: adult; occurrenceID: 56C2B366-B4D6-5C8D-B18F-09F8CCB4C075; **Taxon:** class: Insecta; order: Hymenoptera; family: Apidae; genus: Eupavlovskia; specificEpithet: obscura; scientificNameAuthorship: (Friese, 1895); **Location:** country: Italy; countryCode: IT; stateProvince: Roma; locality: Parco Regionale Urbano del Pineto; decimalLatitude: 41.912056; decimalLongitude: 12.431611; geodeticDatum: WGS84; coordinatePrecision: 0.0002; **Identification:** identifiedBy: M. Annessi; **Event:** samplingProtocol: entomological net; eventDate: 3/20/2025; habitat: non-irrigated arable land; **Record Level:** institutionID: Roma Tre University; collectionCode: Clepto-01
- **Type status:** Other material. **Occurrence:** recordedBy: M. Annessi; individualCount: 3; sex: 2 males, 1 female; lifeStage: adult; occurrenceID: 6790965B-835B-588D-B092-E162FA2B86AF; **Taxon:** class: Insecta; order: Hymenoptera; family: Apidae; genus: Eupavlovskia; specificEpithet: obscura; scientificNameAuthorship: (Friese, 1895); **Location:** country: Italy; countryCode: IT; stateProvince: Roma; locality: Tenuta Presidenziale di Castelporziano; decimalLatitude: 41.687306; decimalLongitude: 12.430194; geodeticDatum: WGS84; coordinatePrecision: 0.0002; **Identification:** identifiedBy: M. Annessi; **Event:** samplingProtocol: entomological net; eventDate: 3/21/2025; habitat: pastures; **Record Level:** institutionID: Roma Tre University; collectionCode: Clepto-01
- **Type status:** Other material. **Occurrence:** recordedBy: M. Annessi; individualCount: 1; sex: 1 male; lifeStage: adult; occurrenceID: A0FA4D66-A48B-5056-8310-B5F671BC59D7; **Taxon:** class: Insecta; order: Hymenoptera; family: Apidae; genus: Eupavlovskia; specificEpithet: obscura; scientificNameAuthorship: (Friese, 1895); **Location:** country: Italy; countryCode: IT; stateProvince: Roma; locality: Riserva Naturale Statale del Litorale Romano; decimalLatitude: 41.884889; decimalLongitude: 12.269306; geodeticDatum: WGS84; coordinatePrecision: 0.0002; **Identification:** identifiedBy: M. Annessi; **Event:** samplingProtocol: entomological net; eventDate: 3/25/2025; habitat: non-irrigated arable land; **Record Level:** institutionID: Roma Tre University; collectionCode: Clepto-01
- **Type status:** Other material. **Occurrence:** recordedBy: M. Annessi; individualCount: 14; sex: 12 males, 2 females; lifeStage: adult; occurrenceID: 226EB315-CBD8-53E5-B90D-9BFF338D8763; **Taxon:** class: Insecta; order: Hymenoptera; family: Apidae; genus: Eupavlovskia; specificEpithet: obscura; scientificNameAuthorship: (Friese, 1895); **Location:** country: Italy; countryCode: IT; stateProvince: Roma; locality: Riserva Naturale Statale del Litorale Romano; decimalLatitude: 41.868750; decimalLongitude: 12.298028; geodeticDatum: WGS84; coordinatePrecision: 0.0002; **Identification:** identifiedBy: M. Annessi; **Event:** samplingProtocol: entomological net; eventDate: 3/25/2025; habitat: land principally occupied by agriculture with significant areas of natural vegetation; **Record Level:** institutionID: Roma Tre University; collectionCode: Clepto-01
- **Type status:** Other material. **Occurrence:** recordedBy: M. Annessi; individualCount: 1; sex: 1 male; lifeStage: adult; occurrenceID: C8DD118B-0BA4-5E73-8BD1-39B0FDED5E39; **Taxon:** class: Insecta; order: Hymenoptera; family: Apidae; genus: Eupavlovskia; specificEpithet: obscura; scientificNameAuthorship: (Friese, 1895); **Location:** country: Italy; countryCode: IT; stateProvince: Roma; locality: Riserva Naturale Statale del Litorale Romano; decimalLatitude: 41.859389; decimalLongitude: 12.294889; geodeticDatum: WGS84; coordinatePrecision: 0.0002; **Identification:** identifiedBy: M. Annessi; **Event:** samplingProtocol: entomological net; eventDate: 3/25/2025; habitat: land principally occupied by agriculture with significant areas of natural vegetation; **Record Level:** institutionID: Roma Tre University; collectionCode: Clepto-01
- **Type status:** Other material. **Occurrence:** recordedBy: M. Annessi; individualCount: 24; sex: 14 males, 10 females; lifeStage: adult; occurrenceID: 9524FA56-9FC3-545D-8C32-2F65021A64B3; **Taxon:** class: Insecta; order: Hymenoptera; family: Apidae; genus: Eupavlovskia; specificEpithet: obscura; scientificNameAuthorship: (Friese, 1895); **Location:** country: Italy; countryCode: IT; stateProvince: Roma; locality: Riserva Naturale Laurentino-Acqua Acetosa; decimalLatitude: 41.806417; decimalLongitude: 12.467139; geodeticDatum: WGS84; coordinatePrecision: 0.0002; **Identification:** identifiedBy: M. Annessi; **Event:** samplingProtocol: entomological net; eventDate: 3/30/2025; habitat: non-irrigated arable land; **Record Level:** institutionID: Roma Tre University; collectionCode: Clepto-01
- **Type status:** Other material. **Occurrence:** recordedBy: M. Annessi; individualCount: 1; sex: 1 male; lifeStage: adult; occurrenceID: 4C2C01A9-6A86-5FC2-8351-1EB1E404CE63; **Taxon:** class: Insecta; order: Hymenoptera; family: Apidae; genus: Eupavlovskia; specificEpithet: obscura; scientificNameAuthorship: (Friese, 1895); **Location:** country: Italy; countryCode: IT; stateProvince: Roma; locality: Riserva Naturale Laurentino-Acqua Acetosa; decimalLatitude: 41.804083; decimalLongitude: 12.473833; geodeticDatum: WGS84; coordinatePrecision: 0.0002; **Identification:** identifiedBy: M. Annessi; **Event:** samplingProtocol: entomological net; eventDate: 3/30/2025; habitat: non-irrigated arable land; **Record Level:** institutionID: Roma Tre University; collectionCode: Clepto-01
- **Type status:** Other material. **Occurrence:** recordedBy: M. Annessi; individualCount: 23; sex: 5 males, 18 females; lifeStage: adult; occurrenceID: 60E85F9C-2EE2-5934-B604-C7DBA127D596; **Taxon:** class: Insecta; order: Hymenoptera; family: Apidae; genus: Eupavlovskia; specificEpithet: obscura; scientificNameAuthorship: (Friese, 1895); **Location:** country: Italy; countryCode: IT; stateProvince: Roma; locality: Parco Regionale Urbano del Pineto; decimalLatitude: 41.922833; decimalLongitude: 12.426278; geodeticDatum: WGS84; coordinatePrecision: 0.0002; **Identification:** identifiedBy: M. Annessi; **Event:** samplingProtocol: entomological net; eventDate: 4/2/2025; habitat: land principally occupied by agriculture with significant areas of natural vegetation; **Record Level:** institutionID: Roma Tre University; collectionCode: Clepto-01
- **Type status:** Other material. **Occurrence:** recordedBy: M. Annessi; individualCount: 3; sex: 1 male, 2 females; lifeStage: adult; occurrenceID: CB147656-D984-5CF4-9107-2F6D4855252E; **Taxon:** class: Insecta; order: Hymenoptera; family: Apidae; genus: Eupavlovskia; specificEpithet: obscura; scientificNameAuthorship: (Friese, 1895); **Location:** country: Italy; countryCode: IT; stateProvince: Roma; locality: Parco Regionale Urbano del Pineto; decimalLatitude: 41.918417; decimalLongitude: 12.428694; geodeticDatum: WGS84; coordinatePrecision: 0.0002; **Identification:** identifiedBy: M. Annessi; **Event:** samplingProtocol: entomological net; eventDate: 4/2/2025; habitat: land principally occupied by agriculture with significant areas of natural vegetation; **Record Level:** institutionID: Roma Tre University; collectionCode: Clepto-01
- **Type status:** Other material. **Occurrence:** recordedBy: M. Annessi; individualCount: 2; sex: 2 females; lifeStage: adult; occurrenceID: 110EFC4A-D225-55F0-AFBE-AF0BA3B26DBA; **Taxon:** class: Insecta; order: Hymenoptera; family: Apidae; genus: Eupavlovskia; specificEpithet: obscura; scientificNameAuthorship: (Friese, 1895); **Location:** country: Italy; countryCode: IT; stateProvince: Roma; locality: Parco Regionale Urbano del Pineto; decimalLatitude: 41.912056; decimalLongitude: 12.431611; geodeticDatum: WGS84; coordinatePrecision: 0.0002; **Identification:** identifiedBy: M. Annessi; **Event:** samplingProtocol: entomological net; eventDate: 4/2/2025; habitat: land principally occupied by agriculture with significant areas of natural vegetation; **Record Level:** institutionID: Roma Tre University; collectionCode: Clepto-01
- **Type status:** Other material. **Occurrence:** recordedBy: M. Annessi; individualCount: 12; sex: 2 males, 10 females; lifeStage: adult; occurrenceID: A00D3EA4-A654-5B50-8F74-00E831FB73C1; **Taxon:** class: Insecta; order: Hymenoptera; family: Apidae; genus: Eupavlovskia; specificEpithet: obscura; scientificNameAuthorship: (Friese, 1895); **Location:** country: Italy; countryCode: IT; stateProvince: Roma; locality: Riserva Naturale Statale del Litorale Romano; decimalLatitude: 41.868750; decimalLongitude: 12.298028; geodeticDatum: WGS84; coordinatePrecision: 0.0002; **Identification:** identifiedBy: M. Annessi; **Event:** samplingProtocol: entomological net; eventDate: 4/5/2025; habitat: land principally occupied by agriculture with significant areas of natural vegetation; **Record Level:** institutionID: Roma Tre University; collectionCode: Clepto-01
- **Type status:** Other material. **Occurrence:** recordedBy: M. Annessi; individualCount: 20; sex: 2 males, 18 females; lifeStage: adult; occurrenceID: 7387D09D-AD81-565A-8A29-2E52066AB78A; **Taxon:** class: Insecta; order: Hymenoptera; family: Apidae; genus: Eupavlovskia; specificEpithet: obscura; scientificNameAuthorship: (Friese, 1895); **Location:** country: Italy; countryCode: IT; stateProvince: Roma; locality: Riserva Naturale Laurentino-Acqua Acetosa; decimalLatitude: 41.806417; decimalLongitude: 12.467139; geodeticDatum: WGS84; coordinatePrecision: 0.0002; **Identification:** identifiedBy: M. Annessi; **Event:** samplingProtocol: entomological net; eventDate: 4/11/2025; habitat: non-irrigated arable land; **Record Level:** institutionID: Roma Tre University; collectionCode: Clepto-01
- **Type status:** Other material. **Occurrence:** recordedBy: M. Annessi; individualCount: 16; sex: 2 males, 14 females; lifeStage: adult; occurrenceID: C56A9F76-6D41-5C77-9EA5-7D2798F7BF1D; **Taxon:** class: Insecta; order: Hymenoptera; family: Apidae; genus: Eupavlovskia; specificEpithet: obscura; scientificNameAuthorship: (Friese, 1895); **Location:** country: Italy; countryCode: IT; stateProvince: Roma; locality: Parco Regionale Urbano del Pineto; decimalLatitude: 41.922833; decimalLongitude: 12.426278; geodeticDatum: WGS84; coordinatePrecision: 0.0002; **Identification:** identifiedBy: M. Annessi; **Event:** samplingProtocol: entomological net; eventDate: 4/16/2025; habitat: land principally occupied by agriculture with significant areas of natural vegetation; **Record Level:** institutionID: Roma Tre University; collectionCode: Clepto-01
- **Type status:** Other material. **Occurrence:** recordedBy: M. Annessi; individualCount: 4; sex: 4 females; lifeStage: adult; occurrenceID: 6C0F82B1-CC39-50D5-AFBB-984CDA31C647; **Taxon:** class: Insecta; order: Hymenoptera; family: Apidae; genus: Eupavlovskia; specificEpithet: obscura; scientificNameAuthorship: (Friese, 1895); **Location:** country: Italy; countryCode: IT; stateProvince: Roma; locality: Riserva Naturale Statale del Litorale Romano; decimalLatitude: 41.868750; decimalLongitude: 12.298028; geodeticDatum: WGS84; coordinatePrecision: 0.0002; **Identification:** identifiedBy: M. Annessi; **Event:** samplingProtocol: entomological net; eventDate: 4/22/2025; habitat: land principally occupied by agriculture with significant areas of natural vegetation; **Record Level:** institutionID: Roma Tre University; collectionCode: Clepto-01
- **Type status:** Other material. **Occurrence:** recordedBy: M. Annessi; individualCount: 5; sex: 5 females; lifeStage: adult; occurrenceID: 779173BA-9885-57CF-B825-C4211ACD74F8; **Taxon:** class: Insecta; order: Hymenoptera; family: Apidae; genus: Eupavlovskia; specificEpithet: obscura; scientificNameAuthorship: (Friese, 1895); **Location:** country: Italy; countryCode: IT; stateProvince: Roma; locality: Riserva Naturale Laurentino-Acqua Acetosa; decimalLatitude: 41.806417; decimalLongitude: 12.467139; geodeticDatum: WGS84; coordinatePrecision: 0.0002; **Identification:** identifiedBy: M. Annessi; **Event:** samplingProtocol: entomological net; eventDate: 4/26/2025; habitat: non-irrigated arable land; **Record Level:** institutionID: Roma Tre University; collectionCode: Clepto-01
- **Type status:** Other material. **Occurrence:** recordedBy: M. Annessi; individualCount: 6; sex: 6 females; lifeStage: adult; occurrenceID: E8C115C1-22C0-52CA-B219-C678E22F9CFF; **Taxon:** class: Insecta; order: Hymenoptera; family: Apidae; genus: Eupavlovskia; specificEpithet: obscura; scientificNameAuthorship: (Friese, 1895); **Location:** country: Italy; countryCode: IT; stateProvince: Roma; locality: Tenuta Presidenziale di Castelporziano; decimalLatitude: 41.687306; decimalLongitude: 12.430194; geodeticDatum: WGS84; coordinatePrecision: 0.0002; **Identification:** identifiedBy: M. Annessi; **Event:** samplingProtocol: entomological net; eventDate: 4/3/2025; habitat: pastures; **Record Level:** institutionID: Roma Tre University; collectionCode: Clepto-01
- **Type status:** Other material. **Occurrence:** recordedBy: M. Annessi; individualCount: 1; sex: 1 female; lifeStage: adult; occurrenceID: BDA80DD3-1FA9-5B77-96BE-6FCE817829A8; **Taxon:** class: Insecta; order: Hymenoptera; family: Apidae; genus: Eupavlovskia; specificEpithet: obscura; scientificNameAuthorship: (Friese, 1895); **Location:** country: Italy; countryCode: IT; stateProvince: Roma; locality: Tenuta Presidenziale di Castelporziano; decimalLatitude: 41.699500; decimalLongitude: 12.386389; geodeticDatum: WGS84; coordinatePrecision: 0.0002; **Identification:** identifiedBy: M. Annessi; **Event:** samplingProtocol: entomological net; eventDate: 4/3/2025; habitat: pastures; **Record Level:** institutionID: Roma Tre University; collectionCode: Clepto-01

#### Eupavlovskia funeraria

(Smith, 1854)

EDC89D8A-A212-5013-BD7E-122442F3621E

##### Materials

- **Type status:** Other material. **Occurrence:** recordedBy: M. Annessi; individualCount: 1; sex: 1 male; lifeStage: adult; occurrenceID: 2BA7E66C-3A00-535A-9BBD-99E7EE11D6A7; **Taxon:** class: Insecta; order: Hymenoptera; family: Apidae; genus: Eupavlovskia; specificEpithet: funeraria; scientificNameAuthorship: (Smith, 1854); **Location:** country: Italy; countryCode: IT; stateProvince: Roma; locality: Riserva Naturale Laurentino-Acqua Acetosa; decimalLatitude: 41.806417; decimalLongitude: 12.467139; geodeticDatum: WGS84; coordinatePrecision: 0.0002; **Identification:** identifiedBy: M. Annessi; **Event:** samplingProtocol: entomological net; eventDate: 4/26/2025; habitat: non-irrigated arable land; **Record Level:** institutionID: Roma Tre University; collectionCode: Clepto-01
- **Type status:** Other material. **Occurrence:** recordedBy: M. Annessi; individualCount: 3; sex: 3 males; lifeStage: adult; occurrenceID: 3EE97BDE-9D6B-5295-8EF2-01F0BAE7B04A; **Taxon:** class: Insecta; order: Hymenoptera; family: Apidae; genus: Eupavlovskia; specificEpithet: funeraria; scientificNameAuthorship: (Smith, 1854); **Location:** country: Italy; countryCode: IT; stateProvince: Roma; locality: Parco Regionale Urbano del Pineto; decimalLatitude: 41.918417; decimalLongitude: 12.428694; geodeticDatum: WGS84; coordinatePrecision: 0.0002; **Identification:** identifiedBy: M. Annessi; **Event:** samplingProtocol: entomological net; eventDate: 5/7/2025; habitat: land principally occupied by agriculture with significant areas of natural vegetation; **Record Level:** institutionID: Roma Tre University; collectionCode: Clepto-01
- **Type status:** Other material. **Occurrence:** recordedBy: M. Annessi; individualCount: 1; sex: 1 male; lifeStage: adult; occurrenceID: C69B95F4-1A7B-5FAC-B5D4-392E2308814B; **Taxon:** class: Insecta; order: Hymenoptera; family: Apidae; genus: Eupavlovskia; specificEpithet: funeraria; scientificNameAuthorship: (Smith, 1854); **Location:** country: Italy; countryCode: IT; stateProvince: Roma; locality: Parco Regionale Urbano del Pineto; decimalLatitude: 41.912056; decimalLongitude: 12.431611; geodeticDatum: WGS84; coordinatePrecision: 0.0002; **Identification:** identifiedBy: M. Annessi; **Event:** samplingProtocol: entomological net; eventDate: 5/7/2025; habitat: land principally occupied by agriculture with significant areas of natural vegetation; **Record Level:** institutionID: Roma Tre University; collectionCode: Clepto-01

#### Melecta (Melecta) leucorhyncha taormina

Gribodo, 1893

914E55A8-F500-56CF-8603-478FDD8AB198

##### Materials

- **Type status:** Other material. **Occurrence:** recordedBy: M. Annessi; individualCount: 2; sex: 2 females; lifeStage: adult; occurrenceID: 1040432C-E2E8-57A7-88B9-1C0A0334E78E; **Taxon:** class: Insecta; order: Hymenoptera; family: Apidae; genus: Melecta; specificEpithet: leucorhyncha; infraspecificEpithet: taormina; scientificNameAuthorship: Gribodo, 1893; **Location:** country: Italy; countryCode: IT; stateProvince: Roma; locality: Parco Regionale Urbano del Pineto; decimalLatitude: 41.922833; decimalLongitude: 12.426278; geodeticDatum: WGS84; coordinatePrecision: 0.0002; **Identification:** identifiedBy: M. Annessi; **Event:** samplingProtocol: entomological net; eventDate: 3/20/2025; habitat: land principally occupied by agriculture with significant areas of natural vegetation; **Record Level:** institutionID: Roma Tre University; collectionCode: Clepto-01
- **Type status:** Other material. **Occurrence:** recordedBy: M. Annessi; individualCount: 2; sex: 2 males; lifeStage: adult; occurrenceID: 71AF8835-BF2F-50E8-8139-647FD47CAE9D; **Taxon:** class: Insecta; order: Hymenoptera; family: Apidae; genus: Melecta; specificEpithet: leucorhyncha; infraspecificEpithet: taormina; scientificNameAuthorship: Gribodo, 1893; **Location:** country: Italy; countryCode: IT; stateProvince: Roma; locality: Parco Regionale Urbano del Pineto; decimalLatitude: 41.918417; decimalLongitude: 12.428694; geodeticDatum: WGS84; coordinatePrecision: 0.0002; **Identification:** identifiedBy: M. Annessi; **Event:** samplingProtocol: entomological net; eventDate: 3/20/2025; habitat: land principally occupied by agriculture with significant areas of natural vegetation; **Record Level:** institutionID: Roma Tre University; collectionCode: Clepto-01
- **Type status:** Other material. **Occurrence:** recordedBy: M. Annessi; individualCount: 2; sex: 1 male, 1 female; lifeStage: adult; occurrenceID: 3BA12174-E61A-5297-B564-E209C073FDA6; **Taxon:** class: Insecta; order: Hymenoptera; family: Apidae; genus: Melecta; specificEpithet: leucorhyncha; infraspecificEpithet: taormina; scientificNameAuthorship: Gribodo, 1893; **Location:** country: Italy; countryCode: IT; stateProvince: Roma; locality: Riserva Naturale Statale del Litorale Romano; decimalLatitude: 41.868750; decimalLongitude: 12.298028; geodeticDatum: WGS84; coordinatePrecision: 0.0002; **Identification:** identifiedBy: M. Annessi; **Event:** samplingProtocol: entomological net; eventDate: 3/25/2025; habitat: land principally occupied by agriculture with significant areas of natural vegetation; **Record Level:** institutionID: Roma Tre University; collectionCode: Clepto-01
- **Type status:** Other material. **Occurrence:** recordedBy: M. Annessi; individualCount: 1; sex: 1 female; lifeStage: adult; occurrenceID: D7C24912-2374-5E85-ACFE-492F9C15E6C6; **Taxon:** class: Insecta; order: Hymenoptera; family: Apidae; genus: Melecta; specificEpithet: leucorhyncha; infraspecificEpithet: taormina; scientificNameAuthorship: Gribodo, 1893; **Location:** country: Italy; countryCode: IT; stateProvince: Roma; locality: Riserva Naturale Statale del Litorale Romano; decimalLatitude: 41.859389; decimalLongitude: 12.294889; geodeticDatum: WGS84; coordinatePrecision: 0.0002; **Identification:** identifiedBy: M. Annessi; **Event:** samplingProtocol: entomological net; eventDate: 3/25/2025; habitat: land principally occupied by agriculture with significant areas of natural vegetation; **Record Level:** institutionID: Roma Tre University; collectionCode: Clepto-01
- **Type status:** Other material. **Occurrence:** recordedBy: M. Annessi; individualCount: 1; sex: 1 male; lifeStage: adult; occurrenceID: 6F546682-82FC-576F-84FB-D1A7F1EC5058; **Taxon:** class: Insecta; order: Hymenoptera; family: Apidae; genus: Melecta; specificEpithet: leucorhyncha; infraspecificEpithet: taormina; scientificNameAuthorship: Gribodo, 1893; **Location:** country: Italy; countryCode: IT; stateProvince: Roma; locality: Tenuta Presidenziale di Castelporziano; decimalLatitude: 41.699500; decimalLongitude: 12.386389; geodeticDatum: WGS84; coordinatePrecision: 0.0002; **Identification:** identifiedBy: M. Annessi; **Event:** samplingProtocol: entomological net; eventDate: 4/3/2025; habitat: pastures; **Record Level:** institutionID: Roma Tre University; collectionCode: Clepto-01
- **Type status:** Other material. **Occurrence:** recordedBy: M. Annessi; individualCount: 2; sex: 2 females; lifeStage: adult; occurrenceID: 638863F2-D5A0-5996-B4B4-9811BFDDB67A; **Taxon:** class: Insecta; order: Hymenoptera; family: Apidae; genus: Melecta; specificEpithet: leucorhyncha; infraspecificEpithet: taormina; scientificNameAuthorship: Gribodo, 1893; **Location:** country: Italy; countryCode: IT; stateProvince: Roma; locality: Riserva Naturale Statale del Litorale Romano; decimalLatitude: 41.868750; decimalLongitude: 12.298028; geodeticDatum: WGS84; coordinatePrecision: 0.0002; **Identification:** identifiedBy: M. Annessi; **Event:** samplingProtocol: entomological net; eventDate: 4/22/2025; habitat: land principally occupied by agriculture with significant areas of natural vegetation; **Record Level:** institutionID: Roma Tre University; collectionCode: Clepto-01

#### Melecta (Melecta) albifrons

(Forster, 1771)

E460073D-A9ED-51CA-8D89-54BD0573BEFD

##### Materials

- **Type status:** Other material. **Occurrence:** recordedBy: M. Annessi; individualCount: 1; sex: 1 male; lifeStage: adult; occurrenceID: 2F673A94-DEC4-5622-AA33-C2E3ECC1EC15; **Taxon:** class: Insecta; order: Hymenoptera; family: Apidae; genus: Melecta; specificEpithet: albifrons; scientificNameAuthorship: (Forster, 1771); **Location:** country: Italy; countryCode: IT; stateProvince: Roma; locality: Riserva Naturale Statale del Litorale Romano; decimalLatitude: 41.859389; decimalLongitude: 12.294889; geodeticDatum: WGS84; coordinatePrecision: 0.0002; **Identification:** identifiedBy: M. Annessi, M. Mei; **Event:** samplingProtocol: entomological net; eventDate: 3/25/2025; habitat: land principally occupied by agriculture with significant areas of natural vegetation; **Record Level:** institutionID: Roma Tre University; collectionCode: Clepto-01
- **Type status:** Other material. **Occurrence:** recordedBy: M. Annessi; individualCount: 1; sex: 1 male; lifeStage: adult; occurrenceID: C782C844-AB34-59AC-8FB7-F8F308FD1DDA; **Taxon:** class: Insecta; order: Hymenoptera; family: Apidae; genus: Melecta; specificEpithet: albifrons; scientificNameAuthorship: (Forster, 1771); **Location:** country: Italy; countryCode: IT; stateProvince: Roma; locality: Tenuta Presidenziale di Castelporziano; decimalLatitude: 41.687306; decimalLongitude: 12.430194; geodeticDatum: WGS84; coordinatePrecision: 0.0002; **Identification:** identifiedBy: M. Annessi, M. Mei; **Event:** samplingProtocol: entomological net; eventDate: 4/3/2025; habitat: pastures; **Record Level:** institutionID: Roma Tre University; collectionCode: Clepto-01
- **Type status:** Other material. **Occurrence:** recordedBy: M. Annessi; individualCount: 1; sex: 1 male; lifeStage: adult; occurrenceID: F99F7930-14C7-59BD-AC26-E02A2BFE92F2; **Taxon:** class: Insecta; order: Hymenoptera; family: Apidae; genus: Melecta; specificEpithet: albifrons; scientificNameAuthorship: (Forster, 1771); **Location:** country: Italy; countryCode: IT; stateProvince: Roma; locality: Riserva Naturale Laurentino-Acqua Acetosa; decimalLatitude: 41.806417; decimalLongitude: 12.467139; geodeticDatum: WGS84; coordinatePrecision: 0.0002; **Identification:** identifiedBy: M. Annessi, M. Mei; **Event:** samplingProtocol: entomological net; eventDate: 4/11/2025; habitat: non-irrigated arable land; **Record Level:** institutionID: Roma Tre University; collectionCode: Clepto-01
- **Type status:** Other material. **Occurrence:** recordedBy: M. Annessi; individualCount: 1; sex: 1 male; lifeStage: adult; occurrenceID: E7A02E4E-B455-5F15-88EB-320BC72F329D; **Taxon:** class: Insecta; order: Hymenoptera; family: Apidae; genus: Melecta; specificEpithet: albifrons; scientificNameAuthorship: (Forster, 1771); **Location:** country: Italy; countryCode: IT; stateProvince: Roma; locality: Riserva Naturale Laurentino-Acqua Acetosa; decimalLatitude: 41.806417; decimalLongitude: 12.467139; geodeticDatum: WGS84; coordinatePrecision: 0.0002; **Identification:** identifiedBy: M. Annessi, M. Mei; **Event:** samplingProtocol: entomological net; eventDate: 4/26/2025; habitat: non-irrigated arable land; **Record Level:** institutionID: Roma Tre University; collectionCode: Clepto-01
- **Type status:** Other material. **Occurrence:** recordedBy: M. Annessi; individualCount: 1; sex: 1 female; lifeStage: adult; occurrenceID: AEA4C2CF-07C6-5EA6-A9CB-940EBC3AD197; **Taxon:** class: Insecta; order: Hymenoptera; family: Apidae; genus: Melecta; specificEpithet: albifrons; scientificNameAuthorship: (Forster, 1771); **Location:** country: Italy; countryCode: IT; stateProvince: Roma; locality: Riserva Naturale Statale del Litorale Romano; decimalLatitude: 41.868750; decimalLongitude: 12.298028; geodeticDatum: WGS84; coordinatePrecision: 0.0002; **Identification:** identifiedBy: M. Annessi, M. Mei; **Event:** samplingProtocol: entomological net; eventDate: 5/26/2025; habitat: land principally occupied by agriculture with significant areas of natural vegetation; **Record Level:** institutionID: Roma Tre University; collectionCode: Clepto-01

#### Melecta (Melecta) aegyptiaca

Radoszkowski, 1876

A2F62F07-E97E-54FD-8C7B-CDB6A79E0FD7

##### Materials

- **Type status:** Other material. **Occurrence:** recordedBy: M. Annessi; individualCount: 2; sex: 2 males; lifeStage: adult; occurrenceID: 527222FA-1B56-5E4D-A56E-D87549F68F92; **Taxon:** class: Insecta; order: Hymenoptera; family: Apidae; genus: Melecta; specificEpithet: aegyptiaca; scientificNameAuthorship: Radoszkowski, 1876; **Location:** country: Italy; countryCode: IT; stateProvince: Roma; locality: Riserva Naturale Laurentino-Acqua Acetosa; decimalLatitude: 41.804083; decimalLongitude: 12.473833; geodeticDatum: WGS84; coordinatePrecision: 0.0002; **Identification:** identifiedBy: M. Annessi; **Event:** samplingProtocol: entomological net; eventDate: 4/26/2025; habitat: non-irrigated arable land; **Record Level:** institutionID: Roma Tre University; collectionCode: Clepto-01

#### Nomada (Gestamen) carnifex

Mocsáry, 1883

B04B9553-C4D1-5ABF-B540-E987D9C41C53

##### Materials

- **Type status:** Other material. **Occurrence:** recordedBy: M. Annessi; individualCount: 1; sex: 1 female; lifeStage: adult; occurrenceID: BE7577F8-EE95-5FA6-A33F-27022C17A511; **Taxon:** class: Insecta; order: Hymenoptera; family: Apidae; genus: Nomada; specificEpithet: carnifex; scientificNameAuthorship: Mocsáry, 1883; **Location:** country: Italy; countryCode: IT; stateProvince: Roma; locality: Parco Regionale Urbano del Pineto; decimalLatitude: 41.918417; decimalLongitude: 12.428694; geodeticDatum: WGS84; coordinatePrecision: 0.0002; **Identification:** identifiedBy: M. Annessi; **Event:** samplingProtocol: entomological net; eventDate: 3/20/2025; habitat: land principally occupied by agriculture with significant areas of natural vegetation; **Record Level:** institutionID: Roma Tre University; collectionCode: Clepto-01
- **Type status:** Other material. **Occurrence:** recordedBy: M. Annessi; individualCount: 8; sex: 8 females; lifeStage: adult; occurrenceID: 2379C3F8-0674-5AA7-9818-02DC217055B2; **Taxon:** class: Insecta; order: Hymenoptera; family: Apidae; genus: Nomada; specificEpithet: carnifex; scientificNameAuthorship: Mocsáry, 1883; **Location:** country: Italy; countryCode: IT; stateProvince: Roma; locality: Riserva Naturale Statale del Litorale Romano; decimalLatitude: 41.884889; decimalLongitude: 12.269306; geodeticDatum: WGS84; coordinatePrecision: 0.0002; **Identification:** identifiedBy: M. Annessi; **Event:** samplingProtocol: entomological net; eventDate: 3/25/2025; habitat: non-irrigated arable land; **Record Level:** institutionID: Roma Tre University; collectionCode: Clepto-01
- **Type status:** Other material. **Occurrence:** recordedBy: M. Annessi; individualCount: 5; sex: 5 females; lifeStage: adult; occurrenceID: 28088672-55D4-574A-A784-5A8717A031D6; **Taxon:** class: Insecta; order: Hymenoptera; family: Apidae; genus: Nomada; specificEpithet: carnifex; scientificNameAuthorship: Mocsáry, 1883; **Location:** country: Italy; countryCode: IT; stateProvince: Roma; locality: Riserva Naturale Laurentino-Acqua Acetosa; decimalLatitude: 41.806417; decimalLongitude: 12.467139; geodeticDatum: WGS84; coordinatePrecision: 0.0002; **Identification:** identifiedBy: M. Annessi; **Event:** samplingProtocol: entomological net; eventDate: 3/30/2025; habitat: non-irrigated arable land; **Record Level:** institutionID: Roma Tre University; collectionCode: Clepto-01
- **Type status:** Other material. **Occurrence:** recordedBy: M. Annessi; individualCount: 3; sex: 2 males, 1 female; lifeStage: adult; occurrenceID: 3C142156-6E01-59D9-96D7-506D39674B88; **Taxon:** class: Insecta; order: Hymenoptera; family: Apidae; genus: Nomada; specificEpithet: carnifex; scientificNameAuthorship: Mocsáry, 1883; **Location:** country: Italy; countryCode: IT; stateProvince: Roma; locality: Riserva Naturale Laurentino-Acqua Acetosa; decimalLatitude: 41.806583; decimalLongitude: 12.461083; geodeticDatum: WGS84; coordinatePrecision: 0.0002; **Identification:** identifiedBy: M. Annessi; **Event:** samplingProtocol: entomological net; eventDate: 3/30/2025; habitat: non-irrigated arable land; **Record Level:** institutionID: Roma Tre University; collectionCode: Clepto-01
- **Type status:** Other material. **Occurrence:** recordedBy: M. Annessi; individualCount: 12; sex: 12 females; lifeStage: adult; occurrenceID: FD6141B4-C3F5-594A-8272-B68311C389A3; **Taxon:** class: Insecta; order: Hymenoptera; family: Apidae; genus: Nomada; specificEpithet: carnifex; scientificNameAuthorship: Mocsáry, 1883; **Location:** country: Italy; countryCode: IT; stateProvince: Roma; locality: Riserva Naturale Statale del Litorale Romano; decimalLatitude: 41.884889; decimalLongitude: 12.269306; geodeticDatum: WGS84; coordinatePrecision: 0.0002; **Identification:** identifiedBy: M. Annessi; **Event:** samplingProtocol: entomological net; eventDate: 4/5/2025; habitat: non-irrigated arable land; **Record Level:** institutionID: Roma Tre University; collectionCode: Clepto-01
- **Type status:** Other material. **Occurrence:** recordedBy: M. Annessi; individualCount: 3; sex: 3 females; lifeStage: adult; occurrenceID: 88053A1C-B690-548A-B9E9-2A3F4B992A61; **Taxon:** class: Insecta; order: Hymenoptera; family: Apidae; genus: Nomada; specificEpithet: carnifex; scientificNameAuthorship: Mocsáry, 1883; **Location:** country: Italy; countryCode: IT; stateProvince: Roma; locality: Riserva Naturale Laurentino-Acqua Acetosa; decimalLatitude: 41.806417; decimalLongitude: 12.467139; geodeticDatum: WGS84; coordinatePrecision: 0.0002; **Identification:** identifiedBy: M. Annessi; **Event:** samplingProtocol: entomological net; eventDate: 4/11/2025; habitat: non-irrigated arable land; **Record Level:** institutionID: Roma Tre University; collectionCode: Clepto-01
- **Type status:** Other material. **Occurrence:** recordedBy: M. Annessi; individualCount: 4; sex: 4 females; lifeStage: adult; occurrenceID: E42DFD8B-30A2-5A93-9180-D7F966D02B07; **Taxon:** class: Insecta; order: Hymenoptera; family: Apidae; genus: Nomada; specificEpithet: carnifex; scientificNameAuthorship: Mocsáry, 1883; **Location:** country: Italy; countryCode: IT; stateProvince: Roma; locality: Riserva Naturale Statale del Litorale Romano; decimalLatitude: 41.884889; decimalLongitude: 12.269306; geodeticDatum: WGS84; coordinatePrecision: 0.0002; **Identification:** identifiedBy: M. Annessi; **Event:** samplingProtocol: entomological net; eventDate: 4/22/2025; habitat: non-irrigated arable land; **Record Level:** institutionID: Roma Tre University; collectionCode: Clepto-01
- **Type status:** Other material. **Occurrence:** recordedBy: M. Annessi; individualCount: 3; sex: 3 females; lifeStage: adult; occurrenceID: E1D798F9-B8B5-544B-8BFB-97872999D9C4; **Taxon:** class: Insecta; order: Hymenoptera; family: Apidae; genus: Nomada; specificEpithet: carnifex; scientificNameAuthorship: Mocsáry, 1883; **Location:** country: Italy; countryCode: IT; stateProvince: Roma; locality: Riserva Naturale Laurentino-Acqua Acetosa; decimalLatitude: 41.806417; decimalLongitude: 12.467139; geodeticDatum: WGS84; coordinatePrecision: 0.0002; **Identification:** identifiedBy: M. Annessi; **Event:** samplingProtocol: entomological net; eventDate: 4/26/2025; habitat: non-irrigated arable land; **Record Level:** institutionID: Roma Tre University; collectionCode: Clepto-01

#### Nomada (Collicula) integra

Brullé, 1833

39657BA6-33F5-5F71-9AF6-52D63ABFE416

##### Materials

- **Type status:** Other material. **Occurrence:** recordedBy: M. Annessi; individualCount: 1; sex: 1 male; lifeStage: adult; occurrenceID: F55CEFAD-9C18-5AB5-BFF0-FAC9CC59A4C9; **Taxon:** class: Insecta; order: Hymenoptera; family: Apidae; genus: Nomada; specificEpithet: integra; scientificNameAuthorship: Brullé, 1833; **Location:** country: Italy; countryCode: IT; stateProvince: Roma; locality: Parco Regionale Urbano del Pineto; decimalLatitude: 41.918417; decimalLongitude: 12.428694; geodeticDatum: WGS84; coordinatePrecision: 0.0002; **Identification:** identifiedBy: M. Annessi; **Event:** samplingProtocol: entomological net; eventDate: 3/20/2025; habitat: land principally occupied by agriculture with significant areas of natural vegetation; **Record Level:** institutionID: Roma Tre University; collectionCode: Clepto-01
- **Type status:** Other material. **Occurrence:** recordedBy: M. Annessi; individualCount: 8; sex: 6 males, 2 females; lifeStage: adult; occurrenceID: 06D217CA-A886-59CB-94EB-8BEF729F3DDF; **Taxon:** class: Insecta; order: Hymenoptera; family: Apidae; genus: Nomada; specificEpithet: integra; scientificNameAuthorship: Brullé, 1833; **Location:** country: Italy; countryCode: IT; stateProvince: Roma; locality: Riserva Naturale Laurentino-Acqua Acetosa; decimalLatitude: 41.806583; decimalLongitude: 12.461083; geodeticDatum: WGS84; coordinatePrecision: 0.0002; **Identification:** identifiedBy: M. Annessi; **Event:** samplingProtocol: entomological net; eventDate: 3/30/2025; habitat: non-irrigated arable land; **Record Level:** institutionID: Roma Tre University; collectionCode: Clepto-01
- **Type status:** Other material. **Occurrence:** recordedBy: M. Annessi; individualCount: 2; sex: 2 females; lifeStage: adult; occurrenceID: 42F48A93-AC47-5FC7-8236-F193920B9400; **Taxon:** class: Insecta; order: Hymenoptera; family: Apidae; genus: Nomada; specificEpithet: integra; scientificNameAuthorship: Brullé, 1833; **Location:** country: Italy; countryCode: IT; stateProvince: Roma; locality: Parco Regionale Urbano del Pineto; decimalLatitude: 41.918417; decimalLongitude: 12.428694; geodeticDatum: WGS84; coordinatePrecision: 0.0002; **Identification:** identifiedBy: M. Annessi; **Event:** samplingProtocol: entomological net; eventDate: 4/2/2025; habitat: land principally occupied by agriculture with significant areas of natural vegetation; **Record Level:** institutionID: Roma Tre University; collectionCode: Clepto-01
- **Type status:** Other material. **Occurrence:** recordedBy: M. Annessi; individualCount: 1; sex: 1 female; lifeStage: adult; occurrenceID: 05347CE0-092A-57DB-B5C2-ED5DC53E54F0; **Taxon:** class: Insecta; order: Hymenoptera; family: Apidae; genus: Nomada; specificEpithet: integra; scientificNameAuthorship: Brullé, 1833; **Location:** country: Italy; countryCode: IT; stateProvince: Roma; locality: Riserva Naturale Laurentino-Acqua Acetosa; decimalLatitude: 41.806417; decimalLongitude: 12.467139; geodeticDatum: WGS84; coordinatePrecision: 0.0002; **Identification:** identifiedBy: M. Annessi; **Event:** samplingProtocol: entomological net; eventDate: 4/11/2025; habitat: non-irrigated arable land; **Record Level:** institutionID: Roma Tre University; collectionCode: Clepto-01
- **Type status:** Other material. **Occurrence:** recordedBy: M. Annessi; individualCount: 1; sex: 1 female; lifeStage: adult; occurrenceID: 2BD98076-03DD-5FD9-91E8-460626521625; **Taxon:** class: Insecta; order: Hymenoptera; family: Apidae; genus: Nomada; specificEpithet: integra; scientificNameAuthorship: Brullé, 1833; **Location:** country: Italy; countryCode: IT; stateProvince: Roma; locality: Riserva Naturale Laurentino-Acqua Acetosa; decimalLatitude: 41.804083; decimalLongitude: 12.473833; geodeticDatum: WGS84; coordinatePrecision: 0.0002; **Identification:** identifiedBy: M. Annessi; **Event:** samplingProtocol: entomological net; eventDate: 4/11/2025; habitat: non-irrigated arable land; **Record Level:** institutionID: Roma Tre University; collectionCode: Clepto-01
- **Type status:** Other material. **Occurrence:** recordedBy: M. Annessi; individualCount: 1; sex: 1 female; lifeStage: adult; occurrenceID: 973B7493-9495-54BC-9120-339B591CEB21; **Taxon:** class: Insecta; order: Hymenoptera; family: Apidae; genus: Nomada; specificEpithet: integra; scientificNameAuthorship: Brullé, 1833; **Location:** country: Italy; countryCode: IT; stateProvince: Roma; locality: Riserva Naturale Laurentino-Acqua Acetosa; decimalLatitude: 41.806583; decimalLongitude: 12.461083; geodeticDatum: WGS84; coordinatePrecision: 0.0002; **Identification:** identifiedBy: M. Annessi; **Event:** samplingProtocol: entomological net; eventDate: 4/11/2025; habitat: non-irrigated arable land; **Record Level:** institutionID: Roma Tre University; collectionCode: Clepto-01
- **Type status:** Other material. **Occurrence:** recordedBy: M. Annessi; individualCount: 5; sex: 3 males, 2 females; lifeStage: adult; occurrenceID: A82D2156-FC75-5B79-859C-711675215322; **Taxon:** class: Insecta; order: Hymenoptera; family: Apidae; genus: Nomada; specificEpithet: integra; scientificNameAuthorship: Brullé, 1833; **Location:** country: Italy; countryCode: IT; stateProvince: Roma; locality: Parco Regionale Urbano del Pineto; decimalLatitude: 41.918417; decimalLongitude: 12.428694; geodeticDatum: WGS84; coordinatePrecision: 0.0002; **Identification:** identifiedBy: M. Annessi; **Event:** samplingProtocol: entomological net; eventDate: 4/16/2025; habitat: land principally occupied by agriculture with significant areas of natural vegetation; **Record Level:** institutionID: Roma Tre University; collectionCode: Clepto-01
- **Type status:** Other material. **Occurrence:** recordedBy: M. Annessi; individualCount: 1; sex: 1 male; lifeStage: adult; occurrenceID: FCCE1263-3112-5459-8D28-39C54B334B42; **Taxon:** class: Insecta; order: Hymenoptera; family: Apidae; genus: Nomada; specificEpithet: integra; scientificNameAuthorship: Brullé, 1833; **Location:** country: Italy; countryCode: IT; stateProvince: Roma; locality: Parco Regionale Urbano del Pineto; decimalLatitude: 41.912056; decimalLongitude: 12.431611; geodeticDatum: WGS84; coordinatePrecision: 0.0002; **Identification:** identifiedBy: M. Annessi; **Event:** samplingProtocol: entomological net; eventDate: 4/16/2025; habitat: land principally occupied by agriculture with significant areas of natural vegetation; **Record Level:** institutionID: Roma Tre University; collectionCode: Clepto-01
- **Type status:** Other material. **Occurrence:** recordedBy: M. Annessi; individualCount: 2; sex: 1 male, 1 female; lifeStage: adult; occurrenceID: 354E615F-0009-55AD-A1B3-DACBEAFA645C; **Taxon:** class: Insecta; order: Hymenoptera; family: Apidae; genus: Nomada; specificEpithet: integra; scientificNameAuthorship: Brullé, 1833; **Location:** country: Italy; countryCode: IT; stateProvince: Roma; locality: Riserva Naturale Laurentino-Acqua Acetosa; decimalLatitude: 41.806417; decimalLongitude: 12.467139; geodeticDatum: WGS84; coordinatePrecision: 0.0002; **Identification:** identifiedBy: M. Annessi; **Event:** samplingProtocol: entomological net; eventDate: 4/26/2025; habitat: non-irrigated arable land; **Record Level:** institutionID: Roma Tre University; collectionCode: Clepto-01
- **Type status:** Other material. **Occurrence:** recordedBy: M. Annessi; individualCount: 1; sex: 1 female; lifeStage: adult; occurrenceID: 666B892A-B348-5F0E-9B42-5DD6ED8A53E9; **Taxon:** class: Insecta; order: Hymenoptera; family: Apidae; genus: Nomada; specificEpithet: integra; scientificNameAuthorship: Brullé, 1833; **Location:** country: Italy; countryCode: IT; stateProvince: Roma; locality: Riserva Naturale Laurentino-Acqua Acetosa; decimalLatitude: 41.806583; decimalLongitude: 12.461083; geodeticDatum: WGS84; coordinatePrecision: 0.0002; **Identification:** identifiedBy: M. Annessi; **Event:** samplingProtocol: entomological net; eventDate: 4/26/2025; habitat: non-irrigated arable land; **Record Level:** institutionID: Roma Tre University; collectionCode: Clepto-01
- **Type status:** Other material. **Occurrence:** recordedBy: M. Annessi; individualCount: 1; sex: 1 male; lifeStage: adult; occurrenceID: EBDEF5F7-248F-5AF7-8D45-52E2556011E6; **Taxon:** class: Insecta; order: Hymenoptera; family: Apidae; genus: Nomada; specificEpithet: integra; scientificNameAuthorship: Brullé, 1833; **Location:** country: Italy; countryCode: IT; stateProvince: Roma; locality: Parco Regionale Urbano del Pineto; decimalLatitude: 41.912056; decimalLongitude: 12.431611; geodeticDatum: WGS84; coordinatePrecision: 0.0002; **Identification:** identifiedBy: M. Annessi; **Event:** samplingProtocol: entomological net; eventDate: 5/7/2025; habitat: land principally occupied by agriculture with significant areas of natural vegetation; **Record Level:** institutionID: Roma Tre University; collectionCode: Clepto-01

#### Nomada (Gestamen) femoralis

Morawitz, 1868

75707A50-855A-57B2-A5A8-C462DF510C9C

##### Materials

- **Type status:** Other material. **Occurrence:** recordedBy: M. Annessi; individualCount: 3; sex: 3 males; lifeStage: adult; occurrenceID: 407815B5-ED92-517A-BA79-5BB3127A070B; **Taxon:** class: Insecta; order: Hymenoptera; family: Apidae; genus: Nomada; specificEpithet: femoralis; scientificNameAuthorship: Morawitz, 1868; **Location:** country: Italy; countryCode: IT; stateProvince: Roma; locality: Riserva Naturale Laurentino-Acqua Acetosa; decimalLatitude: 41.806417; decimalLongitude: 12.467139; geodeticDatum: WGS84; coordinatePrecision: 0.0002; **Identification:** identifiedBy: M. Annessi; **Event:** samplingProtocol: entomological net; eventDate: 3/30/2025; habitat: non-irrigated arable land; **Record Level:** institutionID: Roma Tre University; collectionCode: Clepto-01
- **Type status:** Other material. **Occurrence:** recordedBy: M. Annessi; individualCount: 2; sex: 2 males; lifeStage: adult; occurrenceID: B8E48A1D-45CF-5AAC-8AEE-8B31E0F6B3C4; **Taxon:** class: Insecta; order: Hymenoptera; family: Apidae; genus: Nomada; specificEpithet: femoralis; scientificNameAuthorship: Morawitz, 1868; **Location:** country: Italy; countryCode: IT; stateProvince: Roma; locality: Riserva Naturale Laurentino-Acqua Acetosa; decimalLatitude: 41.804083; decimalLongitude: 12.473833; geodeticDatum: WGS84; coordinatePrecision: 0.0002; **Identification:** identifiedBy: M. Annessi; **Event:** samplingProtocol: entomological net; eventDate: 3/30/2025; habitat: non-irrigated arable land; **Record Level:** institutionID: Roma Tre University; collectionCode: Clepto-01
- **Type status:** Other material. **Occurrence:** recordedBy: M. Annessi; individualCount: 8; sex: 7 males, 1 female; lifeStage: adult; occurrenceID: 22B719E6-ED0A-59AD-BE9F-EA3D76775CED; **Taxon:** class: Insecta; order: Hymenoptera; family: Apidae; genus: Nomada; specificEpithet: femoralis; scientificNameAuthorship: Morawitz, 1868; **Location:** country: Italy; countryCode: IT; stateProvince: Roma; locality: Parco Regionale Urbano del Pineto; decimalLatitude: 41.918417; decimalLongitude: 12.428694; geodeticDatum: WGS84; coordinatePrecision: 0.0002; **Identification:** identifiedBy: M. Annessi; **Event:** samplingProtocol: entomological net; eventDate: 4/2/2025; habitat: land principally occupied by agriculture with significant areas of natural vegetation; **Record Level:** institutionID: Roma Tre University; collectionCode: Clepto-01
- **Type status:** Other material. **Occurrence:** recordedBy: M. Annessi; individualCount: 10; sex: 10 males; lifeStage: adult; occurrenceID: 690962F9-8FE0-505D-AD21-0C33A514FFB5; **Taxon:** class: Insecta; order: Hymenoptera; family: Apidae; genus: Nomada; specificEpithet: femoralis; scientificNameAuthorship: Morawitz, 1868; **Location:** country: Italy; countryCode: IT; stateProvince: Roma; locality: Parco Regionale Urbano del Pineto; decimalLatitude: 41.912056; decimalLongitude: 12.431611; geodeticDatum: WGS84; coordinatePrecision: 0.0002; **Identification:** identifiedBy: M. Annessi; **Event:** samplingProtocol: entomological net; eventDate: 4/2/2025; habitat: land principally occupied by agriculture with significant areas of natural vegetation; **Record Level:** institutionID: Roma Tre University; collectionCode: Clepto-01
- **Type status:** Other material. **Occurrence:** recordedBy: M. Annessi; individualCount: 1; sex: 1 male; lifeStage: adult; occurrenceID: E5605DF3-CB61-5757-A3E3-D2375405ACB0; **Taxon:** class: Insecta; order: Hymenoptera; family: Apidae; genus: Nomada; specificEpithet: femoralis; scientificNameAuthorship: Morawitz, 1868; **Location:** country: Italy; countryCode: IT; stateProvince: Roma; locality: Riserva Naturale Statale del Litorale Romano; decimalLatitude: 41.859389; decimalLongitude: 12.294889; geodeticDatum: WGS84; coordinatePrecision: 0.0002; **Identification:** identifiedBy: M. Annessi; **Event:** samplingProtocol: entomological net; eventDate: 4/5/2025; habitat: land principally occupied by agriculture with significant areas of natural vegetation; **Record Level:** institutionID: Roma Tre University; collectionCode: Clepto-01
- **Type status:** Other material. **Occurrence:** recordedBy: M. Annessi; individualCount: 3; sex: 3 males; lifeStage: adult; occurrenceID: CED0A263-97A2-5404-9927-BE4B095B608C; **Taxon:** class: Insecta; order: Hymenoptera; family: Apidae; genus: Nomada; specificEpithet: femoralis; scientificNameAuthorship: Morawitz, 1868; **Location:** country: Italy; countryCode: IT; stateProvince: Roma; locality: Riserva Naturale Laurentino-Acqua Acetosa; decimalLatitude: 41.806417; decimalLongitude: 12.467139; geodeticDatum: WGS84; coordinatePrecision: 0.0002; **Identification:** identifiedBy: M. Annessi; **Event:** samplingProtocol: entomological net; eventDate: 4/11/2025; habitat: non-irrigated arable land; **Record Level:** institutionID: Roma Tre University; collectionCode: Clepto-01
- **Type status:** Other material. **Occurrence:** recordedBy: M. Annessi; individualCount: 7; sex: 4 males, 3 females; lifeStage: adult; occurrenceID: 43B1B0E7-F7F5-53EE-8600-03E32D7BDB53; **Taxon:** class: Insecta; order: Hymenoptera; family: Apidae; genus: Nomada; specificEpithet: femoralis; scientificNameAuthorship: Morawitz, 1868; **Location:** country: Italy; countryCode: IT; stateProvince: Roma; locality: Parco Regionale Urbano del Pineto; decimalLatitude: 41.918417; decimalLongitude: 12.428694; geodeticDatum: WGS84; coordinatePrecision: 0.0002; **Identification:** identifiedBy: M. Annessi; **Event:** samplingProtocol: entomological net; eventDate: 4/16/2025; habitat: land principally occupied by agriculture with significant areas of natural vegetation; **Record Level:** institutionID: Roma Tre University; collectionCode: Clepto-01
- **Type status:** Other material. **Occurrence:** recordedBy: M. Annessi; individualCount: 2; sex: 2 males; lifeStage: adult; occurrenceID: 7E1A2CFF-64B0-523E-AE56-40C053F1981B; **Taxon:** class: Insecta; order: Hymenoptera; family: Apidae; genus: Nomada; specificEpithet: femoralis; scientificNameAuthorship: Morawitz, 1868; **Location:** country: Italy; countryCode: IT; stateProvince: Roma; locality: Parco Regionale Urbano del Pineto; decimalLatitude: 41.912056; decimalLongitude: 12.431611; geodeticDatum: WGS84; coordinatePrecision: 0.0002; **Identification:** identifiedBy: M. Annessi; **Event:** samplingProtocol: entomological net; eventDate: 4/16/2025; habitat: land principally occupied by agriculture with significant areas of natural vegetation; **Record Level:** institutionID: Roma Tre University; collectionCode: Clepto-01
- **Type status:** Other material. **Occurrence:** recordedBy: M. Annessi; individualCount: 1; sex: 1 female; lifeStage: adult; occurrenceID: F55C26B3-8C6D-547E-A042-04686AD2D81D; **Taxon:** class: Insecta; order: Hymenoptera; family: Apidae; genus: Nomada; specificEpithet: femoralis; scientificNameAuthorship: Morawitz, 1868; **Location:** country: Italy; countryCode: IT; stateProvince: Roma; locality: Riserva Naturale Statale del Litorale Romano; decimalLatitude: 41.884889; decimalLongitude: 12.269306; geodeticDatum: WGS84; coordinatePrecision: 0.0002; **Identification:** identifiedBy: M. Annessi; **Event:** samplingProtocol: entomological net; eventDate: 4/22/2025; habitat: non-irrigated arable land; **Record Level:** institutionID: Roma Tre University; collectionCode: Clepto-01
- **Type status:** Other material. **Occurrence:** recordedBy: M. Annessi; individualCount: 4; sex: 2 males, 2 females; lifeStage: adult; occurrenceID: E820A392-6F43-5820-91DF-E26653D90881; **Taxon:** class: Insecta; order: Hymenoptera; family: Apidae; genus: Nomada; specificEpithet: femoralis; scientificNameAuthorship: Morawitz, 1868; **Location:** country: Italy; countryCode: IT; stateProvince: Roma; locality: Riserva Naturale Statale del Litorale Romano; decimalLatitude: 41.866611; decimalLongitude: 12.289917; geodeticDatum: WGS84; coordinatePrecision: 0.0002; **Identification:** identifiedBy: M. Annessi; **Event:** samplingProtocol: entomological net; eventDate: 4/22/2025; habitat: land principally occupied by agriculture with significant areas of natural vegetation; **Record Level:** institutionID: Roma Tre University; collectionCode: Clepto-01
- **Type status:** Other material. **Occurrence:** recordedBy: M. Annessi; individualCount: 1; sex: 1 female; lifeStage: adult; occurrenceID: 4AD8A8FB-75E4-5030-9B07-8AE5804EAA5B; **Taxon:** class: Insecta; order: Hymenoptera; family: Apidae; genus: Nomada; specificEpithet: femoralis; scientificNameAuthorship: Morawitz, 1868; **Location:** country: Italy; countryCode: IT; stateProvince: Roma; locality: Riserva Naturale Laurentino-Acqua Acetosa; decimalLatitude: 41.804083; decimalLongitude: 12.473833; geodeticDatum: WGS84; coordinatePrecision: 0.0002; **Identification:** identifiedBy: M. Annessi; **Event:** samplingProtocol: entomological net; eventDate: 4/26/2025; habitat: non-irrigated arable land; **Record Level:** institutionID: Roma Tre University; collectionCode: Clepto-01
- **Type status:** Other material. **Occurrence:** recordedBy: M. Annessi; individualCount: 1; sex: 1 female; lifeStage: adult; occurrenceID: E5866C15-F094-5C03-A833-6A74735A17FA; **Taxon:** class: Insecta; order: Hymenoptera; family: Apidae; genus: Nomada; specificEpithet: femoralis; scientificNameAuthorship: Morawitz, 1868; **Location:** country: Italy; countryCode: IT; stateProvince: Roma; locality: Riserva Naturale Laurentino-Acqua Acetosa; decimalLatitude: 41.806583; decimalLongitude: 12.461083; geodeticDatum: WGS84; coordinatePrecision: 0.0002; **Identification:** identifiedBy: M. Annessi; **Event:** samplingProtocol: entomological net; eventDate: 4/26/2025; habitat: non-irrigated arable land; **Record Level:** institutionID: Roma Tre University; collectionCode: Clepto-01
- **Type status:** Other material. **Occurrence:** recordedBy: M. Annessi; individualCount: 1; sex: 1 male; lifeStage: adult; occurrenceID: FE77173D-F26E-5E54-ACE0-52C8B3DEC8C9; **Taxon:** class: Insecta; order: Hymenoptera; family: Apidae; genus: Nomada; specificEpithet: femoralis; scientificNameAuthorship: Morawitz, 1868; **Location:** country: Italy; countryCode: IT; stateProvince: Roma; locality: Parco Regionale Urbano del Pineto; decimalLatitude: 41.912056; decimalLongitude: 12.431611; geodeticDatum: WGS84; coordinatePrecision: 0.0002; **Identification:** identifiedBy: M. Annessi; **Event:** samplingProtocol: entomological net; eventDate: 5/7/2025; habitat: land principally occupied by agriculture with significant areas of natural vegetation; **Record Level:** institutionID: Roma Tre University; collectionCode: Clepto-01
- **Type status:** Other material. **Occurrence:** recordedBy: M. Annessi; individualCount: 1; sex: 1 female; lifeStage: adult; occurrenceID: 3851D701-4ABC-5F18-95C8-E78713ABFF7A; **Taxon:** class: Insecta; order: Hymenoptera; family: Apidae; genus: Nomada; specificEpithet: femoralis; scientificNameAuthorship: Morawitz, 1868; **Location:** country: Italy; countryCode: IT; stateProvince: Roma; locality: Riserva Naturale Statale del Litorale Romano; decimalLatitude: 41.884889; decimalLongitude: 12.269306; geodeticDatum: WGS84; coordinatePrecision: 0.0002; **Identification:** identifiedBy: M. Annessi; **Event:** samplingProtocol: entomological net; eventDate: 5/14/2025; habitat: non-irrigated arable land; **Record Level:** institutionID: Roma Tre University; collectionCode: Clepto-01
- **Type status:** Other material. **Occurrence:** recordedBy: M. Annessi; individualCount: 2; sex: 2 females; lifeStage: adult; occurrenceID: 2BDB2268-0F9A-57D5-A465-8D138D57D2C9; **Taxon:** class: Insecta; order: Hymenoptera; family: Apidae; genus: Nomada; specificEpithet: femoralis; scientificNameAuthorship: Morawitz, 1868; **Location:** country: Italy; countryCode: IT; stateProvince: Roma; locality: Riserva Naturale Statale del Litorale Romano; decimalLatitude: 41.866611; decimalLongitude: 12.289917; geodeticDatum: WGS84; coordinatePrecision: 0.0002; **Identification:** identifiedBy: M. Annessi; **Event:** samplingProtocol: entomological net; eventDate: 5/14/2025; habitat: land principally occupied by agriculture with significant areas of natural vegetation; **Record Level:** institutionID: Roma Tre University; collectionCode: Clepto-01
- **Type status:** Other material. **Occurrence:** recordedBy: M. Annessi; individualCount: 2; sex: 2 females; lifeStage: adult; occurrenceID: 756F3E07-9DFC-5298-A1A8-5AD49D7B5F68; **Taxon:** class: Insecta; order: Hymenoptera; family: Apidae; genus: Nomada; specificEpithet: femoralis; scientificNameAuthorship: Morawitz, 1868; **Location:** country: Italy; countryCode: IT; stateProvince: Roma; locality: Riserva Naturale Laurentino-Acqua Acetosa; decimalLatitude: 41.806417; decimalLongitude: 12.467139; geodeticDatum: WGS84; coordinatePrecision: 0.0002; **Identification:** identifiedBy: M. Annessi; **Event:** samplingProtocol: entomological net; eventDate: 5/16/2025; habitat: non-irrigated arable land; **Record Level:** institutionID: Roma Tre University; collectionCode: Clepto-01
- **Type status:** Other material. **Occurrence:** recordedBy: M. Annessi; individualCount: 9; sex: 9 females; lifeStage: adult; occurrenceID: 8F44AF99-FA03-578C-9DD8-27AD31E097B9; **Taxon:** class: Insecta; order: Hymenoptera; family: Apidae; genus: Nomada; specificEpithet: femoralis; scientificNameAuthorship: Morawitz, 1868; **Location:** country: Italy; countryCode: IT; stateProvince: Roma; locality: Riserva Naturale Laurentino-Acqua Acetosa; decimalLatitude: 41.806583; decimalLongitude: 12.461083; geodeticDatum: WGS84; coordinatePrecision: 0.0002; **Identification:** identifiedBy: M. Annessi; **Event:** samplingProtocol: entomological net; eventDate: 5/16/2025; habitat: non-irrigated arable land; **Record Level:** institutionID: Roma Tre University; collectionCode: Clepto-01
- **Type status:** Other material. **Occurrence:** recordedBy: M. Annessi; individualCount: 3; sex: 3 females; lifeStage: adult; occurrenceID: 2E79CA89-1CFE-5E5A-A8DE-A316A601FD20; **Taxon:** class: Insecta; order: Hymenoptera; family: Apidae; genus: Nomada; specificEpithet: femoralis; scientificNameAuthorship: Morawitz, 1868; **Location:** country: Italy; countryCode: IT; stateProvince: Roma; locality: Parco Regionale Urbano del Pineto; decimalLatitude: 41.918417; decimalLongitude: 12.428694; geodeticDatum: WGS84; coordinatePrecision: 0.0002; **Identification:** identifiedBy: M. Annessi; **Event:** samplingProtocol: entomological net; eventDate: 5/19/2025; habitat: land principally occupied by agriculture with significant areas of natural vegetation; **Record Level:** institutionID: Roma Tre University; collectionCode: Clepto-01
- **Type status:** Other material. **Occurrence:** recordedBy: M. Annessi; individualCount: 1; sex: 1 female; lifeStage: adult; occurrenceID: 861A2EE6-9849-5FE2-BAF1-6FF9F21A8472; **Taxon:** class: Insecta; order: Hymenoptera; family: Apidae; genus: Nomada; specificEpithet: femoralis; scientificNameAuthorship: Morawitz, 1868; **Location:** country: Italy; countryCode: IT; stateProvince: Roma; locality: Parco Regionale Urbano del Pineto; decimalLatitude: 41.918417; decimalLongitude: 12.428694; geodeticDatum: WGS84; coordinatePrecision: 0.0002; **Identification:** identifiedBy: M. Annessi; **Event:** samplingProtocol: entomological net; eventDate: 6/2/2025; habitat: land principally occupied by agriculture with significant areas of natural vegetation; **Record Level:** institutionID: Roma Tre University; collectionCode: Clepto-01

#### Nomada (Nomada) concolor

Schmiedeknecht, 1882

036173CB-8EF3-57A7-900A-54776ED6D139

##### Materials

- **Type status:** Other material. **Occurrence:** recordedBy: M. Annessi; individualCount: 4; sex: 3 males, 1 female; lifeStage: adult; occurrenceID: 29E21346-F3E2-55B6-902E-ABB133BB8EEB; **Taxon:** class: Insecta; order: Hymenoptera; family: Apidae; genus: Nomada; specificEpithet: concolor; scientificNameAuthorship: Schmiedeknecht, 1882; **Location:** country: Italy; countryCode: IT; stateProvince: Roma; locality: Riserva Naturale Laurentino-Acqua Acetosa; decimalLatitude: 41.806583; decimalLongitude: 12.461083; geodeticDatum: WGS84; coordinatePrecision: 0.0002; **Identification:** identifiedBy: M. Annessi; **Event:** samplingProtocol: entomological net; eventDate: 3/30/2025; habitat: non-irrigated arable land; **Record Level:** institutionID: Roma Tre University; collectionCode: Clepto-01
- **Type status:** Other material. **Occurrence:** recordedBy: M. Annessi; individualCount: 6; sex: 6 females; lifeStage: adult; occurrenceID: 3B6EA6F7-FFE1-5FBC-A4E0-09B04DF15CB8; **Taxon:** class: Insecta; order: Hymenoptera; family: Apidae; genus: Nomada; specificEpithet: concolor; scientificNameAuthorship: Schmiedeknecht, 1882; **Location:** country: Italy; countryCode: IT; stateProvince: Roma; locality: Riserva Naturale Statale del Litorale Romano; decimalLatitude: 41.884889; decimalLongitude: 12.269306; geodeticDatum: WGS84; coordinatePrecision: 0.0002; **Identification:** identifiedBy: M. Annessi; **Event:** samplingProtocol: entomological net; eventDate: 4/22/2025; habitat: non-irrigated arable land; **Record Level:** institutionID: Roma Tre University; collectionCode: Clepto-01
- **Type status:** Other material. **Occurrence:** recordedBy: M. Annessi; individualCount: 1; sex: 1 female; lifeStage: adult; occurrenceID: A59118E1-0CEB-5732-82AE-0602E3733235; **Taxon:** class: Insecta; order: Hymenoptera; family: Apidae; genus: Nomada; specificEpithet: concolor; scientificNameAuthorship: Schmiedeknecht, 1882; **Location:** country: Italy; countryCode: IT; stateProvince: Roma; locality: Riserva Naturale Statale del Litorale Romano; decimalLatitude: 41.884889; decimalLongitude: 12.269306; geodeticDatum: WGS84; coordinatePrecision: 0.0002; **Identification:** identifiedBy: M. Annessi; **Event:** samplingProtocol: entomological net; eventDate: 5/14/2025; habitat: non-irrigated arable land; **Record Level:** institutionID: Roma Tre University; collectionCode: Clepto-01

#### Nomada (Holonomada) sexfasciata

Panzer, 1799

7750B3E3-029D-5879-B058-A79D9F6A4F22

##### Materials

- **Type status:** Other material. **Occurrence:** recordedBy: M. Annessi; individualCount: 1; sex: 1 female; lifeStage: adult; occurrenceID: D131CAB8-BD9E-5A84-B374-C8E8006409E6; **Taxon:** class: Insecta; order: Hymenoptera; family: Apidae; genus: Nomada; specificEpithet: sexfasciata; scientificNameAuthorship: Panzer, 1799; **Location:** country: Italy; countryCode: IT; stateProvince: Roma; locality: Riserva Naturale Laurentino-Acqua Acetosa; decimalLatitude: 41.806417; decimalLongitude: 12.467139; geodeticDatum: WGS84; coordinatePrecision: 0.0002; **Identification:** identifiedBy: M. Annessi; **Event:** samplingProtocol: entomological net; eventDate: 3/30/2025; habitat: non-irrigated arable land; **Record Level:** institutionID: Roma Tre University; collectionCode: Clepto-01
- **Type status:** Other material. **Occurrence:** recordedBy: M. Annessi; individualCount: 1; sex: 1 male; lifeStage: adult; occurrenceID: 8BF12FD8-270E-5560-8715-1E7EE5498FCB; **Taxon:** class: Insecta; order: Hymenoptera; family: Apidae; genus: Nomada; specificEpithet: sexfasciata; scientificNameAuthorship: Panzer, 1799; **Location:** country: Italy; countryCode: IT; stateProvince: Roma; locality: Parco Regionale Urbano del Pineto; decimalLatitude: 41.918417; decimalLongitude: 12.428694; geodeticDatum: WGS84; coordinatePrecision: 0.0002; **Identification:** identifiedBy: M. Annessi; **Event:** samplingProtocol: entomological net; eventDate: 4/2/2025; habitat: land principally occupied by agriculture with significant areas of natural vegetation; **Record Level:** institutionID: Roma Tre University; collectionCode: Clepto-01
- **Type status:** Other material. **Occurrence:** recordedBy: M. Annessi; individualCount: 6; sex: 6 females; lifeStage: adult; occurrenceID: F8C2F925-A6F2-5112-B9FA-DFEAC8AA5D5C; **Taxon:** class: Insecta; order: Hymenoptera; family: Apidae; genus: Nomada; specificEpithet: sexfasciata; scientificNameAuthorship: Panzer, 1799; **Location:** country: Italy; countryCode: IT; stateProvince: Roma; locality: Riserva Naturale Laurentino-Acqua Acetosa; decimalLatitude: 41.806417; decimalLongitude: 12.467139; geodeticDatum: WGS84; coordinatePrecision: 0.0002; **Identification:** identifiedBy: M. Annessi; **Event:** samplingProtocol: entomological net; eventDate: 4/11/2025; habitat: non-irrigated arable land; **Record Level:** institutionID: Roma Tre University; collectionCode: Clepto-01
- **Type status:** Other material. **Occurrence:** recordedBy: M. Annessi; individualCount: 1; sex: 1 male; lifeStage: adult; occurrenceID: 96203ABB-A556-571D-93B2-89FB36D75221; **Taxon:** class: Insecta; order: Hymenoptera; family: Apidae; genus: Nomada; specificEpithet: sexfasciata; scientificNameAuthorship: Panzer, 1799; **Location:** country: Italy; countryCode: IT; stateProvince: Roma; locality: Parco Regionale Urbano del Pineto; decimalLatitude: 41.922833; decimalLongitude: 12.426278; geodeticDatum: WGS84; coordinatePrecision: 0.0002; **Identification:** identifiedBy: M. Annessi; **Event:** samplingProtocol: entomological net; eventDate: 4/16/2025; habitat: land principally occupied by agriculture with significant areas of natural vegetation; **Record Level:** institutionID: Roma Tre University; collectionCode: Clepto-01
- **Type status:** Other material. **Occurrence:** recordedBy: M. Annessi; individualCount: 2; sex: 2 females; lifeStage: adult; occurrenceID: CF6D8E37-4477-515A-AAAA-0B32A8E87A3D; **Taxon:** class: Insecta; order: Hymenoptera; family: Apidae; genus: Nomada; specificEpithet: sexfasciata; scientificNameAuthorship: Panzer, 1799; **Location:** country: Italy; countryCode: IT; stateProvince: Roma; locality: Riserva Naturale Laurentino-Acqua Acetosa; decimalLatitude: 41.806417; decimalLongitude: 12.467139; geodeticDatum: WGS84; coordinatePrecision: 0.0002; **Identification:** identifiedBy: M. Annessi; **Event:** samplingProtocol: entomological net; eventDate: 4/26/2025; habitat: non-irrigated arable land; **Record Level:** institutionID: Roma Tre University; collectionCode: Clepto-01

#### Nomada (Heminomada) fucata

Panzer, 1798

9724D52B-4C73-582F-B55F-7FC1AA17BA2D

##### Materials

- **Type status:** Other material. **Occurrence:** recordedBy: M. Annessi; individualCount: 1; sex: 1 male; lifeStage: adult; occurrenceID: BB4AB162-5970-578E-A52A-939E6DF89C14; **Taxon:** class: Insecta; order: Hymenoptera; family: Apidae; genus: Nomada; specificEpithet: fucata; scientificNameAuthorship: Panzer, 1798; **Location:** country: Italy; countryCode: IT; stateProvince: Roma; locality: Riserva Naturale Laurentino-Acqua Acetosa; decimalLatitude: 41.806583; decimalLongitude: 12.461083; geodeticDatum: WGS84; coordinatePrecision: 0.0002; **Identification:** identifiedBy: M. Annessi; **Event:** samplingProtocol: entomological net; eventDate: 3/30/2025; habitat: non-irrigated arable land; **Record Level:** institutionID: Roma Tre University; collectionCode: Clepto-01
- **Type status:** Other material. **Occurrence:** recordedBy: M. Annessi; individualCount: 1; sex: 1 female; lifeStage: adult; occurrenceID: 2EC02316-4CF8-5CA3-BC16-FD5132ED8ADC; **Taxon:** class: Insecta; order: Hymenoptera; family: Apidae; genus: Nomada; specificEpithet: fucata; scientificNameAuthorship: Panzer, 1798; **Location:** country: Italy; countryCode: IT; stateProvince: Roma; locality: Riserva Naturale Statale del Litorale Romano; decimalLatitude: 41.884889; decimalLongitude: 12.269306; geodeticDatum: WGS84; coordinatePrecision: 0.0002; **Identification:** identifiedBy: M. Annessi; **Event:** samplingProtocol: entomological net; eventDate: 4/5/2025; habitat: non-irrigated arable land; **Record Level:** institutionID: Roma Tre University; collectionCode: Clepto-01
- **Type status:** Other material. **Occurrence:** recordedBy: M. Annessi; individualCount: 1; sex: 1 male; lifeStage: adult; occurrenceID: 20CD941D-8EC7-521E-ACF5-25C049CD104D; **Taxon:** class: Insecta; order: Hymenoptera; family: Apidae; genus: Nomada; specificEpithet: fucata; scientificNameAuthorship: Panzer, 1798; **Location:** country: Italy; countryCode: IT; stateProvince: Roma; locality: Riserva Naturale Statale del Litorale Romano; decimalLatitude: 41.866611; decimalLongitude: 12.289917; geodeticDatum: WGS84; coordinatePrecision: 0.0002; **Identification:** identifiedBy: M. Annessi; **Event:** samplingProtocol: entomological net; eventDate: 4/5/2025; habitat: land principally occupied by agriculture with significant areas of natural vegetation; **Record Level:** institutionID: Roma Tre University; collectionCode: Clepto-01
- **Type status:** Other material. **Occurrence:** recordedBy: M. Annessi; individualCount: 1; sex: 1 female; lifeStage: adult; occurrenceID: 2AAA28CD-78E6-5477-A3B7-E6062D411C09; **Taxon:** class: Insecta; order: Hymenoptera; family: Apidae; genus: Nomada; specificEpithet: fucata; scientificNameAuthorship: Panzer, 1798; **Location:** country: Italy; countryCode: IT; stateProvince: Roma; locality: Riserva Naturale Laurentino-Acqua Acetosa; decimalLatitude: 41.806583; decimalLongitude: 12.461083; geodeticDatum: WGS84; coordinatePrecision: 0.0002; **Identification:** identifiedBy: M. Annessi; **Event:** samplingProtocol: entomological net; eventDate: 4/11/2025; habitat: non-irrigated arable land; **Record Level:** institutionID: Roma Tre University; collectionCode: Clepto-01

#### Nomada (Gestamen) verna

Schmiedeknecht, 1882

F9918CE9-79EA-5384-85AF-88AA0944C88B

##### Materials

- **Type status:** Other material. **Occurrence:** recordedBy: M. Annessi; individualCount: 1; sex: 1 female; lifeStage: adult; occurrenceID: F14183E0-9315-51B7-AEAE-6840D8F264F2; **Taxon:** class: Insecta; order: Hymenoptera; family: Apidae; genus: Nomada; specificEpithet: verna; scientificNameAuthorship: Schmiedeknecht, 1882; **Location:** country: Italy; countryCode: IT; stateProvince: Roma; locality: Riserva Naturale Laurentino-Acqua Acetosa; decimalLatitude: 41.806417; decimalLongitude: 12.467139; geodeticDatum: WGS84; coordinatePrecision: 0.0002; **Identification:** identifiedBy: M. Annessi; **Event:** samplingProtocol: entomological net; eventDate: 3/30/2025; habitat: non-irrigated arable land; **Record Level:** institutionID: Roma Tre University; collectionCode: Clepto-01
- **Type status:** Other material. **Occurrence:** recordedBy: M. Annessi; individualCount: 1; sex: 1 female; lifeStage: adult; occurrenceID: 0FA2670D-C36C-542F-BDF8-7F8CFEFB15FB; **Taxon:** class: Insecta; order: Hymenoptera; family: Apidae; genus: Nomada; specificEpithet: verna; scientificNameAuthorship: Schmiedeknecht, 1882; **Location:** country: Italy; countryCode: IT; stateProvince: Roma; locality: Riserva Naturale Laurentino-Acqua Acetosa; decimalLatitude: 41.806417; decimalLongitude: 12.467139; geodeticDatum: WGS84; coordinatePrecision: 0.0002; **Identification:** identifiedBy: M. Annessi; **Event:** samplingProtocol: entomological net; eventDate: 4/11/2025; habitat: non-irrigated arable land; **Record Level:** institutionID: Roma Tre University; collectionCode: Clepto-01
- **Type status:** Other material. **Occurrence:** recordedBy: M. Annessi; individualCount: 1; sex: 1 male; lifeStage: adult; occurrenceID: 858977B8-6D68-53E4-AFEF-B3BBA507B9A3; **Taxon:** class: Insecta; order: Hymenoptera; family: Apidae; genus: Nomada; specificEpithet: verna; scientificNameAuthorship: Schmiedeknecht, 1882; **Location:** country: Italy; countryCode: IT; stateProvince: Roma; locality: Riserva Naturale Laurentino-Acqua Acetosa; decimalLatitude: 41.806583; decimalLongitude: 12.461083; geodeticDatum: WGS84; coordinatePrecision: 0.0002; **Identification:** identifiedBy: M. Annessi; **Event:** samplingProtocol: entomological net; eventDate: 4/11/2025; habitat: non-irrigated arable land; **Record Level:** institutionID: Roma Tre University; collectionCode: Clepto-01
- **Type status:** Other material. **Occurrence:** recordedBy: M. Annessi; individualCount: 1; sex: 1 female; lifeStage: adult; occurrenceID: 52D7050C-DE35-5E2F-B6AE-E06EA0C01AB7; **Taxon:** class: Insecta; order: Hymenoptera; family: Apidae; genus: Nomada; specificEpithet: verna; scientificNameAuthorship: Schmiedeknecht, 1882; **Location:** country: Italy; countryCode: IT; stateProvince: Roma; locality: Riserva Naturale Statale del Litorale Romano; decimalLatitude: 41.884889; decimalLongitude: 12.269306; geodeticDatum: WGS84; coordinatePrecision: 0.0002; **Identification:** identifiedBy: M. Annessi; **Event:** samplingProtocol: entomological net; eventDate: 4/22/2025; habitat: non-irrigated arable land; **Record Level:** institutionID: Roma Tre University; collectionCode: Clepto-01

#### Nomada (Collicula) rubiginosa

Pérez, 1884

3D8A8192-9D0E-584F-883E-2EEA25876286

##### Materials

- **Type status:** Other material. **Occurrence:** recordedBy: M. Annessi; individualCount: 6; sex: 6 males; lifeStage: adult; occurrenceID: D516B210-9D4F-5B02-BEF2-5FEB3FAA272F; **Taxon:** class: Insecta; order: Hymenoptera; family: Apidae; genus: Nomada; specificEpithet: rubiginosa; scientificNameAuthorship: Pérez, 1884; **Location:** country: Italy; countryCode: IT; stateProvince: Roma; locality: Parco Regionale Urbano del Pineto; decimalLatitude: 41.912056; decimalLongitude: 12.431611; geodeticDatum: WGS84; coordinatePrecision: 0.0002; **Identification:** identifiedBy: M. Annessi; **Event:** samplingProtocol: entomological net; eventDate: 4/2/2025; habitat: land principally occupied by agriculture with significant areas of natural vegetation; **Record Level:** institutionID: Roma Tre University; collectionCode: Clepto-01
- **Type status:** Other material. **Occurrence:** recordedBy: M. Annessi; individualCount: 3; sex: 3 females; lifeStage: adult; occurrenceID: 30C7A70F-F0E7-5CAA-8D4C-475DA42537A5; **Taxon:** class: Insecta; order: Hymenoptera; family: Apidae; genus: Nomada; specificEpithet: rubiginosa; scientificNameAuthorship: Pérez, 1884; **Location:** country: Italy; countryCode: IT; stateProvince: Roma; locality: Parco Regionale Urbano del Pineto; decimalLatitude: 41.912056; decimalLongitude: 12.431611; geodeticDatum: WGS84; coordinatePrecision: 0.0002; **Identification:** identifiedBy: M. Annessi; **Event:** samplingProtocol: entomological net; eventDate: 4/16/2025; habitat: land principally occupied by agriculture with significant areas of natural vegetation; **Record Level:** institutionID: Roma Tre University; collectionCode: Clepto-01

#### Nomada (Nomada) fulvicornis

Fabricius, 1793

E072EF7E-F0EF-5847-85FE-093063AAE11F

##### Materials

- **Type status:** Other material. **Occurrence:** recordedBy: M. Annessi; individualCount: 1; sex: 1 female; lifeStage: adult; occurrenceID: 4EABB6DD-899C-5DBE-AE42-50F4BFE5ACED; **Taxon:** class: Insecta; order: Hymenoptera; family: Apidae; genus: Nomada; specificEpithet: fulvicornis; scientificNameAuthorship: Fabricius, 1793; **Location:** country: Italy; countryCode: IT; stateProvince: Roma; locality: Tenuta Presidenziale di Castelporziano; decimalLatitude: 41.685861; decimalLongitude: 12.435944; geodeticDatum: WGS84; coordinatePrecision: 0.0002; **Identification:** identifiedBy: M. Annessi; **Event:** samplingProtocol: entomological net; eventDate: 4/3/2025; habitat: pastures; **Record Level:** institutionID: Roma Tre University; collectionCode: Clepto-01
- **Type status:** Other material. **Occurrence:** recordedBy: M. Annessi; individualCount: 1; sex: 1 female; lifeStage: adult; occurrenceID: 646BA4D9-95C0-5C37-B0AE-B471C342F216; **Taxon:** class: Insecta; order: Hymenoptera; family: Apidae; genus: Nomada; specificEpithet: fulvicornis; scientificNameAuthorship: Fabricius, 1793; **Location:** country: Italy; countryCode: IT; stateProvince: Roma; locality: Tenuta Presidenziale di Castelporziano; decimalLatitude: 41.699500; decimalLongitude: 12.386389; geodeticDatum: WGS84; coordinatePrecision: 0.0002; **Identification:** identifiedBy: M. Annessi; **Event:** samplingProtocol: entomological net; eventDate: 4/3/2025; habitat: pastures; **Record Level:** institutionID: Roma Tre University; collectionCode: Clepto-01

#### Nomada (Mininomada) blepharipes

Schmiedeknecht, 1882

D904002D-09D5-5B5E-A1AA-079EDEA5751A

##### Materials

- **Type status:** Other material. **Occurrence:** recordedBy: M. Annessi; individualCount: 2; sex: 2 females; lifeStage: adult; occurrenceID: D38C070F-EE53-5C02-BA90-B5465ACD8DD9; **Taxon:** class: Insecta; order: Hymenoptera; family: Apidae; genus: Nomada; specificEpithet: blepharipes; scientificNameAuthorship: Schmiedeknecht, 1882; **Location:** country: Italy; countryCode: IT; stateProvince: Roma; locality: Riserva Naturale Statale del Litorale Romano; decimalLatitude: 41.884889; decimalLongitude: 12.269306; geodeticDatum: WGS84; coordinatePrecision: 0.0002; **Identification:** identifiedBy: M. Annessi; **Event:** samplingProtocol: entomological net; eventDate: 4/22/2025; habitat: non-irrigated arable land; **Record Level:** institutionID: Roma Tre University; collectionCode: Clepto-01
- **Type status:** Other material. **Occurrence:** recordedBy: M. Annessi; individualCount: 1; sex: 1 female; lifeStage: adult; occurrenceID: FCA44CEA-1AAA-5DF3-B6E9-57D757A44421; **Taxon:** class: Insecta; order: Hymenoptera; family: Apidae; genus: Nomada; specificEpithet: blepharipes; scientificNameAuthorship: Schmiedeknecht, 1882; **Location:** country: Italy; countryCode: IT; stateProvince: Roma; locality: Riserva Naturale Statale del Litorale Romano; decimalLatitude: 41.884889; decimalLongitude: 12.269306; geodeticDatum: WGS84; coordinatePrecision: 0.0002; **Identification:** identifiedBy: M. Annessi; **Event:** samplingProtocol: entomological net; eventDate: 6/13/2025; habitat: non-irrigated arable land; **Record Level:** institutionID: Roma Tre University; collectionCode: Clepto-01

#### Nomada (Mininomada) sheppardana

(Kirby, 1802)

97376A7F-A78C-593F-8C8E-53764432A7B9

##### Materials

- **Type status:** Other material. **Occurrence:** recordedBy: M. Annessi; individualCount: 2; sex: 1 male, 1 female; lifeStage: adult; occurrenceID: 7DB4FABB-6C89-54C9-BDE2-83ACDE969B34; **Taxon:** class: Insecta; order: Hymenoptera; family: Apidae; genus: Nomada; specificEpithet: sheppardana; scientificNameAuthorship: (Kirby, 1802); **Location:** country: Italy; countryCode: IT; stateProvince: Roma; locality: Riserva Naturale Laurentino-Acqua Acetosa; decimalLatitude: 41.806417; decimalLongitude: 12.467139; geodeticDatum: WGS84; coordinatePrecision: 0.0002; **Identification:** identifiedBy: M. Annessi, M. Mei; **Event:** samplingProtocol: entomological net; eventDate: 4/26/2025; habitat: non-irrigated arable land; **Record Level:** institutionID: Roma Tre University; collectionCode: Clepto-01
- **Type status:** Other material. **Occurrence:** recordedBy: M. Annessi; individualCount: 3; sex: 3 females; lifeStage: adult; occurrenceID: 0FA4A3DE-9575-59AA-8963-14E87E9BE796; **Taxon:** class: Insecta; order: Hymenoptera; family: Apidae; genus: Nomada; specificEpithet: sheppardana; scientificNameAuthorship: (Kirby, 1802); **Location:** country: Italy; countryCode: IT; stateProvince: Roma; locality: Parco Regionale Urbano del Pineto; decimalLatitude: 41.918417; decimalLongitude: 12.428694; geodeticDatum: WGS84; coordinatePrecision: 0.0002; **Identification:** identifiedBy: M. Annessi, M. Mei; **Event:** samplingProtocol: entomological net; eventDate: 5/7/2025; habitat: land principally occupied by agriculture with significant areas of natural vegetation; **Record Level:** institutionID: Roma Tre University; collectionCode: Clepto-01
- **Type status:** Other material. **Occurrence:** recordedBy: M. Annessi; individualCount: 4; sex: 4 females; lifeStage: adult; occurrenceID: BEFE6C1B-C637-53D7-BDBF-6EEBB3D2A6A4; **Taxon:** class: Insecta; order: Hymenoptera; family: Apidae; genus: Nomada; specificEpithet: sheppardana; scientificNameAuthorship: (Kirby, 1802); **Location:** country: Italy; countryCode: IT; stateProvince: Roma; locality: Tenuta Presidenziale di Castelporziano; decimalLatitude: 41.699500; decimalLongitude: 12.386389; geodeticDatum: WGS84; coordinatePrecision: 0.0002; **Identification:** identifiedBy: M. Annessi, M. Mei; **Event:** samplingProtocol: entomological net; eventDate: 5/8/2025; habitat: pastures; **Record Level:** institutionID: Roma Tre University; collectionCode: Clepto-01
- **Type status:** Other material. **Occurrence:** recordedBy: M. Annessi; individualCount: 7; sex: 7 females; lifeStage: adult; occurrenceID: 311FE114-8C75-563B-A6D4-F1B6B7AD256E; **Taxon:** class: Insecta; order: Hymenoptera; family: Apidae; genus: Nomada; specificEpithet: sheppardana; scientificNameAuthorship: (Kirby, 1802); **Location:** country: Italy; countryCode: IT; stateProvince: Roma; locality: Parco Regionale Urbano del Pineto; decimalLatitude: 41.918417; decimalLongitude: 12.428694; geodeticDatum: WGS84; coordinatePrecision: 0.0002; **Identification:** identifiedBy: M. Annessi, M. Mei; **Event:** samplingProtocol: entomological net; eventDate: 5/19/2025; habitat: land principally occupied by agriculture with significant areas of natural vegetation; **Record Level:** institutionID: Roma Tre University; collectionCode: Clepto-01
- **Type status:** Other material. **Occurrence:** recordedBy: M. Annessi; individualCount: 4; sex: 4 females; lifeStage: adult; occurrenceID: A9E9F2AE-828A-53A9-A586-99D540758A4E; **Taxon:** class: Insecta; order: Hymenoptera; family: Apidae; genus: Nomada; specificEpithet: sheppardana; scientificNameAuthorship: (Kirby, 1802); **Location:** country: Italy; countryCode: IT; stateProvince: Roma; locality: Parco Regionale Urbano del Pineto; decimalLatitude: 41.918417; decimalLongitude: 12.428694; geodeticDatum: WGS84; coordinatePrecision: 0.0002; **Identification:** identifiedBy: M. Annessi, M. Mei; **Event:** samplingProtocol: entomological net; eventDate: 6/2/2025; habitat: land principally occupied by agriculture with significant areas of natural vegetation; **Record Level:** institutionID: Roma Tre University; collectionCode: Clepto-01

#### Nomada (Collicula) tridentirostris

Dours, 1873

C736C163-D304-5510-863C-987C655ADE29

##### Materials

- **Type status:** Other material. **Occurrence:** recordedBy: M. Annessi; individualCount: 1; sex: 1 male; lifeStage: adult; occurrenceID: FFADF04A-6D0A-5E12-8A58-C00A735E9540; **Taxon:** class: Insecta; order: Hymenoptera; family: Apidae; genus: Nomada; specificEpithet: tridentirostris; scientificNameAuthorship: Dours, 1873; **Location:** country: Italy; countryCode: IT; stateProvince: Roma; locality: Riserva Naturale Statale del Litorale Romano; decimalLatitude: 41.866611; decimalLongitude: 12.289917; geodeticDatum: WGS84; coordinatePrecision: 0.0002; **Identification:** identifiedBy: M. Annessi; **Event:** samplingProtocol: entomological net; eventDate: 4/22/2025; habitat: land principally occupied by agriculture with significant areas of natural vegetation; **Record Level:** institutionID: Roma Tre University; collectionCode: Clepto-01
- **Type status:** Other material. **Occurrence:** recordedBy: M. Annessi; individualCount: 1; sex: 1 male; lifeStage: adult; occurrenceID: 9E98425E-4B44-5AFB-BAB9-A91F34C3F031; **Taxon:** class: Insecta; order: Hymenoptera; family: Apidae; genus: Nomada; specificEpithet: tridentirostris; scientificNameAuthorship: Dours, 1873; **Location:** country: Italy; countryCode: IT; stateProvince: Roma; locality: Riserva Naturale Statale del Litorale Romano; decimalLatitude: 41.866611; decimalLongitude: 12.289917; geodeticDatum: WGS84; coordinatePrecision: 0.0002; **Identification:** identifiedBy: M. Annessi; **Event:** samplingProtocol: entomological net; eventDate: 5/14/2025; habitat: land principally occupied by agriculture with significant areas of natural vegetation; **Record Level:** institutionID: Roma Tre University; collectionCode: Clepto-01
- **Type status:** Other material. **Occurrence:** recordedBy: M. Annessi; individualCount: 1; sex: 1 female; lifeStage: adult; occurrenceID: 51C7960F-B65B-5306-B750-C29384AD648D; **Taxon:** class: Insecta; order: Hymenoptera; family: Apidae; genus: Nomada; specificEpithet: tridentirostris; scientificNameAuthorship: Dours, 1873; **Location:** country: Italy; countryCode: IT; stateProvince: Roma; locality: Parco Regionale Urbano del Pineto; decimalLatitude: 41.912056; decimalLongitude: 12.431611; geodeticDatum: WGS84; coordinatePrecision: 0.0002; **Identification:** identifiedBy: M. Annessi; **Event:** samplingProtocol: entomological net; eventDate: 5/19/2025; habitat: land principally occupied by agriculture with significant areas of natural vegetation; **Record Level:** institutionID: Roma Tre University; collectionCode: Clepto-01
- **Type status:** Other material. **Occurrence:** recordedBy: M. Annessi; individualCount: 1; sex: 1 female; lifeStage: adult; occurrenceID: 75DF09EF-ECB5-5706-867A-CA3AA09F5606; **Taxon:** class: Insecta; order: Hymenoptera; family: Apidae; genus: Nomada; specificEpithet: tridentirostris; scientificNameAuthorship: Dours, 1873; **Location:** country: Italy; countryCode: IT; stateProvince: Roma; locality: Riserva Naturale Statale del Litorale Romano; decimalLatitude: 41.866611; decimalLongitude: 12.289917; geodeticDatum: WGS84; coordinatePrecision: 0.0002; **Identification:** identifiedBy: M. Annessi; **Event:** samplingProtocol: entomological net; eventDate: 5/26/2025; habitat: land principally occupied by agriculture with significant areas of natural vegetation; **Record Level:** institutionID: Roma Tre University; collectionCode: Clepto-01

#### Nomada (Gestamen) dira

Schmiedeknecht, 1882

ACE3292C-1D34-5D06-BECD-2C1E5D2BFC55

##### Materials

- **Type status:** Other material. **Occurrence:** recordedBy: M. Annessi; individualCount: 1; sex: 1 female; lifeStage: adult; occurrenceID: ABC49D50-0523-5E60-A0A1-C6E33C8F93E2; **Taxon:** class: Insecta; order: Hymenoptera; family: Apidae; genus: Nomada; specificEpithet: dira; scientificNameAuthorship: Schmiedeknecht, 1882; **Location:** country: Italy; countryCode: IT; stateProvince: Roma; locality: Riserva Naturale Laurentino-Acqua Acetosa; decimalLatitude: 41.806583; decimalLongitude: 12.461083; geodeticDatum: WGS84; coordinatePrecision: 0.0002; **Identification:** identifiedBy: M. Annessi; **Event:** samplingProtocol: entomological net; eventDate: 5/29/2025; habitat: non-irrigated arable land; **Record Level:** institutionID: Roma Tre University; collectionCode: Clepto-01

#### Nomada (Gestamen) bispinosa

Mocsáry, 1883

B8D54DEF-BF8F-57E0-9748-BC98642D1BEF

##### Materials

- **Type status:** Other material. **Occurrence:** recordedBy: M. Annessi; individualCount: 1; sex: 1 male; lifeStage: adult; occurrenceID: 6F3EB87E-9616-5B18-B348-F9A3EE443A3B; **Taxon:** class: Insecta; order: Hymenoptera; family: Apidae; genus: Nomada; specificEpithet: bispinosa; scientificNameAuthorship: Mocsáry, 1883; **Location:** country: Italy; countryCode: IT; stateProvince: Roma; locality: Riserva Naturale Laurentino-Acqua Acetosa; decimalLatitude: 41.806417; decimalLongitude: 12.467139; geodeticDatum: WGS84; coordinatePrecision: 0.0002; **Identification:** identifiedBy: M. Annessi; **Event:** samplingProtocol: entomological net; eventDate: 4/26/2025; habitat: non-irrigated arable land; **Record Level:** institutionID: Roma Tre University; collectionCode: Clepto-01

#### Nomada (Nomada) discrepans

Schmiedeknecht, 1882

505B0D8D-C1DE-505C-99D7-7C70C3919027

##### Materials

- **Type status:** Other material. **Occurrence:** recordedBy: M. Annessi; individualCount: 2; sex: 2 males; lifeStage: adult; occurrenceID: 044EEBC2-DAF0-515C-A63F-BFF70835E58F; **Taxon:** class: Insecta; order: Hymenoptera; family: Apidae; genus: Nomada; specificEpithet: discrepans; scientificNameAuthorship: Schmiedeknecht, 1882; **Location:** country: Italy; countryCode: IT; stateProvince: Roma; locality: Parco Regionale Urbano del Pineto; decimalLatitude: 41.918417; decimalLongitude: 12.428694; geodeticDatum: WGS84; coordinatePrecision: 0.0002; **Identification:** identifiedBy: M. Annessi; **Event:** samplingProtocol: entomological net; eventDate: 5/7/2025; habitat: land principally occupied by agriculture with significant areas of natural vegetation; **Record Level:** institutionID: Roma Tre University; collectionCode: Clepto-01

#### Nomada (Mininomada) connectens

Pérez, 1884

CB47F600-AFB0-552B-8C17-376B46050CFD

##### Materials

- **Type status:** Other material. **Occurrence:** recordedBy: M. Annessi; individualCount: 2; sex: 2 females; lifeStage: adult; occurrenceID: C79F7666-BE12-5483-A422-2290D8DD45F4; **Taxon:** class: Insecta; order: Hymenoptera; family: Apidae; genus: Nomada; specificEpithet: connectens; scientificNameAuthorship: Pérez, 1884; **Location:** country: Italy; countryCode: IT; stateProvince: Roma; locality: Riserva Naturale Statale del Litorale Romano; decimalLatitude: 41.884889; decimalLongitude: 12.269306; geodeticDatum: WGS84; coordinatePrecision: 0.0002; **Identification:** identifiedBy: M. Annessi; **Event:** samplingProtocol: entomological net; eventDate: 5/14/2025; habitat: non-irrigated arable land; **Record Level:** institutionID: Roma Tre University; collectionCode: Clepto-01

#### Nomada (Mininomada) minuscula

Noskiewicz, 1930

AF67445F-96D6-5BE7-8127-B5D9A140500B

##### Materials

- **Type status:** Other material. **Occurrence:** recordedBy: M. Annessi; individualCount: 1; sex: 1 female; lifeStage: adult; occurrenceID: 879AF076-8523-57F5-8D5C-3C81C21A6B4B; **Taxon:** class: Insecta; order: Hymenoptera; family: Apidae; genus: Nomada; specificEpithet: minuscula; scientificNameAuthorship: Noskiewicz, 1930; **Location:** country: Italy; countryCode: IT; stateProvince: Roma; locality: Riserva Naturale Statale del Litorale Romano; decimalLatitude: 41.884889; decimalLongitude: 12.269306; geodeticDatum: WGS84; coordinatePrecision: 0.0002; **Identification:** identifiedBy: M. Annessi; **Event:** samplingProtocol: entomological net; eventDate: 5/14/2025; habitat: non-irrigated arable land; **Record Level:** institutionID: Roma Tre University; collectionCode: Clepto-01
- **Type status:** Other material. **Occurrence:** recordedBy: M. Annessi; individualCount: 1; sex: 1 female; lifeStage: adult; occurrenceID: 1F18C273-EC90-5A51-ABE6-EAF19880EAB0; **Taxon:** class: Insecta; order: Hymenoptera; family: Apidae; genus: Nomada; specificEpithet: minuscula; scientificNameAuthorship: Noskiewicz, 1930; **Location:** country: Italy; countryCode: IT; stateProvince: Roma; locality: Parco Regionale Urbano del Pineto; decimalLatitude: 41.918417; decimalLongitude: 12.428694; geodeticDatum: WGS84; coordinatePrecision: 0.0002; **Identification:** identifiedBy: M. Annessi; **Event:** samplingProtocol: entomological net; eventDate: 5/19/2025; habitat: land principally occupied by agriculture with significant areas of natural vegetation; **Record Level:** institutionID: Roma Tre University; collectionCode: Clepto-01

#### Nomada (Gestamen) nausicaa

Schmiedeknecht, 1882

47AB6B36-4B79-5E3A-99E9-D06F028F9073

##### Materials

- **Type status:** Other material. **Occurrence:** recordedBy: M. Annessi; individualCount: 1; sex: 1 male; lifeStage: adult; occurrenceID: 3B25BBFB-D0DB-5DC0-BA15-712654D22D1A; **Taxon:** class: Insecta; order: Hymenoptera; family: Apidae; genus: Nomada; specificEpithet: nausicaa; scientificNameAuthorship: Schmiedeknecht, 1882; **Location:** country: Italy; countryCode: IT; stateProvince: Roma; locality: Tenuta Presidenziale di Castelporziano; decimalLatitude: 41.693528; decimalLongitude: 12.431889; geodeticDatum: WGS84; coordinatePrecision: 0.0002; **Identification:** identifiedBy: M. Annessi; **Event:** samplingProtocol: entomological net; eventDate: 5/8/2025; habitat: pastures; **Record Level:** institutionID: Roma Tre University; collectionCode: Clepto-01
- **Type status:** Other material. **Occurrence:** recordedBy: M. Annessi; individualCount: 1; sex: 1 female; lifeStage: adult; occurrenceID: 619D967F-0CA0-55C8-B56F-6CEF7E87798C; **Taxon:** class: Insecta; order: Hymenoptera; family: Apidae; genus: Nomada; specificEpithet: nausicaa; scientificNameAuthorship: Schmiedeknecht, 1882; **Location:** country: Italy; countryCode: IT; stateProvince: Roma; locality: Riserva Naturale Statale del Litorale Romano; decimalLatitude: 41.866611; decimalLongitude: 12.289917; geodeticDatum: WGS84; coordinatePrecision: 0.0002; **Identification:** identifiedBy: M. Annessi; **Event:** samplingProtocol: entomological net; eventDate: 5/14/2025; habitat: land principally occupied by agriculture with significant areas of natural vegetation; **Record Level:** institutionID: Roma Tre University; collectionCode: Clepto-01

#### Nomada (Gestamen) sanguinea

Smith, 1854

F0777637-8612-5BA8-B3B7-671664DF9DD0

##### Materials

- **Type status:** Other material. **Occurrence:** recordedBy: M. Annessi; individualCount: 1; sex: 1 male; lifeStage: adult; occurrenceID: FAA4E91E-7CC6-5777-9A97-04B7F23FB94B; **Taxon:** class: Insecta; order: Hymenoptera; family: Apidae; genus: Nomada; specificEpithet: sanguinea; scientificNameAuthorship: Smith, 1854; **Location:** country: Italy; countryCode: IT; stateProvince: Roma; locality: Riserva Naturale Statale del Litorale Romano; decimalLatitude: 41.868750; decimalLongitude: 12.298028; geodeticDatum: WGS84; coordinatePrecision: 0.0002; **Identification:** identifiedBy: M. Annessi; **Event:** samplingProtocol: entomological net; eventDate: 5/14/2025; habitat: land principally occupied by agriculture with significant areas of natural vegetation; **Record Level:** institutionID: Roma Tre University; collectionCode: Clepto-01

#### Nomada (Holonomada) basalis

Herrich-Schäffer, 1839

88ABA5F0-5C0D-545B-9C35-6FEB1B9D026F

##### Materials

- **Type status:** Other material. **Occurrence:** recordedBy: M. Annessi; individualCount: 2; sex: 2 females; lifeStage: adult; occurrenceID: 8A3133EA-EE87-545E-8DB2-A5EE1F1E3A12; **Taxon:** class: Insecta; order: Hymenoptera; family: Apidae; genus: Nomada; specificEpithet: basalis; scientificNameAuthorship: Herrich-Schäffer, 1839; **Location:** country: Italy; countryCode: IT; stateProvince: Roma; locality: Riserva Naturale Laurentino-Acqua Acetosa; decimalLatitude: 41.806583; decimalLongitude: 12.461083; geodeticDatum: WGS84; coordinatePrecision: 0.0002; **Identification:** identifiedBy: M. Annessi; **Event:** samplingProtocol: entomological net; eventDate: 5/16/2025; habitat: non-irrigated arable land; **Record Level:** institutionID: Roma Tre University; collectionCode: Clepto-01

#### Nomada (Nomada) cruenta

Schmiedeknecht, 1882

EA929FA6-675E-53E7-92B3-988C3FF6DA5A

##### Materials

- **Type status:** Other material. **Occurrence:** recordedBy: M. Annessi; individualCount: 2; sex: 2 females; lifeStage: adult; occurrenceID: 1CA694D7-B3D6-5478-B34E-6552E810911D; **Taxon:** class: Insecta; order: Hymenoptera; family: Apidae; genus: Nomada; specificEpithet: cruenta; scientificNameAuthorship: Schmiedeknecht, 1882; **Location:** country: Italy; countryCode: IT; stateProvince: Roma; locality: Riserva Naturale Statale del Litorale Romano; decimalLatitude: 41.884889; decimalLongitude: 12.269306; geodeticDatum: WGS84; coordinatePrecision: 0.0002; **Identification:** identifiedBy: M. Annessi; **Event:** samplingProtocol: entomological net; eventDate: 6/13/2025; habitat: non-irrigated arable land; **Record Level:** institutionID: Roma Tre University; collectionCode: Clepto-01

#### Nomada (Collicula) stigma

Fabricius, 1804

9AC18B5B-7C18-5A5E-AB6B-1BDEEB118966

##### Materials

- **Type status:** Other material. **Occurrence:** recordedBy: M. Annessi; individualCount: 2; sex: 2 females; lifeStage: adult; occurrenceID: EBF52B12-85AF-5E45-9407-68C278E18703; **Taxon:** class: Insecta; order: Hymenoptera; family: Apidae; genus: Nomada; specificEpithet: stigma; scientificNameAuthorship: Fabricius, 1804; **Location:** country: Italy; countryCode: IT; stateProvince: Roma; locality: Riserva Naturale Statale del Litorale Romano; decimalLatitude: 41.866611; decimalLongitude: 12.289917; geodeticDatum: WGS84; coordinatePrecision: 0.0002; **Identification:** identifiedBy: M. Annessi, M. Mei; **Event:** samplingProtocol: entomological net; eventDate: 5/26/2025; habitat: land principally occupied by agriculture with significant areas of natural vegetation; **Record Level:** institutionID: Roma Tre University; collectionCode: Clepto-01

#### Nomada (Mininomada) distinguenda

Morawitz, 1873

A36F3135-6194-519D-8331-C6039E9D23D7

##### Materials

- **Type status:** Other material. **Occurrence:** recordedBy: M. Annessi; individualCount: 1; sex: 1 male; lifeStage: adult; occurrenceID: 609B3B93-8A79-5B64-9890-70420DF56E7D; **Taxon:** class: Insecta; order: Hymenoptera; family: Apidae; genus: Nomada; specificEpithet: distinguenda; scientificNameAuthorship: Morawitz, 1873; **Location:** country: Italy; countryCode: IT; stateProvince: Roma; locality: Riserva Naturale Statale del Litorale Romano; decimalLatitude: 41.868750; decimalLongitude: 12.298028; geodeticDatum: WGS84; coordinatePrecision: 0.0002; **Identification:** identifiedBy: M. Annessi; **Event:** samplingProtocol: entomological net; eventDate: 5/26/2025; habitat: land principally occupied by agriculture with significant areas of natural vegetation; **Record Level:** institutionID: Roma Tre University; collectionCode: Clepto-01
- **Type status:** Other material. **Occurrence:** recordedBy: M. Annessi; individualCount: 2; sex: 2 females; lifeStage: adult; occurrenceID: 4AED4CD2-AFE2-50B3-BCF7-6A924FDA7167; **Taxon:** class: Insecta; order: Hymenoptera; family: Apidae; genus: Nomada; specificEpithet: distinguenda; scientificNameAuthorship: Morawitz, 1873; **Location:** country: Italy; countryCode: IT; stateProvince: Roma; locality: Riserva Naturale Statale del Litorale Romano; decimalLatitude: 41.884889; decimalLongitude: 12.269306; geodeticDatum: WGS84; coordinatePrecision: 0.0002; **Identification:** identifiedBy: M. Annessi; **Event:** samplingProtocol: entomological net; eventDate: 7/25/2025; habitat: non-irrigated arable land; **Record Level:** institutionID: Roma Tre University; collectionCode: Clepto-01

#### Nomada (Nomada) piccioliana

Magretti, 1883

C600D7B8-0BD8-5E9F-9DFB-21D6E5B0CCE6

##### Materials

- **Type status:** Other material. **Occurrence:** recordedBy: M. Annessi; individualCount: 1; sex: 1 female; lifeStage: adult; occurrenceID: 2675A7A4-ED20-52A1-B642-E5949667868E; **Taxon:** class: Insecta; order: Hymenoptera; family: Apidae; genus: Nomada; specificEpithet: piccioliana; scientificNameAuthorship: Magretti, 1883; **Location:** country: Italy; countryCode: IT; stateProvince: Roma; locality: Riserva Naturale Statale del Litorale Romano; decimalLatitude: 41.868750; decimalLongitude: 12.298028; geodeticDatum: WGS84; coordinatePrecision: 0.0002; **Identification:** identifiedBy: M. Annessi; **Event:** samplingProtocol: entomological net; eventDate: 5/26/2025; habitat: land principally occupied by agriculture with significant areas of natural vegetation; **Record Level:** institutionID: Roma Tre University; collectionCode: Clepto-01

#### Nomada (Mininomada) bluethgeni

Stöckhert, 1944

7CD75C32-7A8C-5F55-9D0A-2D6F0CB5D480

##### Materials

- **Type status:** Other material. **Occurrence:** recordedBy: M. Annessi; individualCount: 1; sex: 1 male; lifeStage: adult; occurrenceID: 6877C8F1-B0BF-5482-9B12-F458C9BEAE41; **Taxon:** class: Insecta; order: Hymenoptera; family: Apidae; genus: Nomada; specificEpithet: bluethgeni; scientificNameAuthorship: Stöckhert, 1944; **Location:** country: Italy; countryCode: IT; stateProvince: Roma; locality: Riserva Naturale Statale del Litorale Romano; decimalLatitude: 41.859389; decimalLongitude: 12.294889; geodeticDatum: WGS84; coordinatePrecision: 0.0002; **Identification:** identifiedBy: M. Annessi; **Event:** samplingProtocol: entomological net; eventDate: 5/26/2025; habitat: land principally occupied by agriculture with significant areas of natural vegetation; **Record Level:** institutionID: Roma Tre University; collectionCode: Clepto-01

#### Thyreus histrionicus

(Illiger, 1806)

314D552F-0E9F-5F6F-9074-E9FBEBDE2C76

##### Materials

- **Type status:** Other material. **Occurrence:** recordedBy: M. Annessi; individualCount: 2; sex: 1 male, 1 female; lifeStage: adult; occurrenceID: 1E2E0D3A-B694-5355-B72C-FD33A9D1A9C1; **Taxon:** class: Insecta; order: Hymenoptera; family: Apidae; genus: Thyreus; specificEpithet: histrionicus; scientificNameAuthorship: (Illiger, 1806); **Location:** country: Italy; countryCode: IT; stateProvince: Roma; locality: Parco Regionale Urbano del Pineto; decimalLatitude: 41.918417; decimalLongitude: 12.428694; geodeticDatum: WGS84; coordinatePrecision: 0.0002; **Identification:** identifiedBy: M. Annessi; **Event:** samplingProtocol: entomological net; eventDate: 8/18/2025; habitat: land principally occupied by agriculture with significant areas of natural vegetation; **Record Level:** institutionID: Roma Tre University; collectionCode: Clepto-01
- **Type status:** Other material. **Occurrence:** recordedBy: M. Annessi; individualCount: 1; sex: 1 female; lifeStage: adult; occurrenceID: 586E0EDD-407A-57F4-8893-B35EB46FB698; **Taxon:** class: Insecta; order: Hymenoptera; family: Apidae; genus: Thyreus; specificEpithet: histrionicus; scientificNameAuthorship: (Illiger, 1806); **Location:** country: Italy; countryCode: IT; stateProvince: Roma; locality: Tenuta Presidenziale di Castelporziano; decimalLatitude: 41.693528; decimalLongitude: 12.431889; geodeticDatum: WGS84; coordinatePrecision: 0.0002; **Identification:** identifiedBy: M. Annessi; **Event:** samplingProtocol: entomological net; eventDate: 9/9/2025; habitat: pastures; **Record Level:** institutionID: Roma Tre University; collectionCode: Clepto-01
- **Type status:** Other material. **Occurrence:** recordedBy: M. Annessi; individualCount: 2; sex: 2 females; lifeStage: adult; occurrenceID: FB1A113A-C3CF-5670-929A-75CDF171DFBA; **Taxon:** class: Insecta; order: Hymenoptera; family: Apidae; genus: Thyreus; specificEpithet: histrionicus; scientificNameAuthorship: (Illiger, 1806); **Location:** country: Italy; countryCode: IT; stateProvince: Roma; locality: Parco Regionale Urbano del Pineto; decimalLatitude: 41.912056; decimalLongitude: 12.431611; geodeticDatum: WGS84; coordinatePrecision: 0.0002; **Identification:** identifiedBy: M. Annessi; **Event:** samplingProtocol: entomological net; eventDate: 9/12/2025; habitat: land principally occupied by agriculture with significant areas of natural vegetation; **Record Level:** institutionID: Roma Tre University; collectionCode: Clepto-01
- **Type status:** Other material. **Occurrence:** recordedBy: M. Annessi; individualCount: 2; sex: 2 females; lifeStage: adult; occurrenceID: B68DE36B-3E8B-5D76-8EF0-C389453D03F2; **Taxon:** class: Insecta; order: Hymenoptera; family: Apidae; genus: Thyreus; specificEpithet: histrionicus; scientificNameAuthorship: (Illiger, 1806); **Location:** country: Italy; countryCode: IT; stateProvince: Roma; locality: Riserva Naturale Statale del Litorale Romano; decimalLatitude: 41.884889; decimalLongitude: 12.269306; geodeticDatum: WGS84; coordinatePrecision: 0.0002; **Identification:** identifiedBy: M. Annessi; **Event:** samplingProtocol: entomological net; eventDate: 9/18/2025; habitat: non-irrigated arable land; **Record Level:** institutionID: Roma Tre University; collectionCode: Clepto-01

#### Thyreus ramosus

(Lepeletier, 1841)

80FD9044-1644-59BD-BBD2-51BB46FA4EA1

##### Materials

- **Type status:** Other material. **Occurrence:** recordedBy: M. Annessi; individualCount: 1; sex: 1 female; lifeStage: adult; occurrenceID: DA719F6C-0ABB-50FC-A757-D51E6FD12715; **Taxon:** class: Insecta; order: Hymenoptera; family: Apidae; genus: Thyreus; specificEpithet: ramosus; scientificNameAuthorship: (Lepeletier, 1841); **Location:** country: Italy; countryCode: IT; stateProvince: Roma; locality: Tenuta Presidenziale di Castelporziano; decimalLatitude: 41.693528; decimalLongitude: 12.431889; geodeticDatum: WGS84; coordinatePrecision: 0.0002; **Identification:** identifiedBy: M. Annessi; **Event:** samplingProtocol: entomological net; eventDate: 9/9/2025; habitat: pastures; **Record Level:** institutionID: Roma Tre University; collectionCode: Clepto-01

#### Epeolus julliani

Pérez, 1884

A036CA7D-52E3-5900-90BF-15E63C68F4CC

##### Materials

- **Type status:** Other material. **Occurrence:** recordedBy: M. Annessi; individualCount: 4; sex: 4 females; lifeStage: adult; occurrenceID: DF5D1DD2-2865-55BB-BB31-97D566E330A5; **Taxon:** class: Insecta; order: Hymenoptera; family: Apidae; genus: Epeolus; specificEpithet: julliani; scientificNameAuthorship: Pérez, 1884; **Location:** country: Italy; countryCode: IT; stateProvince: Roma; locality: Riserva Naturale Statale del Litorale Romano; decimalLatitude: 41.884889; decimalLongitude: 12.269306; geodeticDatum: WGS84; coordinatePrecision: 0.0002; **Identification:** identifiedBy: M. Annessi; **Event:** samplingProtocol: entomological net; eventDate: 9/18/2025; habitat: non-irrigated arable land; **Record Level:** institutionID: Roma Tre University; collectionCode: Clepto-01

#### Pasites maculatus

Jurine, 1807

B380795F-8089-56CC-9F1B-F261122AB176

##### Materials

- **Type status:** Other material. **Occurrence:** recordedBy: M. Annessi; individualCount: 1; sex: 1 female; lifeStage: adult; occurrenceID: E997DD09-923E-563A-97D1-BDFDEEDF1145; **Taxon:** class: Insecta; order: Hymenoptera; family: Apidae; genus: Pasites; specificEpithet: maculatus; scientificNameAuthorship: Jurine, 1807; **Location:** country: Italy; countryCode: IT; stateProvince: Roma; locality: Tenuta Presidenziale di Castelporziano; decimalLatitude: 41.699500; decimalLongitude: 12.386389; geodeticDatum: WGS84; coordinatePrecision: 0.0002; **Identification:** identifiedBy: M. Annessi; **Event:** samplingProtocol: entomological net; eventDate: 5/23/2025; habitat: pastures; **Record Level:** institutionID: Roma Tre University; collectionCode: Clepto-01

#### Bombus (Psithyrus) vestalis

(Geoffroy, 1785)

239D5341-0BB9-5589-B843-899D154B6844

##### Materials

- **Type status:** Other material. **Occurrence:** recordedBy: M. Annessi; individualCount: 1; sex: 1 female; lifeStage: adult; occurrenceID: 486F1F42-111C-55EC-88CA-0D9B17AD1096; **Taxon:** class: Insecta; order: Hymenoptera; family: Apidae; genus: Bombus; specificEpithet: vestalis; scientificNameAuthorship: (Geoffroy, 1785); **Location:** country: Italy; countryCode: IT; stateProvince: Roma; locality: Parco Regionale Urbano del Pineto; decimalLatitude: 41.922833; decimalLongitude: 12.426278; geodeticDatum: WGS84; coordinatePrecision: 0.0002; **Identification:** identifiedBy: M. Annessi, M. Mei; **Event:** samplingProtocol: entomological net; eventDate: 3/20/2025; habitat: land principally occupied by agriculture with significant areas of natural vegetation; **Record Level:** institutionID: Roma Tre University; collectionCode: Clepto-01

### Halictidae

#### Sphecodes puncticeps

Thomson, 1870

2866FA91-8912-5C17-AC0D-DDAF03A48CA0

##### Materials

- **Type status:** Other material. **Occurrence:** recordedBy: M. Annessi; individualCount: 2; sex: 2 females; lifeStage: adult; occurrenceID: 04CA2959-1F96-5096-B0F3-2AD610F3B38C; **Taxon:** class: Insecta; order: Hymenoptera; family: Halictidae; genus: Sphecodes; specificEpithet: puncticeps; scientificNameAuthorship: Thomson, 1870; **Location:** country: Italy; countryCode: IT; stateProvince: Roma; locality: Riserva Naturale Statale del Litorale Romano; decimalLatitude: 41.884889; decimalLongitude: 12.269306; geodeticDatum: WGS84; coordinatePrecision: 0.0002; **Identification:** identifiedBy: M. Annessi; **Event:** samplingProtocol: entomological net; eventDate: 4/22/2025; habitat: non-irrigated arable land; **Record Level:** institutionID: Roma Tre University; collectionCode: Clepto-01
- **Type status:** Other material. **Occurrence:** recordedBy: M. Annessi; individualCount: 1; sex: 1 female; lifeStage: adult; occurrenceID: F9C8B58C-F91E-5B1D-9621-C2CCD046DDA7; **Taxon:** class: Insecta; order: Hymenoptera; family: Halictidae; genus: Sphecodes; specificEpithet: puncticeps; scientificNameAuthorship: Thomson, 1870; **Location:** country: Italy; countryCode: IT; stateProvince: Roma; locality: Riserva Naturale Statale del Litorale Romano; decimalLatitude: 41.859389; decimalLongitude: 12.294889; geodeticDatum: WGS84; coordinatePrecision: 0.0002; **Identification:** identifiedBy: M. Annessi; **Event:** samplingProtocol: entomological net; eventDate: 4/22/2025; habitat: land principally occupied by agriculture with significant areas of natural vegetation; **Record Level:** institutionID: Roma Tre University; collectionCode: Clepto-01
- **Type status:** Other material. **Occurrence:** recordedBy: M. Annessi; individualCount: 1; sex: 1 female; lifeStage: adult; occurrenceID: 965D7B40-61DF-53DE-B7AE-1C3B388C4283; **Taxon:** class: Insecta; order: Hymenoptera; family: Halictidae; genus: Sphecodes; specificEpithet: puncticeps; scientificNameAuthorship: Thomson, 1870; **Location:** country: Italy; countryCode: IT; stateProvince: Roma; locality: Riserva Naturale Laurentino-Acqua Acetosa; decimalLatitude: 41.806583; decimalLongitude: 12.461083; geodeticDatum: WGS84; coordinatePrecision: 0.0002; **Identification:** identifiedBy: M. Annessi; **Event:** samplingProtocol: entomological net; eventDate: 4/26/2025; habitat: non-irrigated arable land; **Record Level:** institutionID: Roma Tre University; collectionCode: Clepto-01
- **Type status:** Other material. **Occurrence:** recordedBy: M. Annessi; individualCount: 2; sex: 2 females; lifeStage: adult; occurrenceID: 03D00B6A-8C3E-5FE0-AB3C-D036A35A6E81; **Taxon:** class: Insecta; order: Hymenoptera; family: Halictidae; genus: Sphecodes; specificEpithet: puncticeps; scientificNameAuthorship: Thomson, 1870; **Location:** country: Italy; countryCode: IT; stateProvince: Roma; locality: Riserva Naturale Statale del Litorale Romano; decimalLatitude: 41.884889; decimalLongitude: 12.269306; geodeticDatum: WGS84; coordinatePrecision: 0.0002; **Identification:** identifiedBy: M. Annessi; **Event:** samplingProtocol: entomological net; eventDate: 5/14/2025; habitat: non-irrigated arable land; **Record Level:** institutionID: Roma Tre University; collectionCode: Clepto-01
- **Type status:** Other material. **Occurrence:** recordedBy: M. Annessi; individualCount: 1; sex: 1 female; lifeStage: adult; occurrenceID: 65E433EA-0CC1-5123-B747-765EEF53AF5B; **Taxon:** class: Insecta; order: Hymenoptera; family: Halictidae; genus: Sphecodes; specificEpithet: puncticeps; scientificNameAuthorship: Thomson, 1870; **Location:** country: Italy; countryCode: IT; stateProvince: Roma; locality: Riserva Naturale Statale del Litorale Romano; decimalLatitude: 41.868750; decimalLongitude: 12.298028; geodeticDatum: WGS84; coordinatePrecision: 0.0002; **Identification:** identifiedBy: M. Annessi; **Event:** samplingProtocol: entomological net; eventDate: 5/14/2025; habitat: land principally occupied by agriculture with significant areas of natural vegetation; **Record Level:** institutionID: Roma Tre University; collectionCode: Clepto-01
- **Type status:** Other material. **Occurrence:** recordedBy: M. Annessi; individualCount: 1; sex: 1 female; lifeStage: adult; occurrenceID: 129C6B14-2585-57C4-B52F-9D74737FC752; **Taxon:** class: Insecta; order: Hymenoptera; family: Halictidae; genus: Sphecodes; specificEpithet: puncticeps; scientificNameAuthorship: Thomson, 1870; **Location:** country: Italy; countryCode: IT; stateProvince: Roma; locality: Riserva Naturale Statale del Litorale Romano; decimalLatitude: 41.859389; decimalLongitude: 12.294889; geodeticDatum: WGS84; coordinatePrecision: 0.0002; **Identification:** identifiedBy: M. Annessi; **Event:** samplingProtocol: entomological net; eventDate: 5/14/2025; habitat: land principally occupied by agriculture with significant areas of natural vegetation; **Record Level:** institutionID: Roma Tre University; collectionCode: Clepto-01
- **Type status:** Other material. **Occurrence:** recordedBy: M. Annessi; individualCount: 1; sex: 1 female; lifeStage: adult; occurrenceID: AAF39876-1A16-5DB1-847C-37148E2D081F; **Taxon:** class: Insecta; order: Hymenoptera; family: Halictidae; genus: Sphecodes; specificEpithet: puncticeps; scientificNameAuthorship: Thomson, 1870; **Location:** country: Italy; countryCode: IT; stateProvince: Roma; locality: Parco Regionale Urbano del Pineto; decimalLatitude: 41.918417; decimalLongitude: 12.428694; geodeticDatum: WGS84; coordinatePrecision: 0.0002; **Identification:** identifiedBy: M. Annessi; **Event:** samplingProtocol: entomological net; eventDate: 5/19/2025; habitat: land principally occupied by agriculture with significant areas of natural vegetation; **Record Level:** institutionID: Roma Tre University; collectionCode: Clepto-01
- **Type status:** Other material. **Occurrence:** recordedBy: M. Annessi; individualCount: 2; sex: 2 females; lifeStage: adult; occurrenceID: CE820C98-73E9-5C5E-8302-ADEE1F5C3A8E; **Taxon:** class: Insecta; order: Hymenoptera; family: Halictidae; genus: Sphecodes; specificEpithet: puncticeps; scientificNameAuthorship: Thomson, 1870; **Location:** country: Italy; countryCode: IT; stateProvince: Roma; locality: Riserva Naturale Statale del Litorale Romano; decimalLatitude: 41.884889; decimalLongitude: 12.269306; geodeticDatum: WGS84; coordinatePrecision: 0.0002; **Identification:** identifiedBy: M. Annessi; **Event:** samplingProtocol: entomological net; eventDate: 5/26/2025; habitat: non-irrigated arable land; **Record Level:** institutionID: Roma Tre University; collectionCode: Clepto-01
- **Type status:** Other material. **Occurrence:** recordedBy: M. Annessi; individualCount: 1; sex: 1 female; lifeStage: adult; occurrenceID: 07FAD7A2-BF1A-5CC9-983C-0323E5691B7C; **Taxon:** class: Insecta; order: Hymenoptera; family: Halictidae; genus: Sphecodes; specificEpithet: puncticeps; scientificNameAuthorship: Thomson, 1870; **Location:** country: Italy; countryCode: IT; stateProvince: Roma; locality: Riserva Naturale Statale del Litorale Romano; decimalLatitude: 41.868750; decimalLongitude: 12.298028; geodeticDatum: WGS84; coordinatePrecision: 0.0002; **Identification:** identifiedBy: M. Annessi; **Event:** samplingProtocol: entomological net; eventDate: 5/26/2025; habitat: land principally occupied by agriculture with significant areas of natural vegetation; **Record Level:** institutionID: Roma Tre University; collectionCode: Clepto-01
- **Type status:** Other material. **Occurrence:** recordedBy: M. Annessi; individualCount: 1; sex: 1 female; lifeStage: adult; occurrenceID: 7916EF9E-29E1-5320-9113-607D3A1AC306; **Taxon:** class: Insecta; order: Hymenoptera; family: Halictidae; genus: Sphecodes; specificEpithet: puncticeps; scientificNameAuthorship: Thomson, 1870; **Location:** country: Italy; countryCode: IT; stateProvince: Roma; locality: Riserva Naturale Statale del Litorale Romano; decimalLatitude: 41.884889; decimalLongitude: 12.269306; geodeticDatum: WGS84; coordinatePrecision: 0.0002; **Identification:** identifiedBy: M. Annessi; **Event:** samplingProtocol: entomological net; eventDate: 8/25/2025; habitat: non-irrigated arable land; **Record Level:** institutionID: Roma Tre University; collectionCode: Clepto-01
- **Type status:** Other material. **Occurrence:** recordedBy: M. Annessi; individualCount: 1; sex: 1 female; lifeStage: adult; occurrenceID: DFD2656A-D545-5A7D-9969-FAAEBB74FC0D; **Taxon:** class: Insecta; order: Hymenoptera; family: Halictidae; genus: Sphecodes; specificEpithet: puncticeps; scientificNameAuthorship: Thomson, 1870; **Location:** country: Italy; countryCode: IT; stateProvince: Roma; locality: Riserva Naturale Statale del Litorale Romano; decimalLatitude: 41.884889; decimalLongitude: 12.269306; geodeticDatum: WGS84; coordinatePrecision: 0.0002; **Identification:** identifiedBy: M. Annessi; **Event:** samplingProtocol: entomological net; eventDate: 9/18/2025; habitat: non-irrigated arable land; **Record Level:** institutionID: Roma Tre University; collectionCode: Clepto-01
- **Type status:** Other material. **Occurrence:** recordedBy: M. Annessi; individualCount: 2; sex: 2 females; lifeStage: adult; occurrenceID: F455A397-5434-55B3-8CBE-084352A2A2BD; **Taxon:** class: Insecta; order: Hymenoptera; family: Halictidae; genus: Sphecodes; specificEpithet: puncticeps; scientificNameAuthorship: Thomson, 1870; **Location:** country: Italy; countryCode: IT; stateProvince: Roma; locality: Riserva Naturale Statale del Litorale Romano; decimalLatitude: 41.868750; decimalLongitude: 12.298028; geodeticDatum: WGS84; coordinatePrecision: 0.0002; **Identification:** identifiedBy: M. Annessi; **Event:** samplingProtocol: entomological net; eventDate: 9/18/2025; habitat: land principally occupied by agriculture with significant areas of natural vegetation; **Record Level:** institutionID: Roma Tre University; collectionCode: Clepto-01

#### Sphecodes miniatus

von Hagens, 1882

1A8AF953-EE2E-5BF7-8FBF-23AEE8C6E5EA

##### Materials

- **Type status:** Other material. **Occurrence:** recordedBy: M. Annessi; individualCount: 1; sex: 1 female; lifeStage: adult; occurrenceID: 7DAF0FB5-00F2-5CD2-B570-478D397FC736; **Taxon:** class: Insecta; order: Hymenoptera; family: Halictidae; genus: Sphecodes; specificEpithet: miniatus; scientificNameAuthorship: von Hagens, 1882; **Location:** country: Italy; countryCode: IT; stateProvince: Roma; locality: Riserva Naturale Statale del Litorale Romano; decimalLatitude: 41.868750; decimalLongitude: 12.298028; geodeticDatum: WGS84; coordinatePrecision: 0.0002; **Identification:** identifiedBy: M. Annessi; **Event:** samplingProtocol: entomological net; eventDate: 4/22/2025; habitat: land principally occupied by agriculture with significant areas of natural vegetation; **Record Level:** institutionID: Roma Tre University; collectionCode: Clepto-01

#### Sphecodes ephippius

(Linnaeus, 1767)

B7D746FC-F3FB-5C00-877B-F10B13AE6A3B

##### Materials

- **Type status:** Other material. **Occurrence:** recordedBy: M. Annessi; individualCount: 1; sex: 1 female; lifeStage: adult; occurrenceID: 422779EA-DDD2-5175-A4DA-DC8EFF843C56; **Taxon:** class: Insecta; order: Hymenoptera; family: Halictidae; genus: Sphecodes; specificEpithet: ephippius; scientificNameAuthorship: (Linnaeus, 1767); **Location:** country: Italy; countryCode: IT; stateProvince: Roma; locality: Riserva Naturale Statale del Litorale Romano; decimalLatitude: 41.884889; decimalLongitude: 12.269306; geodeticDatum: WGS84; coordinatePrecision: 0.0002; **Identification:** identifiedBy: M. Annessi; **Event:** samplingProtocol: entomological net; eventDate: 4/5/2025; habitat: non-irrigated arable land; **Record Level:** institutionID: Roma Tre University; collectionCode: Clepto-01

#### Sphecodes gibbus

(Linnaeus, 1758)

55AAC27F-B1AC-5A90-8E17-D209FA494C11

##### Materials

- **Type status:** Other material. **Occurrence:** recordedBy: M. Annessi; individualCount: 1; sex: 1 female; lifeStage: adult; occurrenceID: AF4941CC-9CBF-5489-BED9-DF2BF3233168; **Taxon:** class: Insecta; order: Hymenoptera; family: Halictidae; genus: Sphecodes; specificEpithet: gibbus; scientificNameAuthorship: (Linnaeus, 1758); **Location:** country: Italy; countryCode: IT; stateProvince: Roma; locality: Riserva Naturale Statale del Litorale Romano; decimalLatitude: 41.884889; decimalLongitude: 12.269306; geodeticDatum: WGS84; coordinatePrecision: 0.0002; **Identification:** identifiedBy: M. Annessi, M. mei; **Event:** samplingProtocol: entomological net; eventDate: 5/14/2025; habitat: non-irrigated arable land; **Record Level:** institutionID: Roma Tre University; collectionCode: Clepto-01

#### Sphecodes rufiventris

(Panzer, 1798)

0F2655B8-F7CB-5506-AD7E-5D4BC63FF5F5

##### Materials

- **Type status:** Other material. **Occurrence:** recordedBy: M. Annessi; individualCount: 1; sex: 1 male; lifeStage: adult; occurrenceID: 8B089FE1-B53B-5B4D-909C-A906608251DF; **Taxon:** class: Insecta; order: Hymenoptera; family: Halictidae; genus: Sphecodes; specificEpithet: rufiventris; scientificNameAuthorship: (Panzer, 1798); **Location:** country: Italy; countryCode: IT; stateProvince: Roma; locality: Parco Regionale Urbano del Pineto; decimalLatitude: 41.918417; decimalLongitude: 12.428694; geodeticDatum: WGS84; coordinatePrecision: 0.0002; **Identification:** identifiedBy: M. Annessi; **Event:** samplingProtocol: entomological net; eventDate: 6/2/2025; habitat: land principally occupied by agriculture with significant areas of natural vegetation; **Record Level:** institutionID: Roma Tre University; collectionCode: Clepto-01

### Megachilidae

#### Coelioxys (Allocoelioxys) afer

Lepeletier, 1841

E3F7C4DE-5499-5F25-B15C-C9B5ACE63D43

##### Materials

- **Type status:** Other material. **Occurrence:** recordedBy: M. Annessi; individualCount: 1; sex: 1 female; lifeStage: adult; occurrenceID: 6B00DDFC-EBAE-5715-9A6C-4D7C3EC2A179; **Taxon:** class: Insecta; order: Hymenoptera; family: Megachilidae; genus: Coelioxys; specificEpithet: afer; scientificNameAuthorship: Lepeletier, 1841; **Location:** country: Italy; countryCode: IT; stateProvince: Roma; locality: Riserva Naturale Laurentino-Acqua Acetosa; decimalLatitude: 41.806417; decimalLongitude: 12.467139; geodeticDatum: WGS84; coordinatePrecision: 0.0002; **Identification:** identifiedBy: M. Annessi; **Event:** samplingProtocol: entomological net; eventDate: 8/22/2025; habitat: non-irrigated arable land; **Record Level:** institutionID: Roma Tre University; collectionCode: Clepto-01

#### Coelioxys (Paracoelioxys) elongatus

Lepeletier, 1841

DD70FBC6-D4A7-56FF-A349-E56F61C0C8D0

##### Materials

- **Type status:** Other material. **Occurrence:** recordedBy: M. Annessi; individualCount: 1; sex: 1 male; lifeStage: adult; occurrenceID: 34B1A6C5-505D-5407-B2D0-D501070A26F9; **Taxon:** class: Insecta; order: Hymenoptera; family: Megachilidae; genus: Coelioxys; specificEpithet: elongatus; scientificNameAuthorship: Lepeletier, 1841; **Location:** country: Italy; countryCode: IT; stateProvince: Roma; locality: Parco Regionale Urbano del Pineto; decimalLatitude: 41.918417; decimalLongitude: 12.428694; geodeticDatum: WGS84; coordinatePrecision: 0.0002; **Identification:** identifiedBy: M. Annessi; **Event:** samplingProtocol: entomological net; eventDate: 5/19/2025; habitat: land principally occupied by agriculture with significant areas of natural vegetation; **Record Level:** institutionID: Roma Tre University; collectionCode: Clepto-01

#### Coelioxys (Paracoelioxys) inermis

(Kirby, 1802)

B5BDC6FC-FEC2-5AA0-8EC5-4F436AD8F363

##### Materials

- **Type status:** Other material. **Occurrence:** recordedBy: M. Annessi; individualCount: 1; sex: 1 male; lifeStage: adult; occurrenceID: 93FBA1B8-B8E4-52A8-A205-AC2B5EDC8395; **Taxon:** class: Insecta; order: Hymenoptera; family: Megachilidae; genus: Coelioxys; specificEpithet: inermis; scientificNameAuthorship: (Kirby, 1802); **Location:** country: Italy; countryCode: IT; stateProvince: Roma; locality: Riserva Naturale Laurentino-Acqua Acetosa; decimalLatitude: 41.806583; decimalLongitude: 12.461083; geodeticDatum: WGS84; coordinatePrecision: 0.0002; **Identification:** identifiedBy: M. Annessi; **Event:** samplingProtocol: entomological net; eventDate: 8/22/2025; habitat: non-irrigated arable land; **Record Level:** institutionID: Roma Tre University; collectionCode: Clepto-01

#### Dioxys cinctus

(Jurine, 1807)

468F3B85-4EEC-5F7B-BA9B-FAC050BFE474

##### Materials

- **Type status:** Other material. **Occurrence:** recordedBy: M. Annessi; individualCount: 1; sex: 1 male; lifeStage: adult; occurrenceID: A77D1943-DAD0-5971-B3A8-22836BBFF1DD; **Taxon:** class: Insecta; order: Hymenoptera; family: Megachilidae; genus: Dioxys; specificEpithet: cinctus; scientificNameAuthorship: (Jurine, 1807); **Location:** country: Italy; countryCode: IT; stateProvince: Roma; locality: Riserva Naturale Statale del Litorale Romano; decimalLatitude: 41.859389; decimalLongitude: 12.294889; geodeticDatum: WGS84; coordinatePrecision: 0.0002; **Identification:** identifiedBy: M. Annessi; **Event:** samplingProtocol: entomological net; eventDate: 5/26/2025; habitat: land principally occupied by agriculture with significant areas of natural vegetation; **Record Level:** institutionID: Roma Tre University; collectionCode: Clepto-01
- **Type status:** Other material. **Occurrence:** recordedBy: M. Annessi; individualCount: 1; sex: 1 female; lifeStage: adult; occurrenceID: 7C26E919-15BC-59AD-905F-9F17E9438BA8; **Taxon:** class: Insecta; order: Hymenoptera; family: Megachilidae; genus: Dioxys; specificEpithet: cinctus; scientificNameAuthorship: (Jurine, 1807); **Location:** country: Italy; countryCode: IT; stateProvince: Roma; locality: Riserva Naturale Statale del Litorale Romano; decimalLatitude: 41.859389; decimalLongitude: 12.294889; geodeticDatum: WGS84; coordinatePrecision: 0.0002; **Identification:** identifiedBy: M. Annessi; **Event:** samplingProtocol: entomological net; eventDate: 6/13/2025; habitat: land principally occupied by agriculture with significant areas of natural vegetation; **Record Level:** institutionID: Roma Tre University; collectionCode: Clepto-01

## Analysis

**Figure 2.**
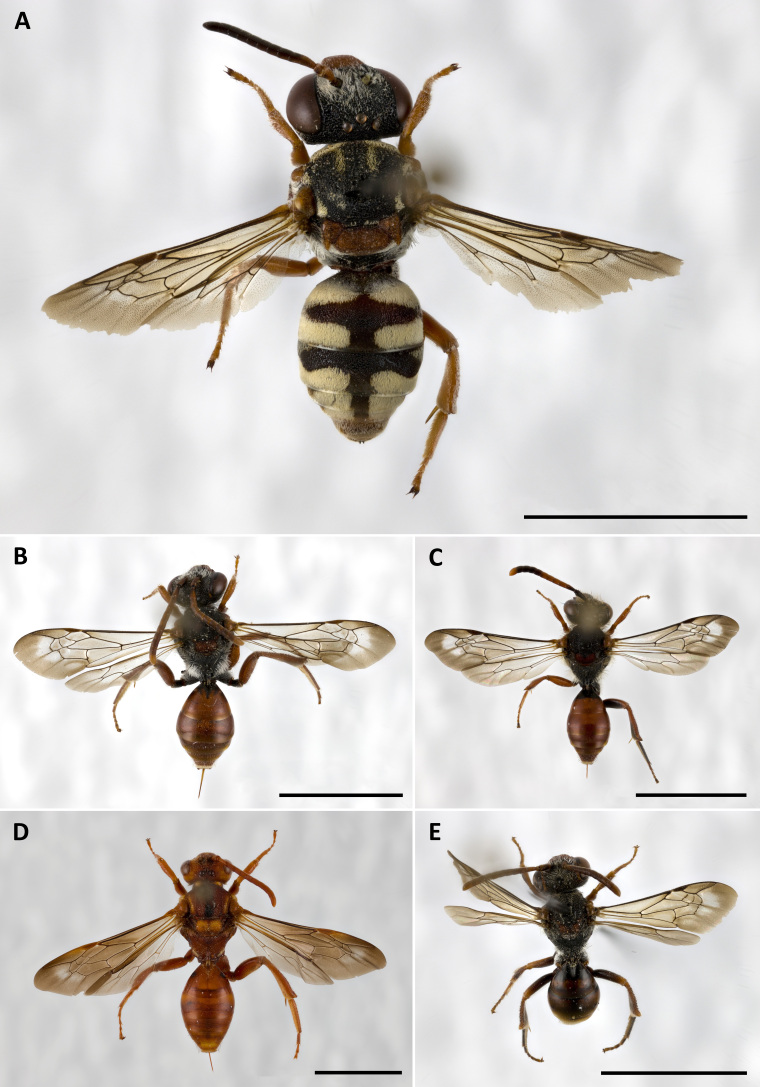
*Epeolus julliani* Pérez, 1884 **(A)**, *Nomada nausicaa* Schmiedeknecht, 1882 **(B)**, *Nomada carnifex* Mocsáry, 1883 **(C)**, *Nomada cruenta* Schmiedeknecht, 1882 **(D)**, *Nomada connectens* Pérez, 1884 **(E)** females collected on rural protected areas in Latium (Italy). Scale bars: 5 mm.

**Figure 3.**
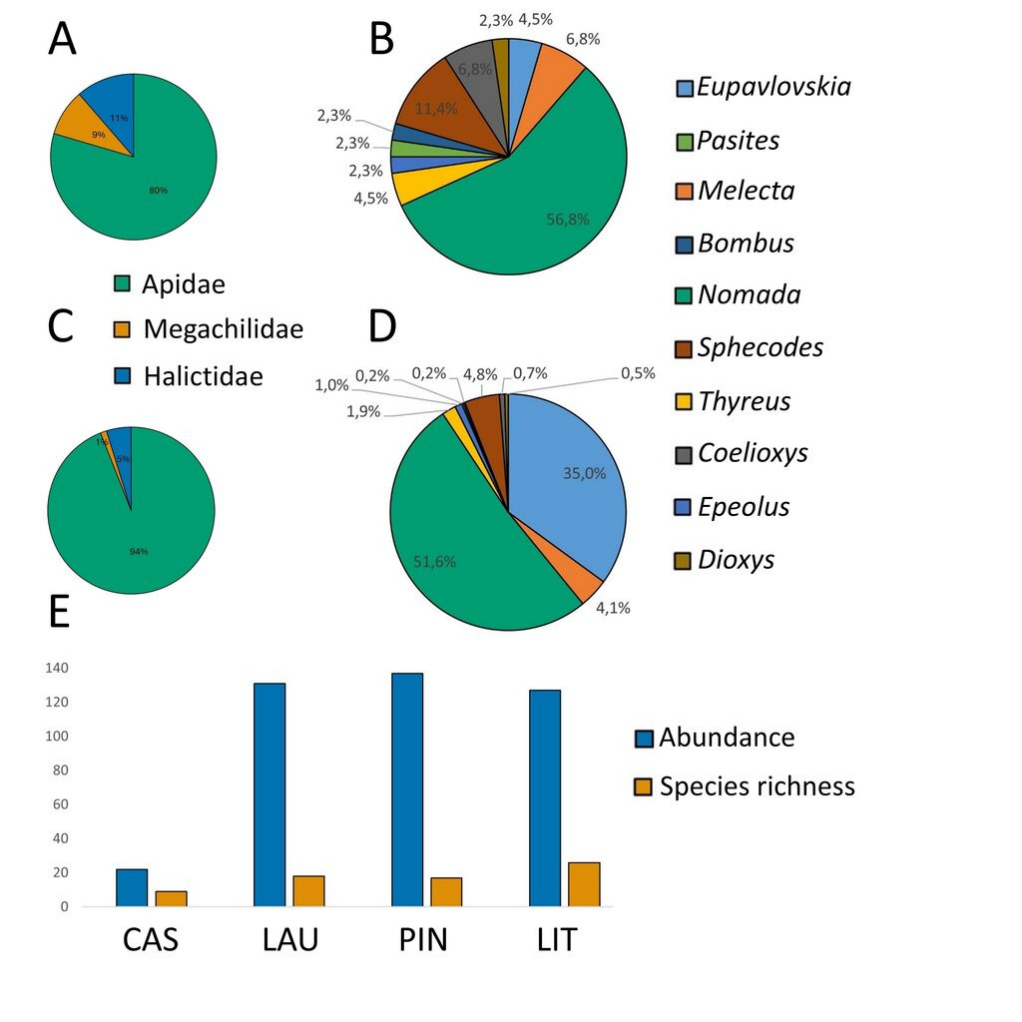
Assemblage of cleptoparasitic bees in the sampled sites. Taxonomic composition (% of species) of cleptoparasitic bees per family **(A)**. Taxonomic composition (% of species) of cleptoparasitic bees per genus **(B)**. Abundance (% of sampled individuals) of cleptoparasitic bees per family **(C)**. Abundance (% of sampled individuals) of cleptoparasitic bees per genus **(D)**. Abundance and species richness of cleptoparasitic bees recorded across the four sampled protected areas (**E**): Parco Regionale Urbano del Pineto (PIN), Riserva Naturale Laurentino-Acqua Acetosa (LAU), Riserva Naturale Statale del Litorale Romano (LIT), and Tenuta Presidenziale di Castelporziano (CAS).

A total of 417 individuals belonging to 44 different species from 10 genera and three families were collected (Suppl. material 2: Table S6). Five species are reported for the first time from Latium: *Epeolus julliani* Pérez, 1884 (Fig. 2A), *Nomada nausicaa* Schmiedeknecht, 1882 (Fig. 2B), *Nomada carnifex* Mocsáry, 1883 (Fig. 2C), *Nomada cruenta* Schmiedeknecht, 1882 (Fig. 2D), and *Nomada connectens* Pérez, 1884 (Fig. 2E), all belonging to the family Apidae. Apidae was the most represented family (35 species, 392 specimens), followed by Halictidae (5 species, 20 specimens) and Megachilidae (4 species, 5 specimens) (Fig. 3A,C). Within Apidae, at the genus level, *Nomada* Scopoli, 1770 was the most species-rich and abundant genus (25 species, 215 specimens), followed by *Eupavlovskia* Popov, 1955 in terms of abundance (146 specimens) (Fig. 3B,D), almost entirely represented by *Eupavlovskia obscura* (Friese, 1895), the most collected species in this study (141 specimens). Among the four sampled areas, the lowest species richness and abundance values were found in CAS (9 species, 22 specimens), while LAU and PIN showed the highest abundance (18 species, 131 specimens and 17 species, 137 specimens, respectively) and LIT the highest species richness (26 species, 127 specimens) (Fig. 3E).

Overall, 98 COI-5P sequences belonging to 43 different species were successfully sequenced (Suppl. material 3: Table S7). The NJ phylogenetic tree based only on our new sequences showed correspondence between monophyletic clades and morphological species, except for *Nomada sheppardana* (Kirby, 1802) and *Nomada minuscula* Noskiewicz, 1930, which were grouped in a single clade, and *Melecta leucorhyncha taormina* Gribodo, 1893, which is polyphyletic (Suppl. material 4: Fig. S1). Despite forming a monophyletic clade, one of the three analyzed specimens of *Melecta albifrons* (Forster, 1771) (POR2-10) exhibits noticeable genetic divergence from the remaining two (Suppl. material 4: Fig. S1). In addition, for the genera *Coelioxys* (Megachilidae), *Eupavlovskia*, *Epeolus*, *Melecta*, *Nomada* (Apidae) and *Sphecodes* (Halictidae), separate NJ trees were built to better clarify species-level identification (see below).

**Genus *Coelioxys***

**Figure 4.**
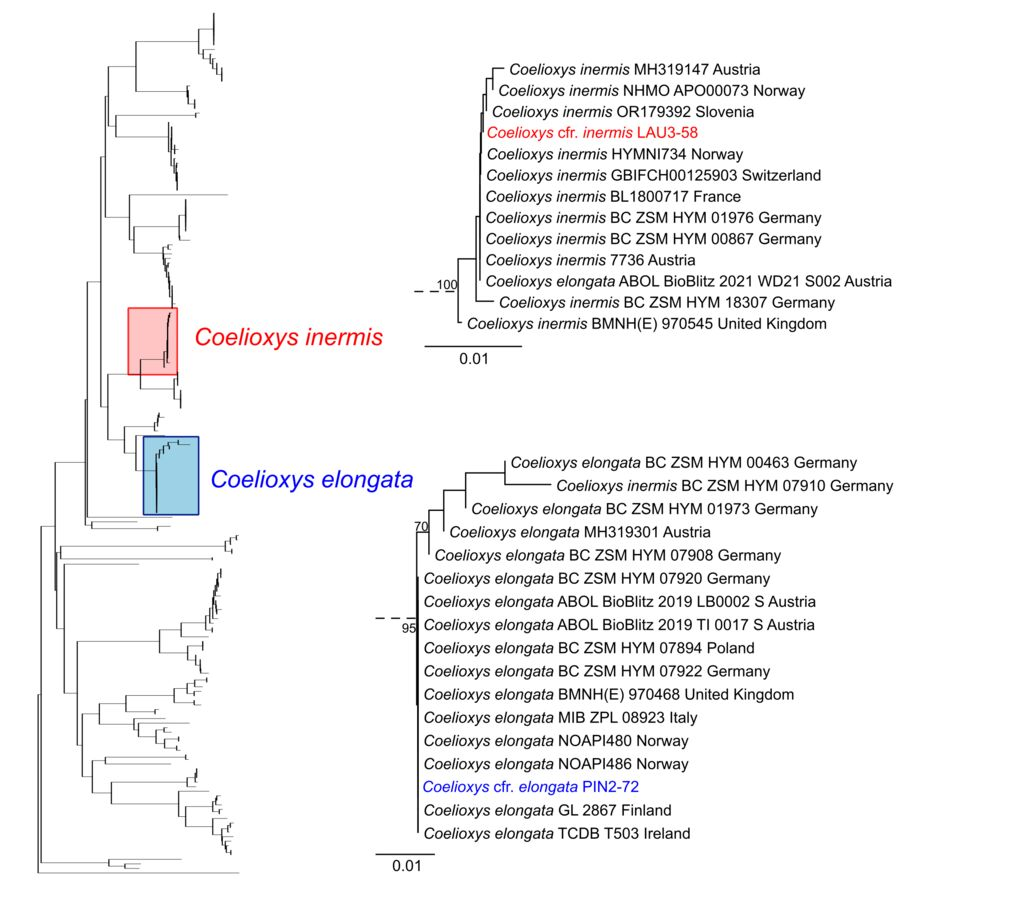
Neighbor-Joining Tree based on the COI-5P of *Coelioxys* showing the phylogenetic position of *C. inermis* and *C. elongata*. Colored taxa correspond to specimens newly sequenced for this work, while all the others were downloaded from BOLD. Only bootstrap values above 70 are shown at nodes (for a complete version of the tree see Suppl. material 4: Fig. S2).

*Coelioxys* cfr. *inermis* (LAU3-58) and *Coelioxys* cfr. *elongatus* (PIN2-72) formed a monophyletic clade with the conspecifics deposited in BOLD (bootstrap > 70; Fig. 4, Suppl. material 4: Fig. S2), showing 100% sequence similarity (Suppl. material 2: Table S6).

**Genus *Eupavlovskia***

**Figure 5.**
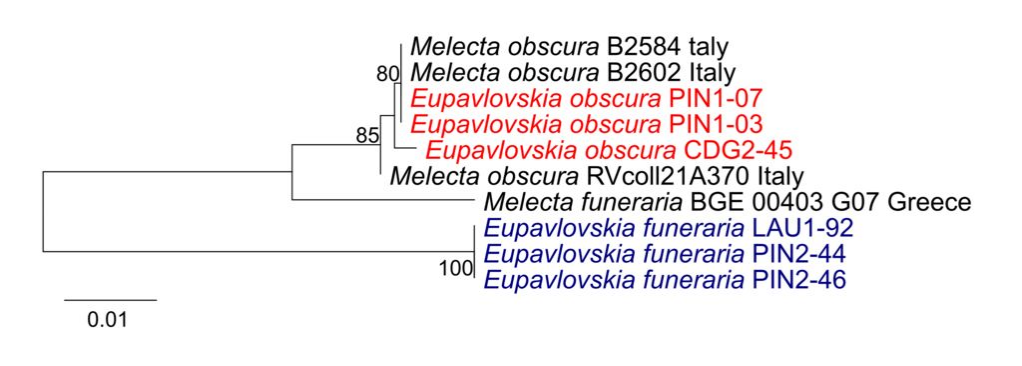
Neighbor-Joining Tree based on the COI-5P of *Eupavlovskia* showing the phylogenetic position of *E. obscura* and *E. funeraria*. Colored taxa correspond to specimens newly sequenced for this work, while all the others were downloaded from BOLD. Only bootstrap values above 70 are shown at nodes.

Our sequences of *Eupavlovskia funeraria* diverged from the only conspecific sequence available in the database (but bootstrap < 70; Fig. 5) consistent with the absence of close matches in BOLD (Suppl. material 2: Table S6).

**Genus *Epeolus***

**Figure 6.**
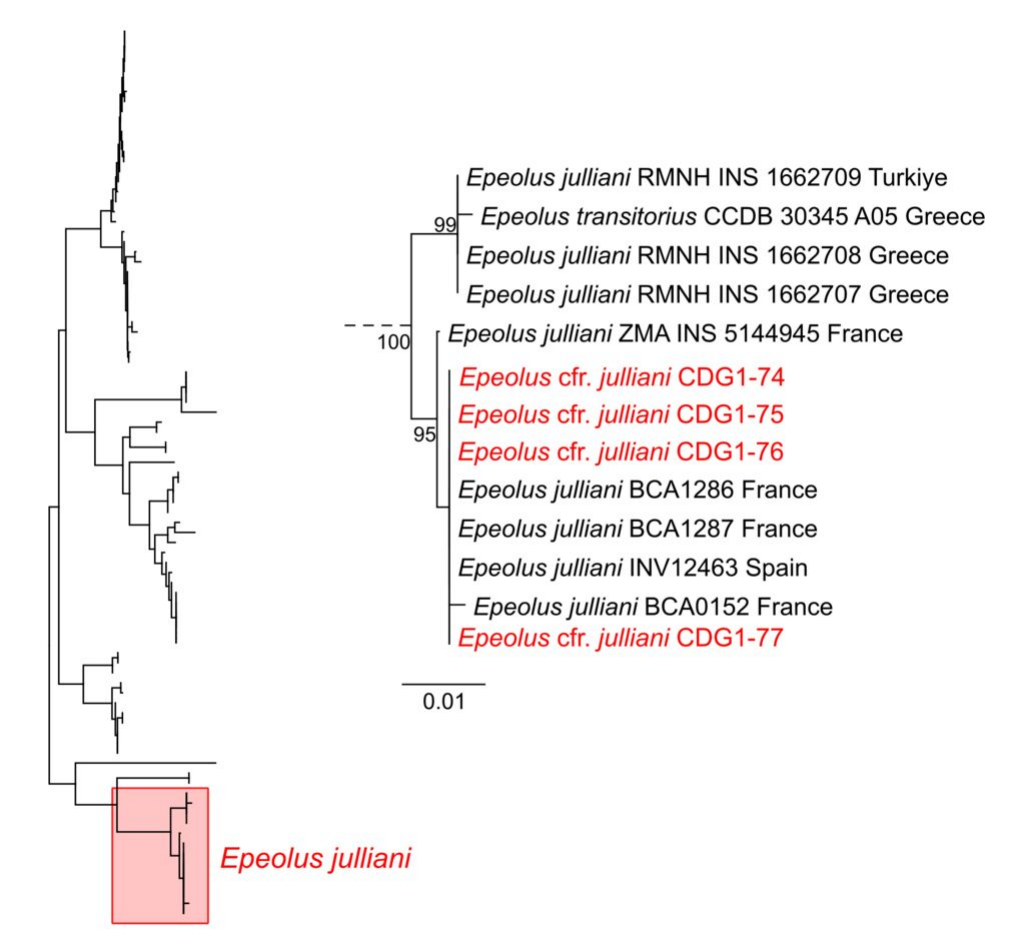
Neighbor-Joining Tree based on the COI-5P of *Epeolus* showing the phylogenetic position of *E. julliani*. Colored taxa correspond to specimens newly sequenced for this work, while all the others were downloaded from BOLD. Only bootstrap values above 70 are shown at nodes (for a complete version of the tree see Suppl. material 4: Fig. S3).

The four sequences of *Epeolus* cfr. *julliani* clustered with the conspecifics deposited in BOLD (bootstrap > 70; Fig. 6, Suppl. material 4: Fig. S3), exhibiting 100% sequence similarity (Suppl. material 2: Table S6).

**Genus *Melecta***

**Figure 7.**
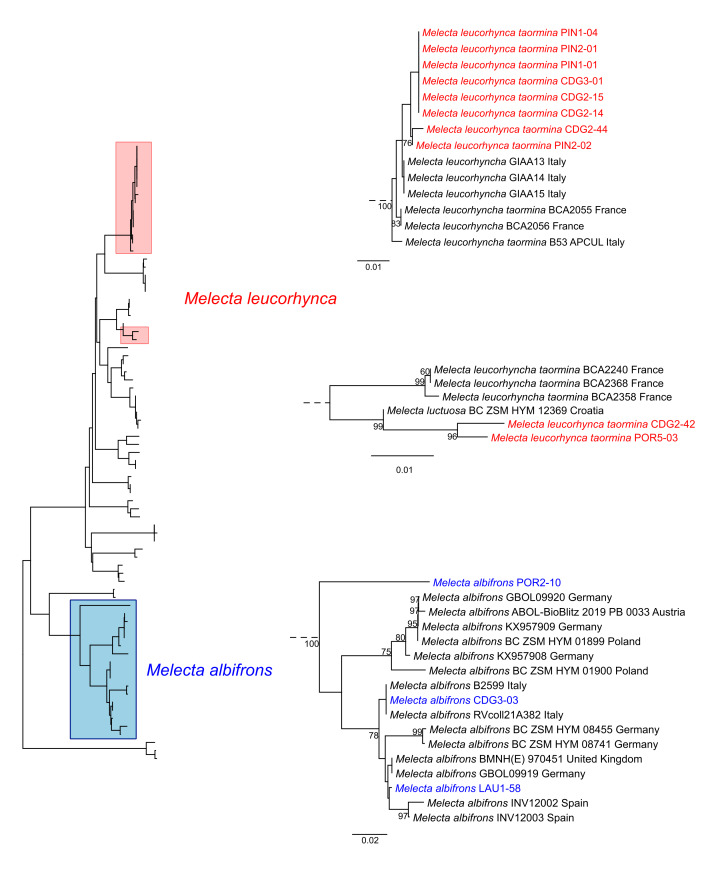
Neighbor-Joining Tree based on the COI-5P of *Melecta* showing the phylogenetic position of *M. leucorhyncha taormina* and *M. albifrons*. Colored taxa correspond to specimens newly sequenced for this work, while all the others were downloaded from BOLD. Only bootstrap values above 70 are shown at nodes (for a complete version of the tree see Suppl. material 4: Fig. S4).

*Melecta leucorhyncha* resulted polyphyletic and our specimens were sorted into two well supported clades (bootstrap >70) formed by specimens downloaded from BOLD (Fig. 7, Suppl. material 4: Fig. S4). Sequence similarity values among our specimens and those on BOLD ranged from 96.36% to 99.67% (Suppl. material 2: Table S6). Two of our sequences of *Melecta albifrons* (Forster, 1771) clustered within the conspecific specimens deposited in BOLD (bootstrap > 70), whereas the third sequence (POR2-10) formed a divergent lineage but closely related to the other *M. albifrons* (bootstrap > 70, Fig. 7, Suppl. material 4: Fig. S4). Sequence similarity values ranged from 94.35% to 100% (Suppl. material 2: Table S6).

**Genus *Nomada***

**Figure 8.**
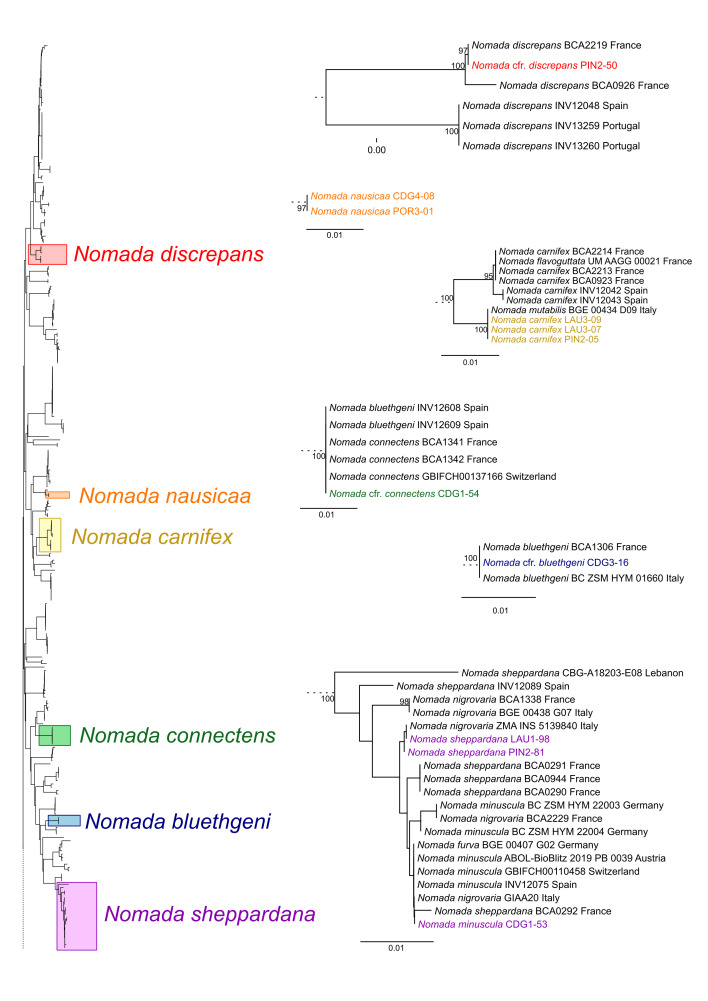
Neighbor-Joining Tree based on the COI-5P of *Nomada* showing the phylogenetic position of *N. discrepans, N. nausicaa*, *N. carnifex*, *N. connectens*, *N. bluethgeni*, and *N. sheppardana*. Colored taxa correspond to specimens newly sequenced for this work, while all the others were downloaded from BOLD. Only bootstrap values above 70 are shown at nodes (for a complete version of the tree see Suppl. material 4: Fig. S5).

**Figure 9.**
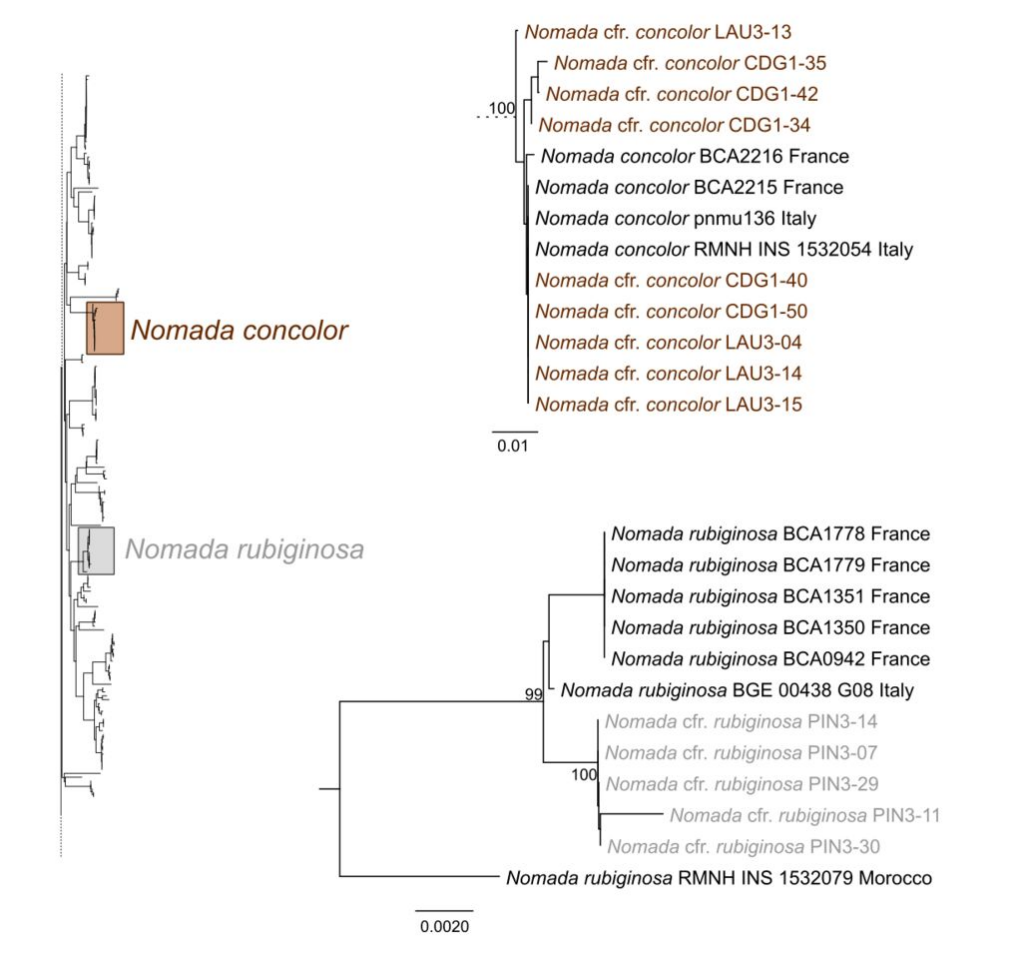
Neighbor-Joining Tree based on the COI-5P of *Nomada* showing the phylogenetic position of *N. concolor* and *N. rubiginosa*. Colored taxa correspond to specimens newly sequenced for this work, while all the others were downloaded from BOLD. Only bootstrap values above 70 are shown at nodes (for a complete version of the tree see Suppl. material 4: Fig. S5).

*Nomada* cfr. *discrepans* (PIN2-50) clustered with the conspecifics deposited in BOLD (bootstrap > 70; Fig. 8, Suppl. material 4: Fig. S5) and showed 100% sequence similarity with them (Suppl. material 2: Table S6). *Nomada nausicaa* (CDG4-08, POR3-01) formed a lineage close to *Nomada eos* Schmiedeknecht, 1882 (bootstrap > 70; Fig. 8, Suppl. material 4: Fig. S5), which was the best match in BOLD with 99.19% similarity (Suppl. material 2: Table S6). Our sequences of *Nomada carnifex* clustered with a specimen identified as *Nomada mutabilis* Morawitz, 1871 available in BOLD (bootstrap > 70; 99.67–99.84% similarity) and slightly diverged from the sister clade represented by deposited specimens of *N. carnifex* (Fig. 8, Suppl. material 4: Fig. S5; Suppl. material 2: Table S6). *Nomada* cfr. *connectens* (CDG1-54) clustered within the clade comprising specimens of *Nomada connectens* Pérez, 1884 and *Nomada bluethgeni* Stöckhert, 1944 deposited in BOLD (bootstrap < 70; Fig. 8, Suppl. material 4: Fig. S5), sharing 100% sequence similarity with both species (Suppl. material 2: Table S6). *Nomada* cfr. *bluethgeni* (CDG3-16) clustered within a second group composed of *N. bluethgeni* specimens (bootstrap < 70; 100% similarity; Fig. 8, Suppl. material 4: Fig. S5; Suppl. material 2: Table S6). Our sequences of *Nomada sheppardana* and *N. minuscula* clustered within the clade including other *N. sheppardana* and *N. nigrovaria* Pérez, 1895 (bootstrap > 70; Fig. 8, Suppl. material 4: Fig. S5). While *Nomada sheppardana* showed 100% sequence similarity with *N. nigrovaria*, *Nomada minuscula* showed 100% sequence similarity with both *N. minuscula* and *N. nigrovaria* (Suppl. material 2: Table S6). Our sequences of *Nomada* cfr. *concolor* formed a monophyletic clade with the conspecifics deposited in BOLD (bootstrap > 70; Fig. 9, Suppl. material 4: Fig. S5), showing from 99.66 to 100% sequence similarity (Suppl. material 2: Table S6). The five specimens identified as *Nomada* cfr. *rubiginosa* clustered with reference sequences of *N. rubiginosa* Pérez, 1884 available in BOLD (bootstrap > 70; Fig. 9, Suppl. material 4: Fig. S5), showing high sequence similarity (99.50–99.67%; Suppl. material 2: Table S6).

**Genus *Sphecodes***

**Figure 10.**
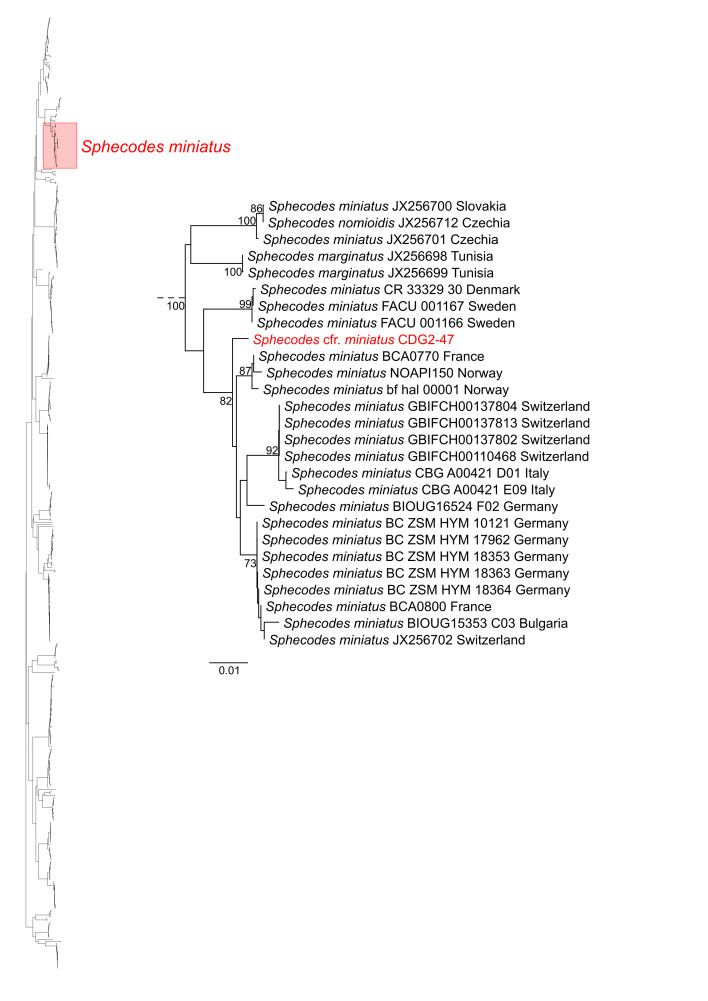
Neighbor-Joining Tree based on the COI-5P of *Sphecodes* showing the phylogenetic position of *Sphecodes miniatus*. Colored taxa correspond to specimens newly sequenced for this work, while all the others were downloaded from BOLD. Only bootstrap values above 70 are shown at nodes (for a complete version of the tree see Suppl. material 4: Fig. S6).

The sequence of *Sphecodes* cfr. *miniatus* (CDG2-47) clustered with the sequences of the conspecific deposited in BOLD (bootstrap > 70; Fig. 10, Suppl. material 4: Fig. S6) with 99.01% sequence similarity (Suppl. material 2: Table S6).

## Discussion

Our survey recorded approximately 40% of the cleptoparasitic bee species reported for the Latium region (Comba 2019) and 18% of those reported for Italy (Comba 2019, Reverté et al. 2023), even though sampling was conducted over a single year, using a single sampling method, and restricted to rural sites within four protected areas (see Fig. 1). Such diversity, together with the relatively high number of individuals collected, suggests that hand netting alone may represent a sufficiently effective method for sampling cleptoparasitic bees. This is consistent with Krahner et al. (2021), who demonstrated that hand netting outperforms pan traps and Malaise traps in sampling these bees. Trap nesting, although effective, was primarily driven by Megachilidae species, which are known to be particularly well-sampled by this method (Krahner et al. 2021), potentially explaining the relatively low number of cleptoparasitic Megachilidae collected in our work. However, recent studies using hand netting with comparable sampling effort — such as Fortini et al. (2024) in areas near our study sites and Annessi et al. (2025b) in Sardinia — recorded considerably fewer cleptoparasitic species than the present study, likely reflecting both the underrepresentation of parasitic bees in surveys of entire bee communities versus targeted investigations and potential sampler-dependent effects. The lowest species richness and abundance of cleptoparasitic bees were found in CAS, which included the highest number of transects (n = 5). This was followed by LIT (n = 4), which recorded the highest species richness, and LAU and PIN (n = 3 each), which showed relatively high and comparable species richness and abundance. These patterns may partly reflect differences in environmental conditions among study sites. However, it is important to consider that sampling effort was not evenly distributed among the four protected areas. Moreover, the relatively high cleptoparasitic bee diversity recorded at LAU and PIN — both located within the boundaries of the Grande Raccordo Anulare — suggests that sizeable agricultural areas embedded within the urban matrix may provide suitable conditions for these taxa.

In our work, Apidae was the most represented family, with *Nomada* accounting for more than half of the recorded species, consistent with Apidae being the family with the highest number of cleptoparasitic genera and *Nomada* the largest genus of brood parasites worldwide (Alexander and Schwarz 1994, Michener 2007, Litman et al. 2013). The cleptoparasitic bee community recorded in this study included both widely distributed species and taxa with restricted distribution. Among these, *Nomada rubiginosa* and *Nomada blepharipes* Schmiedeknecht, 1882 were assessed as moderate and critical risk of extinction, respectively, according to Comba (2019). In the present study, five species are newly recorded for Latium, with no previous records in regional or national checklists (Comba and Comba 1991, Comba 2019), recent studies carried out in the region (Geppert et al. 2022, Fortini et al. 2024), or GBIF (2026). These findings highlight how knowledge of wild bee distribution remains fragmented in the Mediterranean region, further supporting the need for continued faunistic surveys to improve species distribution data even at a local scale and contribute to more reliable assessments of their conservation status (Michez et al. 2026, Quaranta et al. 2018).

The integrative taxonomic approach, based on morphology and supported by molecular data, proved effective in achieving reliable species-level identifications across all examined specimens, particularly for the confirmation of taxa with uncertain morphological identification. New COI-5P sequences were generated and deposited in the BOLD and GenBank databases, including barcode sequences for *Nomada nausicaa*, representing the first COI-5P record for this species in both repositories. In addition, the combined morphological and molecular data highlighted some potential taxonomic issues that warrant further systematic investigation. Among these, the placement of *Melecta leucorhyncha* sequences into well-separated clades (Fig. 7, Suppl. material 4: Fig. S4) may reflect the presence of cryptic diversity within this genus. To the best of our knowledge, no integrative taxonomic study combining morphological and molecular data has yet been conducted on the genus *Melecta*. The last comprehensive morphological treatment dates back to Lieftinck (1981), who already highlighted the difficulty of establishing stable morphological characters for *Melecta leucorhyncha* across its range, and the risk of misidentification with the polytypic and morphologically similar *Melecta italica* Radoszkowski, 1876. Nevertheless, in our phylogenetic tree, *Melecta leucorhyncha* and *M. italica* appear clearly separated at the molecular level (Suppl. material 4: Fig. S4), although the limited number of available sequences, the undefined barcode gap within this genus, together with the possibility of errors in DNA data repositories (Janko et al. 2024), prevent any firm conclusions at this time. For this reason, we conservatively assign our specimens to *Melecta leucorhyncha taormina* based on the morphological characters reported by Lieftinck (1981) and the dichotomous key by Rasmont (2014), who recognized the melanic subspecies “*taormina”* Strand, confined to Italy and the major Tyrrhenian islands, pending comprehensive taxonomic revisions clarifying the taxonomy of the genus *Melecta*. Additional ambiguities within this genus concern *Melecta albifrons*, for which one of our sequences is separated from the other conspecifics in the NJ tree (Fig. 7, Suppl. material 4: Fig. S4), and *Eupavlovskia funeraria* — within *Eupavlovskia*, recently brought back to genus level from *Melecta* (Orr et al. 2024) — whose sequences show divergence from the only conspecific currently available in BOLD (Fig. 5). Nevertheless, these specimens were identified based on diagnostic morphological characters following Lieftinck (1969), Lieftinck (1981) and Rasmont (2014). Regarding the genus *Nomada*, in some cases (e.g., *N. carnifex*, *N. connectens*, *N. sheppardana*) our specimens clustered in clades that included both sequences of the morphologically identified conspecific species and sequences assigned to other species (Fig. 8, Suppl. material 4: Fig. S5). In certain cases, this can be attributed to possible misidentifications in public DNA repositories (Janko et al. 2024), whereas in others it may reflect BIN sharing among closely related species within the genus, as highlighted by Ollivier et al. (2025), for example in *Nomada sheppardana* and *N. nigrovaria*. However, in these cases, species names were assigned on the basis of morphological characters whenever these allowed a confident identification.

In conclusion, this work provides a contribution to the still limited knowledge of the distribution of cleptoparasitic bees in the Mediterranean region and improves current understanding of intraspecific genetic variability through the deposition of newly generated COI-5P sequences in the public databases. Moreover, our findings highlight some taxonomic issues and confirm the confusing taxonomic situation of some species within the genus *Melecta*.

## Supplementary Material

XML Treatment for Eupavlovskia obscura

XML Treatment for Eupavlovskia funeraria

XML Treatment for Melecta (Melecta) leucorhyncha taormina

XML Treatment for Melecta (Melecta) albifrons

XML Treatment for Melecta (Melecta) aegyptiaca

XML Treatment for Nomada (Gestamen) carnifex

XML Treatment for Nomada (Collicula) integra

XML Treatment for Nomada (Gestamen) femoralis

XML Treatment for Nomada (Nomada) concolor

XML Treatment for Nomada (Holonomada) sexfasciata

XML Treatment for Nomada (Heminomada) fucata

XML Treatment for Nomada (Gestamen) verna

XML Treatment for Nomada (Collicula) rubiginosa

XML Treatment for Nomada (Nomada) fulvicornis

XML Treatment for Nomada (Mininomada) blepharipes

XML Treatment for Nomada (Mininomada) sheppardana

XML Treatment for Nomada (Collicula) tridentirostris

XML Treatment for Nomada (Gestamen) dira

XML Treatment for Nomada (Gestamen) bispinosa

XML Treatment for Nomada (Nomada) discrepans

XML Treatment for Nomada (Mininomada) connectens

XML Treatment for Nomada (Mininomada) minuscula

XML Treatment for Nomada (Gestamen) nausicaa

XML Treatment for Nomada (Gestamen) sanguinea

XML Treatment for Nomada (Holonomada) basalis

XML Treatment for Nomada (Nomada) cruenta

XML Treatment for Nomada (Collicula) stigma

XML Treatment for Nomada (Mininomada) distinguenda

XML Treatment for Nomada (Nomada) piccioliana

XML Treatment for Nomada (Mininomada) bluethgeni

XML Treatment for Thyreus histrionicus

XML Treatment for Thyreus ramosus

XML Treatment for Epeolus julliani

XML Treatment for Pasites maculatus

XML Treatment for Bombus (Psithyrus) vestalis

XML Treatment for Sphecodes puncticeps

XML Treatment for Sphecodes miniatus

XML Treatment for Sphecodes ephippius

XML Treatment for Sphecodes gibbus

XML Treatment for Sphecodes rufiventris

XML Treatment for Coelioxys (Allocoelioxys) afer

XML Treatment for Coelioxys (Paracoelioxys) elongatus

XML Treatment for Coelioxys (Paracoelioxys) inermis

XML Treatment for Dioxys cinctus

FA247CC7-C120-5DA0-913D-486E4A910EC4 10.3897/BDJ.14.e193040.suppl1 Supplementary material 1 List of specimens Data type xlsx Brief description List of specimens used for the construction of Neighbor-Joining phylogenetic trees. File: oo_1564661.xlsx https://binary.pensoft.net/file/1564661 Annessi M., Riccieri A., Polidori C., Mei M., Di Giulio A.

C8A922BF-9B40-5068-AEB5-40C6105F3084 10.3897/BDJ.14.e193040.suppl2 Supplementary material 2 List of cleptoparasitic bee species Data type xlsx Brief description List of cleptoparasitic bee species sampled within four protected areas in the province of Rome, identified using an integrative taxonomy approach. File: oo_1565769.xlsx https://binary.pensoft.net/file/1565769 Annessi M., Riccieri A., Polidori C., Mei M., Di Giulio A.

2FC80759-8F49-5908-9A76-62729B8D35BB 10.3897/BDJ.14.e193040.suppl3 Supplementary material 3 List of sequenced specimens Data type xlsx Brief description List of sequenced specimens with reported Sample ID, Process ID, Institution Storing and Genbank accession. File: oo_1564660.xlsx https://binary.pensoft.net/file/1564660 Annessi M., Riccieri A., Polidori C., Mei M., Di Giulio A.

DB5B6350-51FA-591D-AAFE-E45DC1C315A9 10.3897/BDJ.14.e193040.suppl4 Supplementary material 4 Neighbor-Joining tree Data type PDF Brief description Neighbor-Joining tree based on the COI-5P. File: oo_1564683.pdf https://binary.pensoft.net/file/1564683 Annessi M., Riccieri A., Polidori C., Mei M., Di Giulio A.
