# Supplementary material for: Cleptoparasitic bees (Apoidea, Anthophila) in rural protected areas in Latium (Italy): a faunistic survey through an integrative taxonomic approach
Source: Biodivers Data J. 2026 Jul 14;14:e193040. doi: 10.3897/BDJ.14.e193040 (PMC13389566; doi:10.3897/BDJ.14.e193040)

Fig. S1
Neighbor Joining Tree based on the COI of all the specimens newly sequenced in this paper.
Only bootstrap values above 70 are shown at nodes.

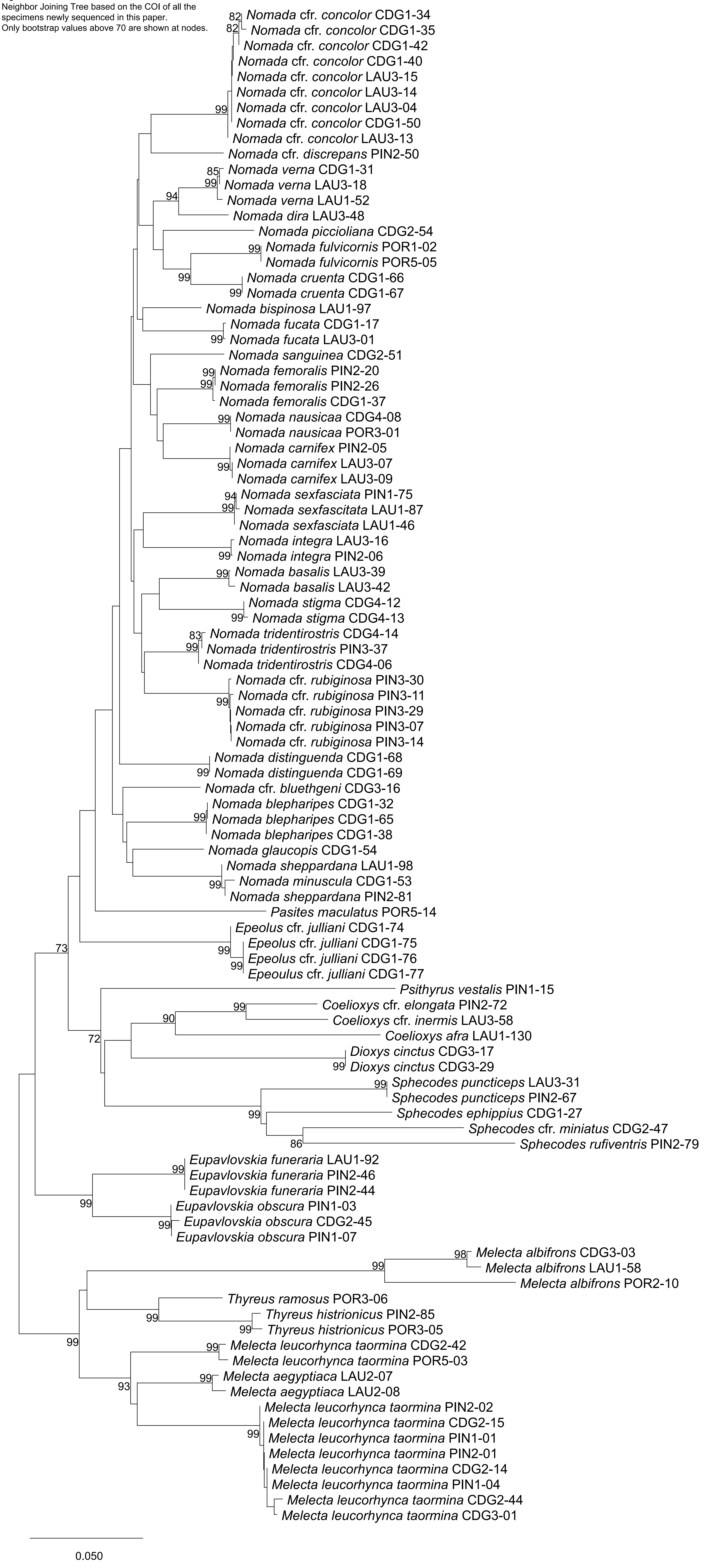

Fig. S2
Neighbor Joining Tree based on the COI of Coelioxys. Taxa shown in red correspond to specimens newly sequenced in this work, while all the others were downloaded from BOLD. Only bootstrap values above 70 are shown at nodes.

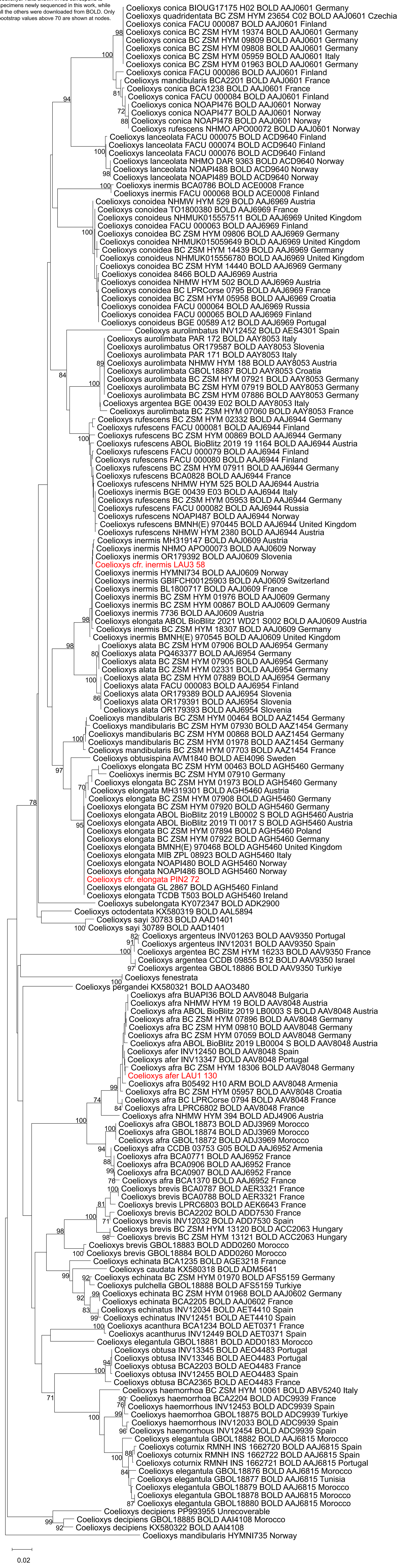

Fig. S3
Neighbor Joining Tree based on the COI of Epeolus. Taxa shown in red correspond to specimens newly sequenced in this work, while all the others were downloaded from BOLD. Only bootstrap values above 70 are shown at nodes.

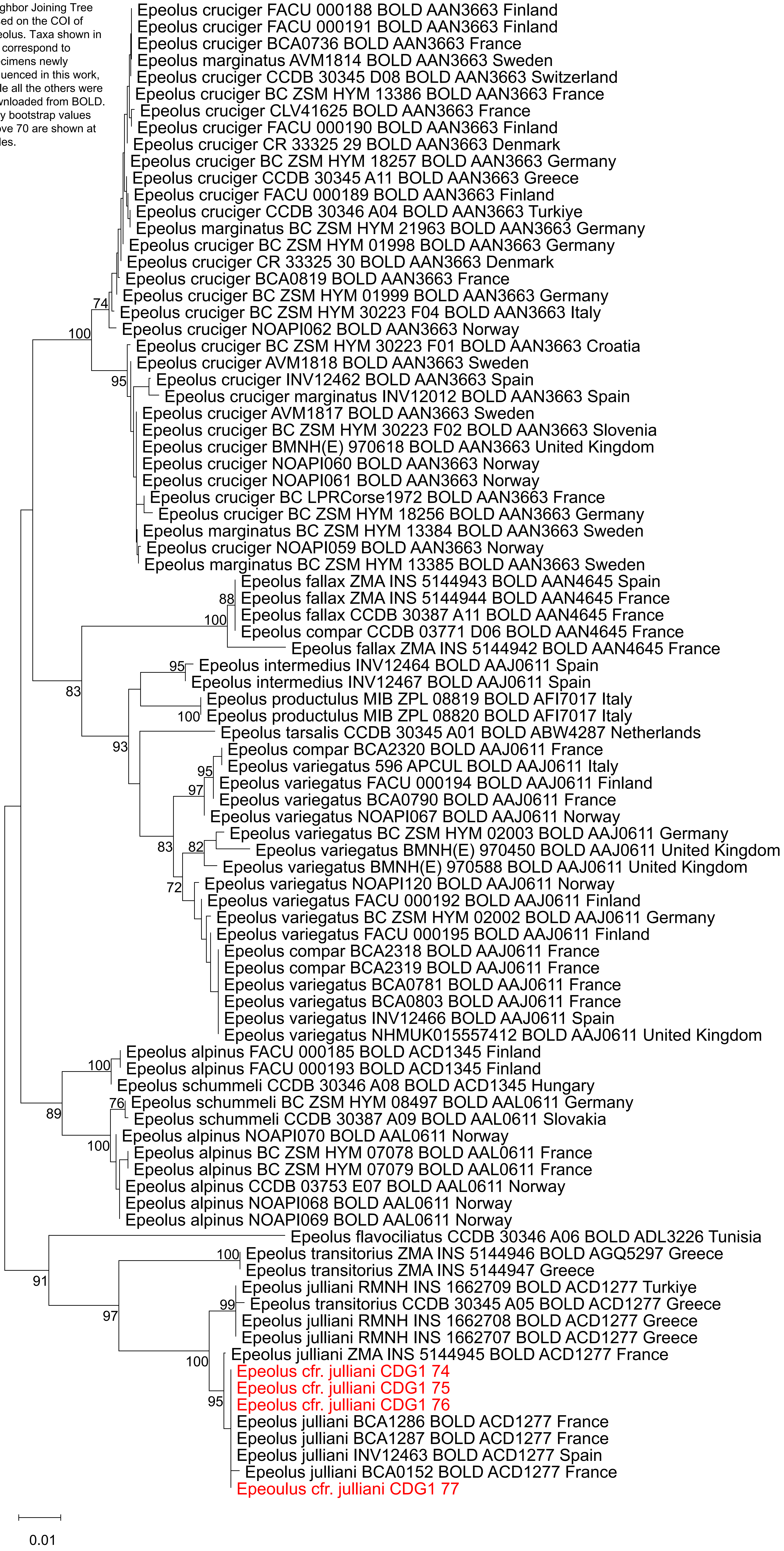

Fig. S4
Neighbor Joining Tree based on the COI of Melecta. Taxa shown in red correspond to specimens newly sequenced in this work, while all the others were downloaded from BOLD. Only bootstrap values above 70 are shown at nodes.

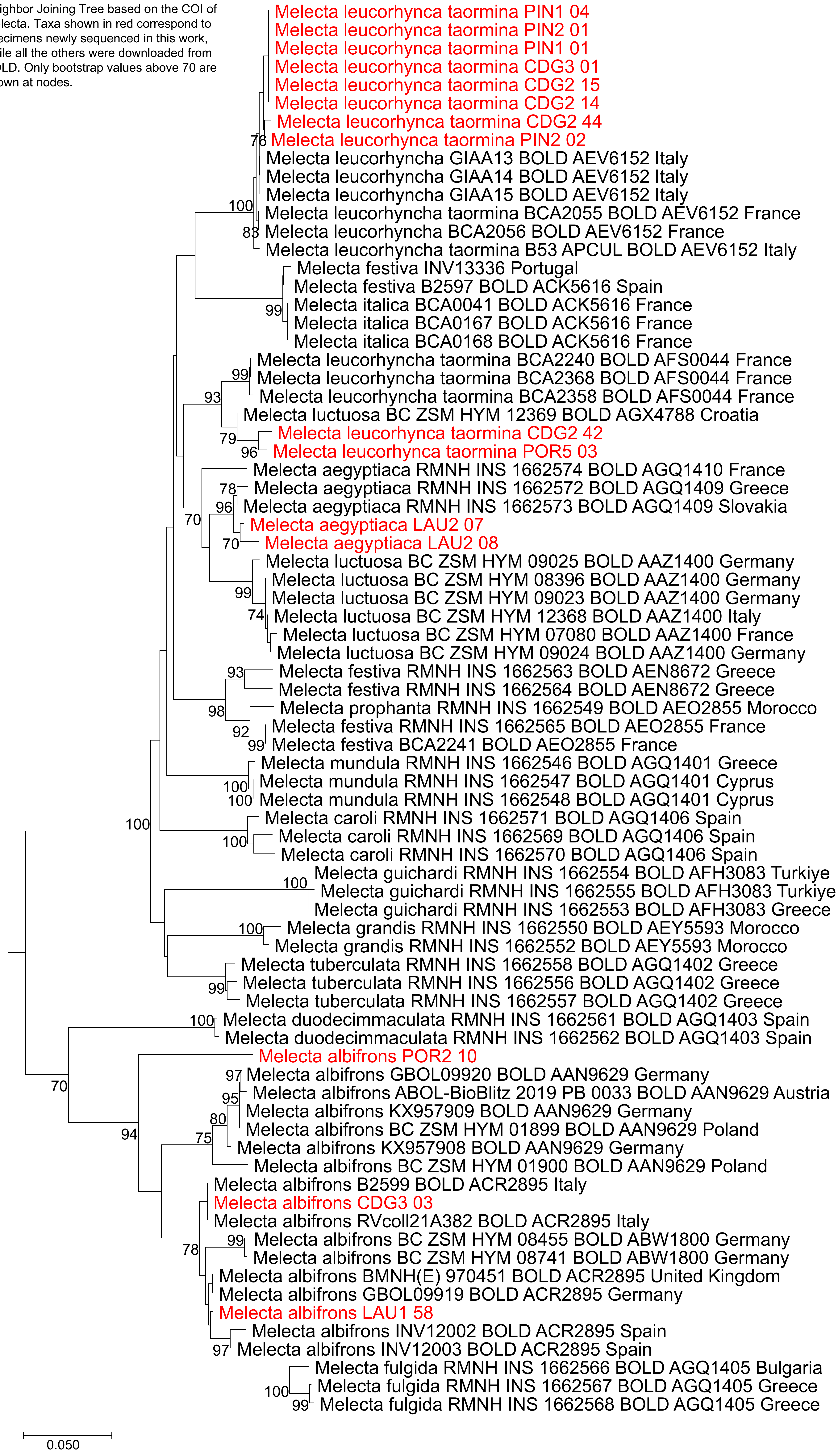

Fig. S5
Neighbor Joining Tree based on the COI of Nomada. Taxa shown in red correspond to specimens newly sequenced in this work, while all the others were downloaded from BOLD. Only bootstrap values above 70 are shown at nodes.

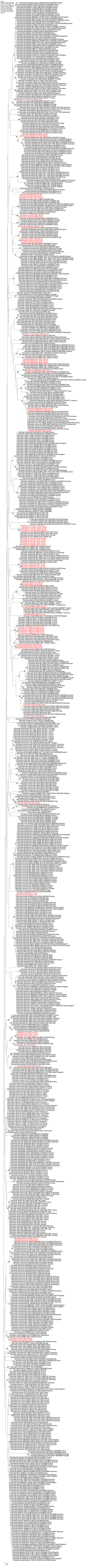

Fig. S6
Neighbor Joining Tree based on the COI of Sphecodes. Taxa shown in red correspond to specimens newly sequenced in this work, while all the others were downloaded from BOLD. Only bootstrap values above 70 are shown at nodes.

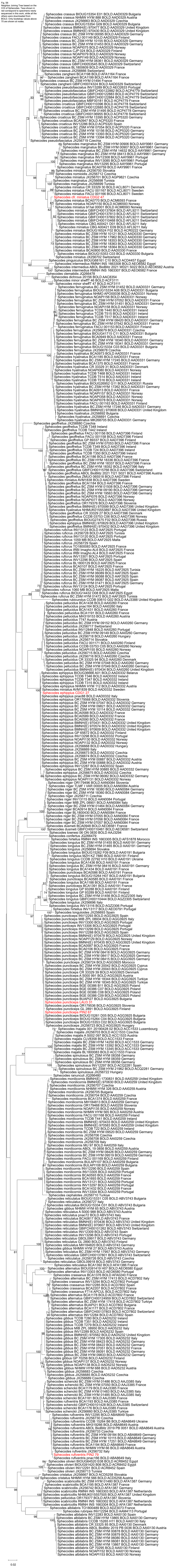

Supplement: Supplementary material 4 — Neighbor-Joining tree [file bdj-14-e193040-s004.pdf]
